# The DRAGON benchmark for clinical NLP

**DOI:** 10.1038/s41746-025-01626-x

**Published:** 2025-05-17

**Authors:** Joeran S. Bosma, Koen Dercksen, Luc Builtjes, Romain André, Christian Roest, Stefan J. Fransen, Constant R. Noordman, Mar Navarro-Padilla, Judith Lefkes, Natália Alves, Max J. J. de Grauw, Leander van Eekelen, Joey M. A. Spronck, Megan Schuurmans, Bram de Wilde, Ward Hendrix, Witali Aswolinskiy, Anindo Saha, Jasper J. Twilt, Daan Geijs, Jeroen Veltman, Derya Yakar, Maarten de Rooij, Francesco Ciompi, Alessa Hering, Jeroen Geerdink, Henkjan Huisman, On behalf of the DRAGON consortium, Joeran S. Bosma, Koen Dercksen, Luc Builtjes, Romain André, Christian Roest, Stefan J. Fransen, Constant R. Noordman, Mar Navarro-Padilla, Judith Lefkes, Natália Alves, Joey M. A. Spronck, Megan Schuurmans, Ward Hendrix, Witali Aswolinskiy, Anindo Saha, Jasper J. Twilt, Daan Geijs, Jeroen Veltman, Derya Yakar, Francesco Ciompi, Alessa Hering, Jeroen Geerdink, Henkjan Huisman, Max J. J. de Grauw, Leander van Eekelen, Bram de Wilde, Quintin van Lohuizen, Michelle Stegeman, Karlijn Rutten, Inge M. E. Smit, Gijs Stultiens, Christiaan G. Overduin, Matthieu J. C. M. Rutten, Ernst Th. Scholten, Rachel S. van der Post, Katrien Grünberg, Shoko Vos, Elise M. G. Taken, Iris D. Nagtegaal, Anne Mickan, Miriam Groeneveld, Paul K. Gerke, James A. Meakin, M. G. Looijen-Salamon, Tijmen L. M. de Haas, Fabian Hoitsma, Marina D’Amato, Maarten de Rooij

**Affiliations:** 1 Diagnostic Image Analysis Group, Department of Medical Imaging, Radboud University Medical Center, Nijmegen, The Netherlands; 2 Department of Health & Information Technology, Ziekenhuisgroep Twente, Almelo, The Netherlands; 3 Department of Radiology, Netherlands Cancer Institute, Amsterdam, The Netherlands; 4 Department of Radiology, University Medical Center Groningen, Groningen, The Netherlands; 5 Minimally Invasive Image-Guided Intervention Center, Department of Medical Imaging, Radboud University Medical Center, Nijmegen, The Netherlands; 6 Computational Pathology Group, Department of Pathology, Radboud University Medical Center, Nijmegen, The Netherlands; 7 Department of Radiology, Ziekenhuisgroep Twente, Almelo, The Netherlands; 8 Department of Medical Imaging, Radboud University Medical Center, Nijmegen, The Netherlands; 9 Department of Radiology, Jeroen Bosch Hospital, ’s-Hertogenbosch, The Netherlands; 10 Department of Pathology, Radboud University Medical Center, Nijmegen, The Netherlands

**Keywords:** Cancer, Diagnosis, Medical imaging, Research data

## Abstract

Artificial Intelligence can mitigate the global shortage of medical diagnostic personnel but requires large-scale annotated datasets to train clinical algorithms. Natural Language Processing (NLP), including Large Language Models (LLMs), shows great potential for annotating clinical data to facilitate algorithm development but remains underexplored due to a lack of public benchmarks. This study introduces the DRAGON challenge, a benchmark for clinical NLP with 28 tasks and 28,824 annotated medical reports from five Dutch care centers. It facilitates automated, large-scale, cost-effective data annotation. Foundational LLMs were pretrained using four million clinical reports from a sixth Dutch care center. Evaluations showed the superiority of domain-specific pretraining (DRAGON 2025 test score of 0.770) and mixed-domain pretraining (0.756), compared to general-domain pretraining (0.734, *p* < 0.005). While strong performance was achieved on 18/28 tasks, performance was subpar on 10/28 tasks, uncovering where innovations are needed. Benchmark, code, and foundational LLMs are publicly available.

## Introduction

Healthcare is under pressure due to a worldwide shortage of diagnostic personnel, such as radiologists and pathologists^1^. The demand for medical imaging is projected to increase substantially, worsening the diagnostic personnel shortage. For instance, worldwide cancer incidence is expected to increase by 47% from 2020 to 2040, resulting in substantially more imaging demand for the management of these patients^1,2^. This increasing imaging demand is unsustainable without innovative solutions^1^. Artificial Intelligence (AI) that enhances disease detection, guides treatment decisions, or optimizes follow-up care can potentially reduce workload. AI can serve as an assistive tool^3^ or function autonomously^4,5^, both requiring expert-level performance.

Large, high-quality, annotated datasets are essential to develop clinical algorithms with expert-level performance^6,7^. Routine clinical practice generates substantial datasets, with diagnostic interpretations and lab measurements described in clinical reports. However, the typically unstructured nature of these reports presents a challenge for efficient information extraction. Manual annotation of such data is time-consuming, costly, and requires specialized domain knowledge.

Natural Language Processing (NLP) has the potential to perform time-efficient, large-scale, and low-cost annotation of routine medical data, specifically using clinical reports. Large Language Models (LLMs) have shown groundbreaking text processing performance in the general language domain, but their application for automated data curation is underexplored. Most notably, (pre)training and evaluation of LLMs in the medical domain is constrained due to the limited availability of public datasets and benchmarks, which hinders systematic research on LLMs with clinical reports. Publicly available datasets with clinical reports are limited to high-resource languages (i.e., languages with many data resources) and a few institutes^8^. To the best of our knowledge, only the following datasets are publicly available: three chest X-ray radiology report datasets^9–11^ in English or Spanish; one chest CT radiology report dataset in English (translated from Turkish)^12^; one clinical notes dataset in English^13^; and one pathology reports dataset in English^14^.

Moreover, general text-based similarity metrics such as ROUGE can obfuscate clinical factuality, motivating the need for evaluating models using tasks with clinically-motivated evaluation metrics. Furthermore, pretraining of LLMs is key for good downstream performance, but optimal pretraining methods for clinical LLMs are understudied. Recent efforts have shown that general-purpose LLMs can be adapted to clinical tasks through fine-tuning, instruction tuning, and prompt engineering^15–19^. LLMs specifically pretrained for the medical domain exist (e.g. refs. ^20–23^), but the availability of medical domain-specific LLMs remains limited^24^, especially for non-high-resource languages.

This study has three objectives. First, introduce a unique benchmark with clinical reports from five Dutch care centers to provide a publicly available objective evaluation method. This benchmark also increases the number of languages with accessible clinical reports from two (English and Spanish) to three (adding Dutch), which opens avenues for research regarding languages that are less well-established within the field. Second, publicly release foundational LLMs pretrained using millions of clinical reports from a Sixth Dutch care center. Third, investigate three pretraining strategies for clinical LLMs in a non-high-resource language. This involves evaluating various architectures with these pretraining strategies using the benchmark.

Here we introduce the DRAGON (Diagnostic Report Analysis: General Optimization of NLP) challenge, focused on addressing the shortage of public resources for training and evaluating clinical NLP algorithms. Researchers from all over the world can benchmark their NLP algorithm using the DRAGON benchmark through the cloud-based Grand Challenge platform^25^. The DRAGON challenge aims to achieve a leap forward for clinical NLP algorithms, similar to how large-scale benchmarks have achieved this for other use cases^26–28^. To the best of our knowledge, the DRAGON challenge features the first large-scale benchmark for NLP algorithms using clinical reports. The benchmark is accessible online, where fully automatic performance assessment will be supported for at least 5 years. Furthermore, we publicly release foundational LLMs pretrained using four million clinical reports. The DRAGON challenge aims to accelerate research on NLP algorithms processing clinical reports, and in turn, facilitate automated dataset curation.

**Fig. 1:**
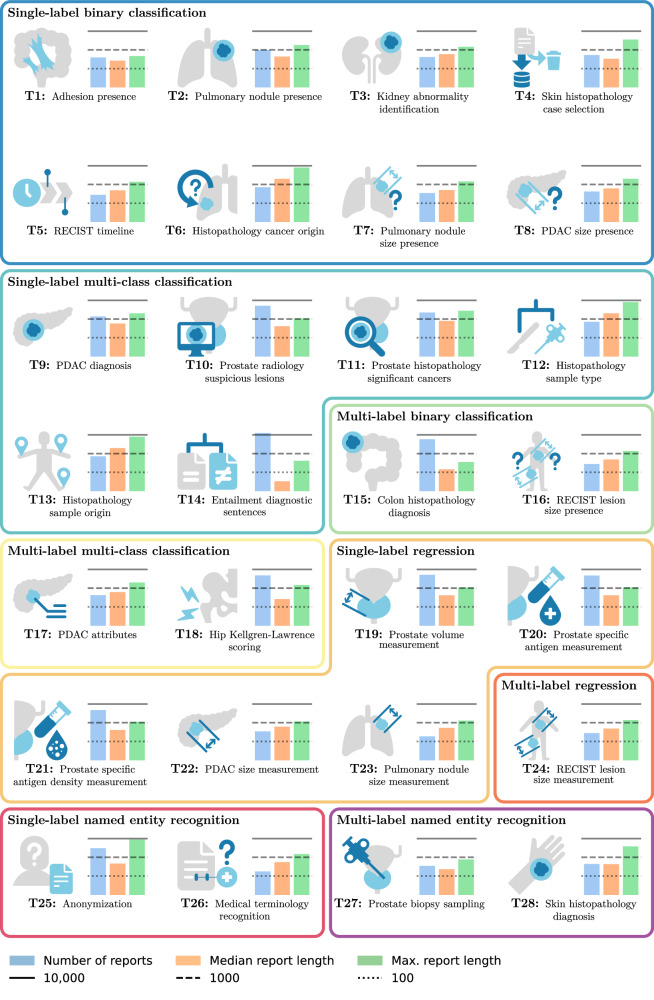
Overview of the tasks in the DRAGON benchmark. Tasks are grouped by their task type. For each task, key statistics are shown: (blue) the number of development cases, (orange) the median report length, and (green) the maximum report length. The report length is expressed in the number of tokens with an xlm-roberta-base tokenizer.

The benchmark comprises 28 clinically relevant tasks that are geared towards automatic dataset curation through the annotation of clinical reports (Fig. 1). The DRAGON benchmark focuses on classification, regression, and named entity recognition for automated dataset curation, rather than text-generation tasks. The tasks include selecting relevant studies, collecting key measurements, determining the clinical outcomes as labels, and more (see Table 1 for a full overview). The tasks include reports from multiple imaging modalities (MRI, CT, X-ray, histopathology) and many conditions spanning the entire body (lungs, pancreas, prostate, skin, etc.). The benchmark comprises 28,824 clinical reports from five Dutch centers. 24,021 reports were manually annotated, and an additional 4990 reports were automatically annotated as development data for one task. Most tasks use diagnostic reports (26/28), specifically radiology reports (19/26), pathology reports (7/26), or both (1/26). One task uses non-diagnostic clinical reports, and one task uses manually constructed clinical text. All tasks are categorized under one of eight task types (see Methods section Benchmark for details), allowing for the easy formulation of new tasks within these existing categories.

**Table 1:** Overview of tasks in the DRAGON benchmark

ID	Name	Task type	Metric	Number of development cases	Number of testing cases
T1	Adhesion presence	SL Bin Clf	AUROC	397	166
T2	Pulmonary nodule presence	SL Bin Clf	AUROC	1000	200
T3	Kidney abnormality identification	SL Bin Clf	AUROC	417	183
T4	Skin histopathology case selection	SL Bin Clf	AUROC	531	225
T5	RECIST timeline	SL Bin Clf	AUROC	278	119
T6	Histopathology cancer origin	SL Bin Clf	AUROC	715	304
T7	Pulmonary nodule size presence	SL Bin Clf	AUROC	348	66
T8	PDAC size presence	SL Bin Clf	AUROC	418	179
T9	PDAC diagnosis	SL MC Clf	Unweighted Kappa	1374	588
T10	Prostate radiology suspicious lesions	SL MC Clf	Linearly Weighted Kappa	5111	2229
T11	Prostate histopathology significant cancers	SL MC Clf	Linearly Weighted Kappa	2213	952
T12	Histopathology tissue type	SL MC Clf	Unweighted Kappa	707	304
T13	Histopathology tissue origin	SL MC Clf	Unweighted Kappa	718	297
T14	Entailment diagnostic sentences	SL MC Clf	Linearly Weighted Kappa	12,627	1422
T15	Colon histopathology diagnosis	ML Bin Clf	Macro AUROC	2748	1177
T16	RECIST lesion size presence	ML Bin Clf	AUROC	278	119
T17	PDAC attributes	ML MC Clf	Unweighted Kappa	418	179
T18	Hip Kellgren-Lawrence scoring	ML MC Clf	Unweighted Kappa	4803	172
T19	Prostate volume measurement	SL Reg	RSMAPES (ε = 4 cm^3^)	5138	2170
T20	Prostate specific antigen measurement	SL Reg	RSMAPES (ε = 0.4 ng/mL)	4759	2046
T21	Prostate specific antigen density measurement	SL Reg	RSMAPES (ε = 0.04 ng/mL^2^)	4700	2020
T22	PDAC size measurement	SL Reg	RSMAPES (ε = 4 mm)	343	147
T23	Pulmonary nodule size measurement	SL Reg	RSMAPES (ε = 4 mm)	186	32
T24	RECIST lesion size measurements	ML Reg	RSMAPES (ε = 4 mm)	278	119
T25	Anonymization	SL NER	Macro F1	3078	1307
T26	Medical terminology recognition	SL NER	F1	175	75
T27	Prostate biopsy sampling	ML NER	Weighted F1	349	146
T28	Skin histopathology diagnosis	ML NER	Weighted F1	439	185

*AUROC* area under the receiver operating characteristic curve, *SL* single-label, *ML* multi-label, *Bin* binary, *MC* multi-class, *Clf* classification, *Reg* regression, *NER* named entity recognition, *RSMAPES* Robust Symmetric Mean Absolute Percentage Error Score, *RECIST* response evaluation criteria in solid tumors, *PDAC* pancreatic ductal adenocarcinoma.

All data, including clinical reports and associated labels, are securely stored in a sequestered manner. This prevents users from directly accessing or viewing the data, preserving patient privacy by design. While participants cannot directly download or see the data, they do have full functional access for model training and validation through the platform interface. Keeping the test labels hidden helps to mitigate potential biases. To aid the development of solutions we provide synthetic datasets for all task types and provide an example case for each of the tasks in Supplementary Note 7.

**Fig. 2:**
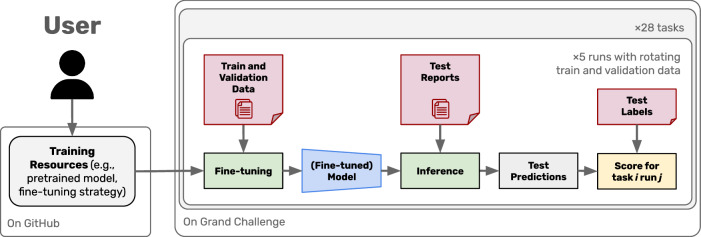
Workflow for the DRAGON benchmark. Challenge participants must provide all resources necessary to process the reports and generate predictions for the test set. Processing of reports is performed on the Grand Challenge platform, without any interaction with the participant.

Execution of the benchmark is performed on the Grand Challenge platform, with the workflow illustrated in Fig. 2. Clinical NLP methods are defined as all resources for training and inference (e.g., pretrained model, fine-tuning strategy), and referred to as the algorithm. For each task, a test set (without labels), training set, and validation set are available to the algorithm. The training set enables fine-tuning of a model or the realization of few-shot approaches. The validation set may be used to perform model selection, but not as additional training data. Participants are encouraged to define general rules to define the hyperparameters (e.g., using the number of training samples to define the number of training steps, using the distribution of labels to define the normalization approach, using the class distribution to inform sampling strategy, etc.). To assess the model fine-tuning robustness, the training and validation sets rotate using five-fold cross-validation, without patient overlap between splits.

Predictions for each task are evaluated using a primary metric depending on the task type (see Table 1 for a full list, and Supplementary Note 1 for the motivation). To characterize the overall performance of a clinical NLP algorithm, we introduce the DRAGON 2025 test score, where a value of 0 indicates no clinical utility and a value of 1 indicates a perfect match with the manual annotations. The DRAGON 2025 test score is the unweighted average of the primary metric across all 28 tasks and each of the five training runs per task.

Researchers worldwide may request to conduct statistical comparisons like those presented in this study for their own research purposes. These requests must be accompanied by a well-defined statistical analysis plan. To foster open science and ensure the verifiability of confirmatory analyses, both the statistical plans and their corresponding outcomes will be made publicly available.

Complete reproducibility of submissions is ensured by performing all model fine-tuning and inference activities on the Grand Challenge platform. This verifies that all required code and associated training resources are provided. To promote cumulative research progress, we also require that all user’s training resources and a description of the method be made publicly available, possibly after a grace period designated for the publication of the related research paper.

To quantify the reliability of annotations used in the DRAGON benchmark, we conducted a reader study to assess inter-annotator agreement, providing a conservative estimate of annotation quality.

## Results

Pretraining is key for obtaining high performance with LLMs^29^. Foundational LLMs must adapt to diverse tasks, and variability in clinical reporting styles makes this challenging. Reporting styles can differ based on the region, hospital, diagnostic task, and even individual physician preference. Additionally, there is a difference in the type of cases seen across care centers. Large academic centers have more tertiary referrals with complex pathology, whereas non-academic hospitals typically see a larger number of common, less complex cases. Moreover, high-resource languages (English, Spanish, and Chinese) dominate the available pretrained foundational LLMs, but two-thirds of countries use non-high-resource languages^30^. In the context of clinical NLP, pretraining can be done using general-domain data (incorporating sources like Wikipedia and books), domain-specific data (utilizing clinical narratives such as radiology and pathology reports), or mixed-domain data (a combination of general-domain and domain-specific pretraining). Related literature compared the efficacy of pretraining strategies for biomedical downstream tasks with PubMed abstracts and showed that domain-specific pretraining outperformed both general-domain pretraining and mixed-domain pretraining^31^. For the clinical domain, contradicting results have been reported. Yan et al.^21^ found that mixed-domain pretraining outperformed general-domain pretraining, Rietberg et al.^32^ found that general-domain pretraining outperformed domain-specific pretraining, and Verkijk et al.^22^ found that domain-specific pretraining outperformed general-domain and mixed-domain pretraining. Each comparison was based on only one to three tasks, limiting generalizability. Which pretraining strategy is optimal for clinical NLP is therefore still unknown, especially for non-high-resource languages.

**Fig. 3:**
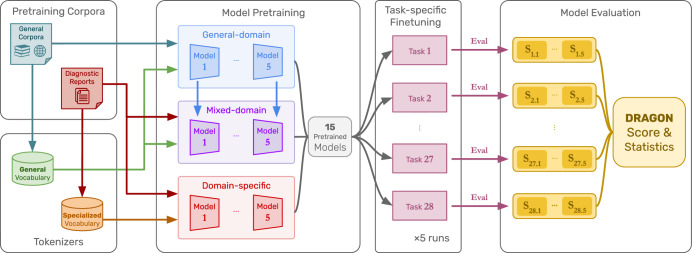
Experimental setup to compare pretraining strategies. Several LLM architectures are pretrained using either general-domain, domain-specific, or mixed-domain pretraining (general-domain followed by domain-specific pretraining). Each of the resulting pretrained foundational models is evaluated on the DRAGON benchmark by task-specific fine-tuning followed by performance evaluation on the test set. To assess fine-tuning stability, the training and validation datasets rotate with five-fold cross-validation, resulting in five performance assessments for each of the 28 tasks per pretrained model.

To investigate the optimal pretraining strategy, we conducted a comparative analysis of general-domain, domain-specific, and mixed-domain pretraining. Each strategy was applied to five LLM architectures that are commonly used and can be implemented on hospital infrastructure using a single GPU (24 GB VRAM), allowing the preservation of patient privacy by design. These LLMs are evaluated on the DRAGON benchmark, with the experimental setup depicted in Fig. 3.

For general-domain pretraining, we utilized models from the HuggingFace Model Hub. Language-specific versions were selected according to availability, which mirrors the typical development scenario for clinical NLP in languages with varying levels of resource availability. The selected models were Dutch BERT base^29^, multilingual RoBERTa base and large^33^, and English Longformer base and large^34^.

For domain-specific and mixed-domain pretraining, we used four million clinical reports from a single Dutch non-academic care center (different from the five centers used in the benchmark). This approach allows us to rigorously investigate how the model adapts to diverse reporting styles and patient cohorts across multiple care settings, assessing its robustness and generalizability in real-world scenarios. Pretraining was done using the masked language modeling (MLM) objective, with hyperparameters for domain-specific and mixed-domain pretraining based on Gu et al.^31^.

**Fig. 4:**
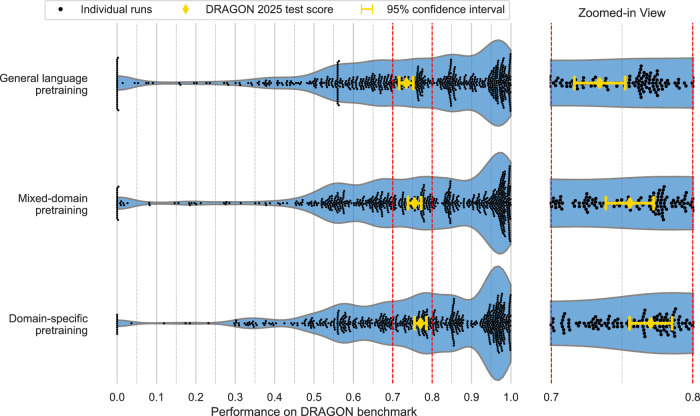
Benchmark results. Performance observed across each architecture, task, and training run in the DRAGON benchmark for the three pretraining strategies: (1) general-domain pretraining, (2) mixed-domain pretraining, and (3) domain-specific pretraining. Performance metrics from individual fine-tuning runs are shown as black dots (from 5 architectures, 28 tasks, and 5 runs, resulting in 700 scores per pretraining method). The diamond and error bars show the DRAGON 2025 test score (average of the score from each run) and its 95% confidence interval. The blue shading represents the density estimation of individual scores in a violin plot.

Pretraining on millions of clinical reports resulted in significantly better performance for LLMs compared to general-domain pretraining, as evaluated on the DRAGON benchmark, with LLMs represented by the BERT, RoBERTa (base and large), and Longformer (base and large) architectures (Fig. 4). We report the DRAGON 2025 test score (Eq. (2)) with a 95% confidence interval (CI). Domain-specific pretraining improved the baseline score to 0.770 (95% CI 0.755–0.785, *p* = 0.004), and mixed-domain pretraining improved the baseline score to 0.756 (95% CI 0.739–0.773, *p* = 0.004), both compared to general-domain pretraining (0.734, 95% CI 0.717–0.752). No statistically significant performance difference was observed between domain-specific pretraining and mixed-domain pretraining (*p* > 0.05). The highest performance on the DRAGON benchmark was observed for RoBERTa large with domain-specific pretraining, obtaining a DRAGON 2025 test score (Eq. (1)) of 0.819 (95% CI 0.793–0.844). Performance for each model architecture and pretraining strategy across each task is provided in Supplementary Note 2 and 3.

Model performance for the best model (RoBERTa large with domain-specific pretraining) was excellent for 10/28 tasks (T1, T2, T4, T5, T10, T15, T16, T19, T20, T21), good for 8/28 tasks (T3, T7, T11, T22, T23, T25, T26, T27), moderate for 6/28 tasks (T9, T13, T17, T18, T24, T28) and poor for 4/28 tasks (T6, T8, T12, T14), based on pre-defined performance thresholds described in Supplementary Note 1. For all three lesion size extraction tasks (T22, T23, T24), the best model extracted the reported lesion size within 1 mm for only 7% to 18% of lesions. Similarly, the model extracted the reported lesion size within 5 mm for only 33–61% of lesions. These tasks had a primary metric of 0.782–0.854 for the best model, placing these tasks in “moderate” or “good” as per the pre-defined performance thresholds, motivating an update of the thresholds for interpretation of this metric in future work.

The reader study demonstrated strong overall inter-annotator agreement, achieving an average Krippendorff’s alpha of 0.859 across classification and regression tasks (T1–T24) and an average F1 score of 0.860 for named entity recognition tasks (T25–T28). Accuracy relative to the original annotations was consistently high (≥90%) for most tasks. However, higher variability was observed in histopathology cancer origin (T6, α = 0.333), entailment of diagnostic sentences (T14, α = 0.550), and Kellgren-Lawrence scoring (T18, α = 0.557). Tasks T06, T14 and T18 are inherently more complex, necessitating extensive diagnostic background knowledge and specialized training, which were available during the original annotation process but impractical within the reader study. The improved agreement achieved by RoBERTa large with domain-specific pretraining on several tasks (e.g., T18 with α = 0.70 ± 0.04), indicates that the lower agreement observed in the reader study likely stems from limited reader training compared to the original annotators. Thus, the results presented here provide a conservative estimate of annotation reliability within the DRAGON benchmark. Detailed task-level results are provided in Supplementary Note 5.

## Discussion

This study had three objectives. First, the DRAGON benchmark provides a comprehensive performance assessment of clinical NLP algorithms for researchers worldwide. The benchmark includes a wide variety of tasks aimed at automated dataset curation. Large-scale manual annotations are used to evaluate LLMs with diagnostically relevant metrics, rather than general text-based similarity metrics that can obfuscate clinical correctness. Second, pretrained foundational LLMs are released on HuggingFace, and code for preprocessing, pretraining, fine-tuning, inference, evaluation, and statistical evaluation is shared on GitHub. All resources are FAIR (Findable, Accessible, Interoperable, Reusable) and publicly accessible at dragon.grand-challenge.org. The end-to-end pipeline for task-specific fine-tuning and prediction of unlabeled reports is also accessible on Grand Challenge, allowing users to utilize, validate, and reproduce these algorithms without any setup. Additionally, the full pipeline can be deployed offline, allowing reports to remain on-premises. Third, results show superior performance for models that are pretrained using domain data, compared to pretraining exclusively on general-domain text.

Model performance for the best model (RoBERTa large with domain-specific pretraining) was good to excellent for 18/28 tasks (based on pre-defined performance thresholds described in Supplementary Note 1). Model performance was poor for tasks T6, T8, T12, and T14. Tasks T6 (histopathology cancer origin), T8 (pancreatic ductal adenocarcinoma size presence), and T12 (histopathology sample origin) have highly unbalanced labels (the majority class occurs 4.6–9.2 times as often as the minority class), which could explain the reduced performance. Task T14 (entailment diagnostic sentences) uses constructed medical sentences translated from English. For this downstream task, the domain gap is relatively large from the domain data with full-text reports, which could explain the poor performance.

For the regression tasks, the extraction of lesion size (tasks T22, T23, and T24) performed poorly, while the extraction of other measurements (tasks T19, T20, and T21) performed well. The tokenization process, where words in a report are embedded as a sequence of vectors, complicates the model’s ability to accurately interpret numeric information. This challenge arises because the numeric context and precision are often lost during tokenization, making it difficult for the model to correctly associate specific tokens with their corresponding numeric values. The performance disparity between these two groups of tasks may be attributed to the substantial difference in development dataset sizes, with the latter having approximately 18 times more cases. Novel NLP techniques that more effectively handle this type of information extraction would significantly enhance automatic dataset curation.

Researchers worldwide are encouraged to contribute and build upon the baseline results. The requirement for open-source submissions (upon paper acceptance) ensures that each contribution not only stands on its own but also propels the entire field forward, allowing developers to iteratively build on each other’s work. All algorithms, including those of participants, will be made accessible through the Grand Challenge platform, such that improved algorithms directly allow higher-quality dataset curation.

Previous studies that explored the application of NLP in healthcare evaluated models either on a few clinical tasks or with only a few full-length medical reports^35,36^. The DRAGON challenge substantially increases the scope of clinical tasks and the number of accessible full-length medical reports, thereby enhancing the evaluation capabilities crucial for steering the development of clinical LLMs. Our findings support the hypothesis that domain-specific pretraining enhances model performance, a notion that has been suggested before, but not validated on this scale in prior research. This positions our study as a significant advancement in the field, providing strong empirical evidence based on which the field can move forward.

The tasks in the benchmark are aimed at automated dataset curation, such that advances result in large, high-quality, annotated datasets. These datasets are crucial for developing medical image analysis algorithms to improve patient management. Leveraging AI is essential to improve patient management and address the growing demand for medical imaging. Additionally, automated report analysis allows the investigation of trends, such as the number of incidental findings^37^. Results showed that present-day algorithms can annotate clinical reports with excellent or good performance for 18/28 tasks. Researchers can now use algorithms in their data curation workflows to enhance global research quality, with the DRAGON challenge providing the knowledge of which tasks are feasible now and in the future as new submissions come in.

Our study has limitations. Many of the tasks are sourced from a single academic tertiary care center (14/28, 50%). Additionally, the medical reports are accessible in sequestered form such that algorithms can interact with them but are not visible to algorithm developers. Although example reports are made available, algorithm development could be more effective if all development data were directly available. On the other hand, this setup does push developers to devise more general solutions. Furthermore, most reports were annotated by trained investigators under the supervision of clinical experts in regular research settings, which were non-controlled environments. This likely resulted in some label noise, mirroring the realities of data collection in the real world and ensuring that training algorithms can accommodate it. However, for the test sets, it would be ideal to have no label noise. No inter-annotator agreement was assessed during the original data collection, preventing us from quantifying label noise. We took extensive measures to ensure consistency—such as annotation protocols, annotator training, clinical expert supervision, and dataset-wise sanity and consistency checks. The quality and consistency of the annotations was verified in a retrospective reader study to quantify the inter-annotator agreement. This reader study showed a generally high inter-annotator agreement. In future work, we plan to systematically revisit a subset of reports, specifically where top-performing models disagree with the manual annotations. This targeted effort will yield further insights into human and model performance and guide improvements in annotation quality and benchmark utility. Additionally, while our benchmark focused on closed-ended questions, the inclusion of generative tasks, such as free-text generation evaluated using Unified Medical Language System (UMLS)-based metrics, could have provided a broader assessment of model capabilities. We encourage data contributions; such that future iterations of the DRAGON challenge can address these limitations.

Based on our findings and limitations, several avenues for future research emerge. First, algorithmic improvements are necessary to tackle datasets with highly unbalanced labels and perform regression tasks with limited annotated data. Second, improving data efficiency when fine-tuning the tasks would unlock automated report analysis for even more clinical settings. Third, future studies could focus on improved pretraining strategies that leverage the nature of electronic healthcare records, where additional clinically relevant metadata is typically available in a structured way. Fourth, federated strategies are necessary to further scale up pretraining, such that data from multiple hospitals can be used while preserving patient privacy. Lastly, investigating related research questions, such as the application of these models in real-time clinical decision support, could open new frontiers in the integration of AI in healthcare.

The DRAGON challenge presents a benchmark for NLP algorithms using clinical reports. The benchmark contains a wide variety of clinically relevant tasks and provides exceptional evaluation capabilities to advance the field. Results showed the superiority of models pretrained on clinical reports, compared to models pretrained exclusively on general language texts. Benchmark, code, and pretrained foundational LLMs are publicly available, to accelerate the development of accurate and robust clinical NLP algorithms.

## Methods

### Data collection and preprocessing

For the DRAGON benchmark, 28,824 clinical reports (22,895 patients) were included from five Dutch care centers (Radboud University Medical Center, Jeroen Bosch Ziekenhuis, University Medical Center Groningen, Rijnstate, and Antoni van Leeuwenhoek Ziekenhuis) of patients with a diagnostic or interventional visit between 1 January 1995 and 12 February 2024. For 27/28 tasks, all reports were manually annotated. For task 18, the 4803 development cases were automatically annotated using GPT-4, and the 172 testing cases were manually annotated. Characteristics of the benchmark datasets are summarized in Fig. 1 and Table 1, and individual dataset details are provided in Supplementary Note 7.

For pretraining, 4,333,201 clinical reports (466,351 consecutive patients) were selected from Ziekenhuisgroep Twente from patients with a diagnostic or interventional visit between 13 July 2000 and 25 April 2023. 180,439 duplicate clinical reports (179,808 patients) were excluded, resulting in 4,152,762 included reports (463,692 patients). These reports were split into training (80%, 3,322,209 reports), validation (10%, 415,276 reports), and testing (10%, 415,277 reports). The testing reports were set aside for future analysis and are not used in this study.

This study was approved by the institutional or regional review board of each participating center (Radboud University Medical Center: CMO 2016–3045; Jeroen Bosch Ziekenhuis: A21-0554-05; University Medical Center Groningen: IRB 2018–597, Rijnstate: METC Oost-Nederland, registration number 19082; Antoni van Leeuwenhoek Ziekenhuis: IRBd22-159; Ziekenhuisgroep Twente: ZGT25-04). Informed consent was exempted given the retrospective scientific use of deidentified patient data.

Reports utilized in the benchmark are stored in sequestered form (accessible only to algorithms). Protected health information (PHI) was detected automatically using institutional PHI detection software (see Supplementary Note 6 for details). For a subset of reports, the detected PHI was manually refined. Reports were anonymized by replacing the PHI with realistic surrogates^38^. Additionally, a subset of reports was manually anonymized by removing PHI. Reports were converted to ASCII characters (e.g., replacing ë with e) during the anonymization procedure.

### Benchmark

The DRAGON benchmark serves as an extensive resource for testing and advancing clinical NLP algorithms, particularly in the realm of automated data curation. In the context of medical imaging datasets, data curation involves selecting relevant studies, collecting key measurements, and determining the clinical outcomes as labels. Clinical reports are the primary source for these curation tasks. The DRAGON benchmark aims to catalyze the development of algorithms capable of addressing a broad spectrum of data curation tasks and introduces 28 clinically relevant tasks, an overview of which is given in Table 1. To facilitate handling many tasks, eight distinct task types were identified and defined. These task types were designed to be universally applicable, and most data curation tasks should be able to be formulated as one of these tasks:

1. single-label binary classification (e.g., classify reports as indicating “cancer” or “no cancer”),
2. single-label multi-class classification (e.g., classify reports based on the type of diagnosis, such as “cancer”, “other disease”, or “benign”),
3. multi-label binary classification (e.g., classify presence of multiple conditions, such as “hyperplastic polyps”, “high-grade dysplasia”, and “cancer”, where each condition is treated as a binary indicator),
4. multi-label multi-class classification (e.g., classify reports by multiple factors, such as disease severity “mild”, “moderate”, or “severe” and urgency of treatment “low”, “medium”, or “high”),
5. single-label regression (e.g., predict the prostate volume described in the report),
6. multi-label regression (e.g., predict the lesion diameter of multiple lesions),
7. single-label named entity recognition (e.g., identify and classify protected health information in a report, such as names, dates, and places), and
8. multi-label named entity recognition (e.g., identify multiple types of entities in a medical report, where entities can be diseases, symptoms, treatments, and test results, potentially with overlap and multiple occurrences).

All reports included in this benchmark are written in Dutch. This aspect necessitates the development of pretraining and fine-tuning strategies that are effective for a language with fewer resources. This enhances the global applicability of these algorithms, particularly in non-English, non-Spanish, and non-Chinese settings, while they still maintain their relevance in high-resource language environments.

The medical reports encompass a wide variety of writing styles, which are influenced by factors such as the specific hospital, regional practices, and individual physician preferences. Furthermore, the nature and complexity of cases vary across hospitals, with academic centers often dealing with more complex pathologies. To assess the generalizability of pretrained clinical LLMs across different hospitals, we established the benchmark with reports from various external academic and non-academic centers. However, the pretraining dataset was compiled from a single non-academic hospital. This aligns with the commonly feasible setup, where the large-scale sharing of reports across centers is restricted due to concerns regarding patient privacy.

Submissions to the DRAGON challenge should become available to all researchers such that the whole research field moves forward. To achieve this, all submissions must become open source and should be accompanied by a document detailing the methodology used. Open source in this context means that all resources are accessible to the public and that other scientists can exactly replicate the method. To address potential conflicts with academic processes, such as the submission of papers to journals, submissions are not required to be open source immediately. A grace period of up to six months is permitted, with potential extensions available upon justified request (for example, if the manuscript is still under review). These requirements are designed to ensure that high-performing contributions are shared with the broader research community in a timely manner.

### Evidence synthesis

Each task was evaluated using a primary metric that was also used for statistical testing. Additional metrics for some tasks are shared in Supplementary Note 3, to facilitate comparison with previously published results. The primary metric for each task was chosen to reflect clinically relevant performance assessment. The chosen metrics are listed in Table 1, and their choice is motivated in Supplementary Note 1. The DRAGON 2025 test score is used as the ranking score for the challenge leaderboard and is the average of the primary metric for each of the tasks, and each of the runs. A score of 0 indicates no clinical utility and a score of 1 indicates a perfect match with the manual annotations. The arithmetic mean was chosen for its simplicity and ease of understanding, even though it does not account for the differences in ranges for different metrics, nor differences in typical ranges between tasks. With ${S}_{{ij}}$ the metric for task $i$ run $j$, the DRAGON 2025 test score is calculated for pretrained models as:

1 $${S}_{{DRAGON}}=\frac{1}{140}\mathop{\sum }\limits_{i}^{28}\mathop{\sum }\limits_{j}^{5}{S}_{{ij}}$$

For pretraining methods, where the performances of five architectures are pooled, the DRAGON 2025 test score is calculated with ${S}_{{ijk}}$ the metric for task $i$ run $j$ architecture $k$ as:

2 $${S}_{{DRAGON}}=\frac{1}{700}\mathop{\sum }\limits_{i}^{28}\mathop{\sum }\limits_{j}^{5}\mathop{\sum }\limits_{k}^{5}{S}_{{ijk}}$$

Within the manuscript, the DRAGON 2025 test score (Eqs. (1) or (2)) is reported with the 95% CI obtained from the scores of the individual runs. For pretrained models, the 140 scores obtained from the individual runs are used, while for pretraining methods the 700 scores from individual runs are used. These scores are directly used for n-out-of-n bootstrapping with 10,000 iterations to determine the 95% CI. In Supplementary Note 2 and 3, where the performance is provided for each task across the pretraining method and architecture, the average score and standard deviation across the five runs are given.

### Experiment

Our experiment compares the efficacy of three distinct pretraining strategies: general-domain pretraining, domain-specific pretraining, and mixed-domain pretraining (a hybrid approach involving initial general-domain pretraining followed by domain-specific fine-tuning). The DRAGON benchmark with the DRAGON 2025 test score (Eq. (2)) served as our metric for evaluating the performance of these strategies. To generalize the results across architectures, we pretrained five popular architectures and pooled the performance metrics.

Considering the importance of maintaining patient confidentiality when processing clinical reports, our model architecture selection was informed by the ability to train in single-GPU environments. This allows the full end-to-end model training and application pipeline to remain within the hospital infrastructure, thereby safeguarding patient privacy by design. The architectures incorporated into our study were selected from the HuggingFace Model Hub and are BERT base (GroNLP/bert-base-dutch-cased)^39^, RoBERTa base (xlm-roberta-base)^33^, RoBERTa large (xlm-roberta-large)^33^, Longformer base (allenai/longformer-base-4096)^34^, and Longformer large (allenai/longformer-large-4096)^34^. These models were pretrained using general-domain data (Dutch for BERT base, multilingual for RoBERTa, and English for Longformer), as specified in the associated publications. These architectures are widely used and able to operate within the specified computational constraints.

Acknowledging the prevalent challenge of resource scarcity in non-English language settings, our experiment utilized pretraining configurations in Dutch, multilingual, and English. This selection mirrors the typical scenario in low-resource contexts, where a pretrained model in the target language may not be available, necessitating the utilization of either multilingual models or models pretrained in alternative languages.

Pretraining was performed using the masked language modeling (MLM) objective^29^. The HuggingFace implementation for MLM was used, where 15% of the tokens were masked. Pretraining was performed for 3 epochs when following general-domain pretraining and for 10 epochs when pretraining from scratch. All parameters are detailed in their respective model card^40–49^. Convergence of the pretraining runs was verified using the validation set. If divergence occurred, the pretraining run was rolled back to the last checkpoint before divergence, and pretraining was continued. The MLM pretraining technique was chosen following Liu et al.^33^, since it represents the standard pretraining technique.

For the primary analysis, we pooled the results from each architecture to compare the three pretraining methods. The overall efficacy of a pretraining approach is calculated as the DRAGON 2025 test score, Eq. (2). This comprehensive evaluation framework enables a nuanced understanding of the relative advantages of each pretraining strategy in the context of clinical report analysis.

### Reader study

To assess annotation consistency within the DRAGON benchmark, we conducted a reader study evaluating inter-annotator agreement. Tasks with similar annotation requirements were grouped. For each of the 16 reader study blocks, we randomly sampled 30–50 reports, resulting in 550 unique reports. Each block was independently annotated by two readers. Reader assignment ensured that neither reader had previously annotated that specific task, thus allowing comparison among three independent annotations per report: two from the reader study and the original benchmark annotation. Due to practical constraints, the comprehensive training, clinician consultation, and post-annotation consistency checks of the original annotations were not replicated, making the inter-annotator agreement presented a conservative estimate of the benchmark’s annotation reliability.

Agreement was quantified using Krippendorff’s alpha for classification and regression tasks (T1–T24) and F1 score for named entity recognition tasks (T25–T28). Additionally, we assessed annotation accuracy by comparing the readers’ annotations directly against the original benchmark labels. Additional details are provided in Supplementary Note 5.

### Statistical analysis

The performance of domain-specific pretraining, general-domain pretraining, and mixed-domain pretraining were statistically compared. We evaluated the performance of domain-specific pretraining in comparison to general-domain pretraining, mixed-domain pretraining in comparison to general-domain pretraining, domain-specific pretraining in comparison to mixed-domain pretraining, and mixed-domain pretraining in comparison to domain-specific pretraining. Statistical tests were reserved for these four primary outcomes only. To maintain the type I error while investigating multiple comparisons, all study objectives were prespecified in a hierarchical family tree and tested accordingly^50^. Multiplicity was corrected for at each stage using the Holm–Bonferroni method, considering a base alpha value of 0.05. See the Statistical Analysis Plan in Supplementary Note 4 for more details.

## Supplementary information

Supplementary Information

## Data Availability

All data used in the benchmark is publicly accessible through the Grand Challenge cloud platform. Data is not directly accessible, but rather accessible to the submitted algorithms. Data used to pretrain the foundational models is not available. The pretrained foundational models are all available on HuggingFace, see https://dragon.grand-challenge.org/pretrained-models.

## Code availability

All code for this study is publicly available. The most up-to-date code is available on GitHub at https://github.com/DIAGNijmegen/dragon_baseline for the baseline algorithm, https://github.com/DIAGNijmegen/dragon_eval for the evaluation method, https://github.com/DIAGNijmegen/dragon_prep for data preparation, and http://github.com/DIAGNijmegen/dragon_submission for a template for new submissions. A snapshot of the code used for this study is available on Zenodo at 10.5281/zenodo.13695131.

## References

[1] Hricak, H. et al. Medical imaging and nuclear medicine: a Lancet Oncology Commission. *Lancet Oncol.* **22**, e136–e172 (2021). 33676609 10.1016/S1470-2045(20)30751-8 PMC8444235

[2] Sung, H. et al. Global Cancer Statistics 2020: GLOBOCAN Estimates of Incidence and Mortality Worldwide for 36 Cancers in 185 Countries. *CA A Cancer J. Clinicians* **71**, 209–249 (2021). 10.3322/caac.21660 33538338

[3] Netzer, N. et al. Fully Automatic Deep Learning in Bi-institutional Prostate Magnetic Resonance Imaging: Effects of Cohort Size and Heterogeneity. *Invest. Radio.* **56**, 799–808 (2021). 10.1097/RLI.0000000000000791 34049336

[4] Lång, K. et al. Artificial intelligence-supported screen reading versus standard double reading in the Mammography Screening with Artificial Intelligence trial (MASAI): a clinical safety analysis of a randomised, controlled, non-inferiority, single-blinded, screening accuracy study. *Lancet Oncol.* **24**, 936–944 (2023). 37541274 10.1016/S1470-2045(23)00298-X

[5] Martin, D. D., Calder, A. D., Ranke, M. B., Binder, G. & Thodberg, H. H. Accuracy and self-validation of automated bone age determination. *Sci. Rep.* **12**, 6388 (2022). 35430607 10.1038/s41598-022-10292-y PMC9013398

[6] Gulshan, V. et al. Development and Validation of a Deep Learning Algorithm for Detection of Diabetic Retinopathy in Retinal Fundus Photographs. *JAMA* **316**, 2402–2410 (2016). 27898976 10.1001/jama.2016.17216

[7] Hosseinzadeh, M. et al. Deep learning–assisted prostate cancer detection on bi-parametric MRI: minimum training data size requirements and effect of prior knowledge. *Eur. Radio.* **32**, 2224–2234 (2022). 10.1007/s00330-021-08320-y PMC8921042 34786615

[8] Spasic, I. & Nenadic, G. Clinical text data in machine learning: systematic review. *JMIR Med. Inf.* **8**, e17984 (2020). 10.2196/17984 PMC7157505 32229465

[9] Johnson, A. E. W. et al. MIMIC-CXR, a de-identified publicly available database of chest radiographs with free-text reports. *Sci. Data* **6**, 317 (2019). 31831740 10.1038/s41597-019-0322-0 PMC6908718

[10] Bustos, A., Pertusa, A., Salinas, J.-M. & De La Iglesia-Vayá, M. PadChest: a large chest x-ray image dataset with multi-label annotated reports. *Med. Image Anal.* **66**, 101797 (2020). 32877839 10.1016/j.media.2020.101797

[11] Demner-Fushman, D. et al. Preparing a collection of radiology examinations for distribution and retrieval. *J. Am. Med. Inform. Assoc.* **23**, 304–310 (2016). 26133894 10.1093/jamia/ocv080 PMC5009925

[12] Hamamci, I. E. et al. A foundation model utilizing chest CT volumes and radiology reports for supervised-level zero-shot detection of abnormalities. Preprint at 10.48550/arXiv.2403.17834, https://www.researchsquare.com/article/rs-5271327/v1 (2024).

[13] Johnson, A., Pollard, T., Horng, S., Celi, L. A. & Mark, R. MIMIC-IV-Note: Deidentified free-text clinical notes (version 2.2). *PhysioNet*. 10.13026/1n74-ne17 (2023).

[14] Kefeli, J. TCGA-reports: a machine-readable pathology report resource for benchmarking text-based AI models. Kefeli et al. Mendeley Data 10.17632/HYG5XKZNPX.1 (2024). 10.1016/j.patter.2024.100933 PMC10935496 38487800

[15] Li, Y. et al. ChatDoctor: a medical chat model fine-tuned on a large language model meta-AI (LLaMA) using medical domain knowledge. *Cureus*10.7759/cureus.40895 (2023). 10.7759/cureus.40895 PMC10364849 37492832

[16] Wang, A., Liu, C., Yang, J. & Weng, C. Fine-tuning large language models for rare disease concept normalization. *J. Am. Med. Inform. Assoc.* **31**, 2076–2083 (2024). 38829731 10.1093/jamia/ocae133 PMC11339522

[17] Singhal, K. et al. Large language models encode clinical knowledge. *Nature* **620**, 172–180 (2023). 37438534 10.1038/s41586-023-06291-2 PMC10396962

[18] Jin, Q. et al. Matching patients to clinical trials with large language models. *Nat. Commun.* **15**, 9074 (2024). 39557832 10.1038/s41467-024-53081-z PMC11574183

[19] Lin, C.-Y. *ROUGE: a package for automatic evaluation of summaries*. Text summarization branches out, 74–81 (Association for Computational Linguistics, 2004).

[20] Lu, Q., Dou, D. & Nguyen, T. ClinicalT5: a generative language model for clinical text. In: *Findings of the association for computational linguistics: EMNLP, 2022*, 5436–5443 (Association for Computational Linguistics, 2022). 10.18653/v1/2022.findings-emnlp.398.

[21] Yan, A. et al. RadBERT: adapting transformer-based language models to radiology. *Radiol. Artif. Intell.* **4**, e210258 (2022). 35923376 10.1148/ryai.210258 PMC9344353

[22] Verkijk, S. & Vossen, P. MedRoBERTa.nl: A Language Model for Dutch Electronic Health Records. *Comput. Linguist. Neth. J.* **11**, 141–159. Retrieved from https://clinjournal.org/clinj/article/view/132 (2021).

[23] Huang, K., Altosaar, J. & Ranganath, R. ClinicalBERT: modeling clinical notes and predicting hospital readmission. Preprint at 10.48550/ARXIV.1904.05342 (2019).

[24] Busch, F. et al. Large language models for structured reporting in radiology: past, present, and future. *Eur. Radiol.*10.1007/s00330-024-11107-6 (2024). 10.1007/s00330-024-11107-6 PMC12021971 39438330

[25] Meakin, J. et al. Grand-Challenge.org. *Zenodo*10.5281/ZENODO.3356819 (2023).

[26] Deng, J. et al. ImageNet: A large-scale hierarchical image database. In *2009 IEEE Conference on Computer Vision and Pattern Recognition* 248–255 (IEEE, 2009). 10.1109/CVPR.2009.5206848.

[27] Wang, A. et al. GLUE: a multi-task benchmark and analysis platform for natural language understanding. International Conference on Learning Representations https://openreview.net/forum?id=rJ4km2R5t7 (2019).

[28] Buckeye, A. J. et al. Data Science Bowl 2017. https://kaggle.com/competitions/data-science-bowl-2017 (2017).

[29] Devlin, J., Chang, M.-W., Lee, K. & Toutanova, K. BERT: pre-training of deep bidirectional transformers for language understanding. Preprint at http://arxiv.org/abs/1810.04805 (2019).

[30] Central Intelligence Agency. *Field listing - languages* (Central Intelligence Agency, 2007).

[31] Gu, Y. et al. Domain-specific language model pretraining for biomedical natural language processing. *ACM Trans. Comput. Healthc.* **3**, 1–23 (2022).

[32] Rietberg, M. T., Nguyen, V. B., Geerdink, J., Vijlbrief, O. & Seifert, C. Accurate and reliable classification of unstructured reports on their diagnostic goal using BERT models. *Diagnostics* **13**, 1251 (2023). 37046469 10.3390/diagnostics13071251 PMC10093295

[33] Liu, Y. et al. RoBERTa: a robustly optimized BERT pretraining approach. Preprint at http://arxiv.org/abs/1907.11692 (2019).

[34] Beltagy, I., Peters, M. E. & Cohan, A. Longformer: the long-document transformer. Preprint at http://arxiv.org/abs/2004.05150 (2020).

[35] Gao, Y. et al. A scoping review of publicly available language tasks in clinical natural language processing. *J. Am. Med. Inform. Assoc.* **29**, 1797–1806 (2022). 35923088 10.1093/jamia/ocac127 PMC9471718

[36] Gao, Y. et al. DR.BENCH: diagnostic reasoning benchmark for clinical natural language processing. *J. Biomed. Inform.* **138**, 104286 (2023). 36706848 10.1016/j.jbi.2023.104286 PMC9993808

[37] Hendrix, W. et al. Trends in the incidence of pulmonary nodules in chest computed tomography: 10-year results from two Dutch hospitals. *Eur. Radiol.*10.1007/s00330-023-09826-3 (2023). 10.1007/s00330-023-09826-3 PMC10598118 37338552

[38] Carrell, D. et al. Hiding in plain sight: use of realistic surrogates to reduce exposure of protected health information in clinical text. *J. Am. Med. Inf. Assoc.* **20**, 342–348 (2013). 10.1136/amiajnl-2012-001034 PMC3638183 22771529

[39] Natural Language Processing and Computational Linguistics group at the University of Groningen. bert-base-dutch-cased. *Hugging Face*10.57967/HF/0149 (2022).

[40] Joeran Bosma. dragon-bert-base-mixed-domain. *Hugging Face*10.57967/HF/2166 (2024).

[41] Joeran Bosma. dragon-bert-base-domain-specific. *Hugging Face*10.57967/HF/2167 (2024).

[42] Joeran Bosma. dragon-roberta-base-mixed-domain. *Hugging Face*10.57967/HF/2168 (2024).

[43] Joeran Bosma. dragon-roberta-base-domain-specific. *Hugging Face*10.57967/HF/2169 (2024).

[44] Joeran Bosma. dragon-roberta-large-mixed-domain. *Hugging Face*10.57967/HF/2170 (2024).

[45] Joeran Bosma. dragon-roberta-large-domain-specific. *Hugging Face*10.57967/HF/2171 (2024).

[46] Joeran Bosma. dragon-longformer-base-mixed-domain. *Hugging Face*10.57967/HF/2172 (2024).

[47] Joeran Bosma. dragon-longformer-base-domain-specific. *Hugging Face*10.57967/HF/2173 (2024).

[48] Joeran Bosma. dragon-longformer-large-mixed-domain. *Hugging Face*10.57967/HF/2174 (2024).

[49] Joeran Bosma. dragon-longformer-large-domain-specific. *Hugging Face*10.57967/HF/2175 (2024).

[50] Bosma, J. S. et al. *DRAGON statistical analysis plan*. 10.5281/ZENODO.10374512 (2024).

