# Supplementary material for: The DRAGON benchmark for clinical NLP
Source: NPJ Digit Med. 2025 May 17;8:289. doi: 10.1038/s41746-025-01626-x (PMC12084576; doi:10.1038/s41746-025-01626-x)
Supplement: Supplementary file 1 — Supplementary Information [file 41746_2025_1626_MOESM1_ESM.pdf]

DRAGON Performance Benchmark for Clinical NLP Supplementary Information

Supplementary Note 1: Evidence Synthesis

Here the choice of the primary metric for each task type is motivated.

For binary classification tasks (e.g., predicting abnormality presence) the area under the receiver operating characteristic curve (AUROC) metric was used. This metric evaluates model performance across all possible operation points. Since different applications have different trade-offs for sensitivity and specificity, this evaluation results in a representative performance assessment for many different applications. For binary classification tasks, it is not predetermined whether identifying positive or negative cases is more critical, since this may depend on the specific downstream application. Therefore, we selected the class-neutral AUROC rather than the positive-class-focused AUPRC, consistent with established recommendations for metric selection[1]. For multi-label binary classification tasks that share the same objective across labels (e.g., predicting the presence of a lesion measurement for multiple lesions) the labels and predictions are pooled and a single AUROC is calculated. For multi-label binary classification tasks that do not share the same objective across labels (e.g., identifying the presence of different lesion characteristics) the AUROC is calculated per label and then averaged ("macro AUROC").

For ordinal multi-class classification tasks (e.g., predicting the number of aggressive cancer lesions), the linearly weighted Cohen's kappa metric was used. For non-ordinal multi-class classification tasks (e.g., predicting the sample origin) and multi-label binary classification tasks that do not share the same objective across labels (e.g., predicting multiple binary unrelated diagnoses), the unweighted kappa metric was used. The kappa metrics incorporate the class frequency in the evaluation to adjust for chance agreement, providing a more nuanced measure of classification accuracy that accounts for the imbalance in class distribution. The weighting for ordinal tasks represents that being further away from the true label is worse (e.g., predicting 0 for a case with 3 aggressive cancer lesions is worse than predicting 2). For our analysis, we selected a linearly weighted Cohen's kappa rather than, for example, a quadratically weighted version. This choice aligns with the clinical context of our tasks: deviations two categories apart should be considered roughly twice as consequential as those one category apart, rather than penalized according to a squared distance. While alternatives like Krippendorff's Alpha natively support ordinal data and offer greater flexibility for various rating conditions, we opted for linearly weighted Cohen's kappa due to its broad recognition, frequent use in similar studies, and consequently greater accessibility and interpretability for a wider audience[1].

For named entity recognition, the F1 metric was used. The F1 score harmonizes the precision and recall of a classifier into a single metric by taking their harmonic mean, offering a balance between the classifier's ability to correctly label positive instances (precision) and its capability to identify all positive instances (recall). For single-label named entity recognition, the macro F1 metric was used. For multi-label named entity recognition, the weighted F1 metric was used.

For regression tasks (e.g., predicting the lesion diameter measurement), the evaluation needs to focus on four key principles to ensure a balanced and clinically relevant assessment. First, the metric should be robust to outliers. Even though outliers in the predictions are undesirable, a single outlier should not dominate the evaluation. Second, the metric should treat small errors in small values with the same effect as larger errors in larger values. For instance, a 5 mm error in estimating a 25 mm lesion is similar in diagnostical impact compared to a 1 mm error in a 5 mm lesion. Third, the metric should be symmetrical, meaning that swapping the ground truth label and prediction should not alter the score. This recognizes that predicting 4 mm for a 6 mm lesion is similar in diagnostic impact compared to predicting 6 mm for a 4 mm lesion. Lastly, minor measurement errors (e.g., when measuring the lesion in a medical scan) should not disproportionately affect the overall score. For

example, when measuring lesions in a scan with 1 mm voxel spacing, a few voxels would change the measurement of very small lesions but not the diagnostic outcome. These requirements ensure that the primary metric remains a reliable indicator of clinically relevant performance, reflecting the nuanced nature of medical diagnostics and patient outcomes. Most typical regression metrics (e.g., mean squared error, mean absolute error, mean percentage error) do not satisfy these requirements. Therefore, the Robust Symmetric Mean Absolute Percentage Error Score (RSMAPES) metric was used:

$$1 - \frac{1}{N} \sum_{i}^{N} \frac{|y - \hat{y}|}{|\hat{y}| + |y| + \epsilon}$$

Where N is the number of cases, y is the target value, $\hat{y}$ is the predicted value, and ϵ is a positive value. We set ϵ to four times the estimated measurement error, which empirically resulted in good correspondence between errors and their diagnostic impact.

We define thresholds to describe the observed performance as "excellent", "good", etc. For the Kappa metrics, we follow the thresholds proposed by McHugh[2] for health related studies. For the AUROC metric, we use the thresholds proposed by Nahm[3] for clinicians and subdivide the 0.6 – 0.7 range of "poor" agreement into 0.6 – 0.65 "minimal/poor" and 0.65 – 0.7 "weak/poor" to align with the Kappa metrics. To the best of our knowledge, no literature exists that motivates interpretation ranges for the F1 and RSMAPES metrics. Therefore, we followed the same thresholds as for Kappa, since the typical range of values is [0, 1] for each metric, and based on our experience with these metrics, the interpretation fits to the F1 and RSMAPES metrics too.

Supplementary Table 1: interpretation of metric values. Ranges are including the left value and up to but excluding the right value, i.e. $[s_0, s_1)$.

Metric	None / Fail	Minimal / Poor	Weak / Poor	Moderate / Fair	Strong / Good	Almost Perfect / Excellent
Kappa	0.00 – 0.21	0.21 – 0.40	0.40 – 0.60	0.60 – 0.80	0.80 – 0.90	0.90 – 1.0
AUROC	0.50 – 0.60	0.60 – 0.65	0.65 – 0.70	0.70 – 0.80	0.80 – 0.90	0.90 – 1.0
F1	0.20 – 0.21	0.21 – 0.40	0.40 – 0.60	0.60 – 0.80	0.80 – 0.90	0.90 – 1.0
RSMAPES	0.00 – 0.21	0.21 – 0.40	0.40 – 0.60	0.60 – 0.80	0.80 – 0.90	0.90 – 1.0

References

1. Maier-Hein, L. *et al.* Metrics reloaded: recommendations for image analysis validation. *Nat Methods* **21**, 195–212 (2024).
2. McHugh, M. L. Interrater reliability: the kappa statistic. *Biochem Med* 276–282 (2012) doi:10.11613/BM.2012.031.
3. Nahm, F. S. Receiver operating characteristic curve: overview and practical use for clinicians. *Korean J Anesthesiol* **75**, 25–36 (2022).

Supplementary Note 2: Test Performance Across Tasks

Supplementary Table 2: Test performance across tasks. For each task and architecture, the mean ± standard deviation across the five fine-tuning runs (with different partitions of the development data) is shown. AUROC = area under the receiver operating characteristic curve, Kappa = Cohen's Kappa. RSMAPES = robust symmetric mean absolute percentage error score.

ID	Name	Pretraining strategy	BERT base	Longformer base	Longformer large	RoBERTa base	RoBERTa large	Metric
T1	Adhesion presence	Domain	0.978 ± 0.013	0.986 ± 0.003	0.979 ± 0.006	0.991 ± 0.001	0.983 ± 0.005	AUROC
T1	Adhesion presence	General	0.998 ± 0.002	0.902 ± 0.045	0.952 ± 0.034	0.985 ± 0.001	0.987 ± 0.006	AUROC
T1	Adhesion presence	Mixed	0.993 ± 0.001	0.978 ± 0.029	0.985 ± 0.017	0.985 ± 0.006	0.992 ± 0.003	AUROC
T2	Pulmonary nodule presence	Domain	0.954 ± 0.008	0.945 ± 0.004	0.936 ± 0.016	0.953 ± 0.005	0.958 ± 0.009	AUROC
T2	Pulmonary nodule presence	General	0.820 ± 0.041	0.756 ± 0.066	0.716 ± 0.097	0.855 ± 0.034	0.923 ± 0.015	AUROC
T2	Pulmonary nodule presence	Mixed	0.934 ± 0.014	0.900 ± 0.009	0.953 ± 0.010	0.949 ± 0.008	0.948 ± 0.009	AUROC
T3	Kidney abnormality identification	Domain	0.751 ± 0.016	0.762 ± 0.003	0.920 ± 0.031	0.782 ± 0.017	0.842 ± 0.107	AUROC
T3	Kidney abnormality identification	General	0.762 ± 0.052	0.565 ± 0.101	0.579 ± 0.057	0.741 ± 0.027	0.843 ± 0.045	AUROC
T3	Kidney abnormality identification	Mixed	0.796 ± 0.006	0.546 ± 0.037	0.571 ± 0.059	0.623 ± 0.028	0.664 ± 0.025	AUROC
T4	Skin histopathology case selection	Domain	0.998 ± 0.001	0.996 ± 0.000	0.996 ± 0.001	0.999 ± 0.001	0.996 ± 0.001	AUROC
T4	Skin histopathology case selection	General	0.998 ± 0.001	0.998 ± 0.001	0.996 ± 0.001	0.981 ± 0.020	0.996 ± 0.001	AUROC
T4	Skin histopathology case selection	Mixed	0.996 ± 0.001	0.996 ± 0.002	0.996 ± 0.001	0.997 ± 0.001	0.997 ± 0.002	AUROC
T5	RECIST timeline	Domain	0.962 ± 0.008	0.964 ± 0.003	0.964 ± 0.002	0.964 ± 0.003	0.944 ± 0.011	AUROC
T5	RECIST timeline	General	0.954 ± 0.006	0.942 ± 0.006	0.886 ± 0.036	0.952 ± 0.006	0.961 ± 0.015	AUROC
T5	RECIST timeline	Mixed	0.972 ± 0.004	0.935 ± 0.004	0.917 ± 0.014	0.931 ± 0.020	0.943 ± 0.019	AUROC
T6	Histopathology cancer origin	Domain	0.700 ± 0.054	0.743 ± 0.014	0.718 ± 0.039	0.716 ± 0.037	0.631 ± 0.047	AUROC
T6	Histopathology cancer origin	General	0.692 ± 0.037	0.514 ± 0.063	0.592 ± 0.066	0.552 ± 0.128	0.610 ± 0.118	AUROC
T6	Histopathology cancer origin	Mixed	0.696 ± 0.090	0.544 ± 0.084	0.509 ± 0.078	0.529 ± 0.057	0.576 ± 0.030	AUROC
T7	Pulmonary nodule size presence	Domain	0.667 ± 0.017	0.715 ± 0.021	0.852 ± 0.013	0.803 ± 0.021	0.870 ± 0.045	AUROC
T7	Pulmonary nodule size presence	General	0.761 ± 0.058	0.708 ± 0.153	0.570 ± 0.070	0.781 ± 0.040	0.652 ± 0.146	AUROC
T7	Pulmonary nodule size presence	Mixed	0.652 ± 0.070	0.562 ± 0.067	0.632 ± 0.121	0.663 ± 0.078	0.652 ± 0.059	AUROC
T8	PDAC size presence	Domain	0.652 ± 0.037	0.715 ± 0.012	0.777 ± 0.007	0.743 ± 0.013	0.640 ± 0.056	AUROC
T8	PDAC size presence	General	0.688 ± 0.026	0.614 ± 0.082	0.628 ± 0.056	0.586 ± 0.125	0.655 ± 0.068	AUROC
T8	PDAC size presence	Mixed	0.616 ± 0.073	0.612 ± 0.029	0.609 ± 0.060	0.573 ± 0.040	0.534 ± 0.036	AUROC
T9	PDAC diagnosis	Domain	0.749 ± 0.042	0.680 ± 0.024	0.709 ± 0.036	0.743 ± 0.005	0.767 ± 0.044	Unweighted Kappa
T9	PDAC diagnosis	General	0.690 ± 0.018	0.652 ± 0.016	0.623 ± 0.050	0.675 ± 0.051	0.728 ± 0.014	Unweighted Kappa
T9	PDAC diagnosis	Mixed	0.726 ± 0.036	0.697 ± 0.104	0.773 ± 0.025	0.747 ± 0.036	0.779 ± 0.032	Unweighted Kappa
T10	Prostate radiology suspicious lesions	Domain	0.975 ± 0.004	0.967 ± 0.002	0.952 ± 0.004	0.975 ± 0.003	0.975 ± 0.003	Linearly Weighted Kappa
T10	Prostate radiology suspicious lesions	General	0.960 ± 0.003	0.961 ± 0.009	0.963 ± 0.009	0.967 ± 0.007	0.980 ± 0.001	Linearly Weighted Kappa
T10	Prostate radiology suspicious lesions	Mixed	0.969 ± 0.005	0.974 ± 0.007	0.981 ± 0.004	0.972 ± 0.004	0.982 ± 0.002	Linearly Weighted Kappa
T11	Prostate histopathology significant cancers	Domain	0.863 ± 0.009	0.851 ± 0.006	0.805 ± 0.009	0.868 ± 0.002	0.861 ± 0.004	Linearly Weighted Kappa
T11	Prostate histopathology significant cancers	General	0.835 ± 0.013	0.847 ± 0.023	0.842 ± 0.028	0.834 ± 0.010	0.879 ± 0.007	Linearly Weighted Kappa
T11	Prostate histopathology significant cancers	Mixed	0.875 ± 0.013	0.853 ± 0.008	0.833 ± 0.005	0.866 ± 0.004	0.858 ± 0.022	Linearly Weighted Kappa
T12	Histopathology tissue type	Domain	0.331 ± 0.039	0.344 ± 0.033	0.505 ± 0.028	0.348 ± 0.005	0.428 ± 0.063	Unweighted Kappa

T12	Histopathology tissue type	General	0.380 ± 0.024	0.277 ± 0.183	0.436 ± 0.218	0.238 ± 0.156	0.322 ± 0.140	Unweighted Kappa
T12	Histopathology tissue type	Mixed	0.361 ± 0.120	0.391 ± 0.099	0.738 ± 0.052	0.153 ± 0.044	0.312 ± 0.090	Unweighted Kappa
T13	Histopathology tissue origin	Domain	0.212 ± 0.145	0.000 ± 0.000	0.426 ± 0.057	0.000 ± 0.000	0.669 ± 0.025	Unweighted Kappa
T13	Histopathology tissue origin	General	0.352 ± 0.121	0.131 ± 0.153	0.393 ± 0.068	0.000 ± 0.000	0.270 ± 0.167	Unweighted Kappa
T13	Histopathology tissue origin	Mixed	0.596 ± 0.137	0.187 ± 0.246	0.508 ± 0.193	0.140 ± 0.206	0.674 ± 0.078	Unweighted Kappa
T14	Entailment diagnostic sentences	Domain	0.599 ± 0.018	0.583 ± 0.011	0.552 ± 0.024	0.593 ± 0.007	0.577 ± 0.010	Linearly Weighted Kappa
T14	Entailment diagnostic sentences	General	0.592 ± 0.028	0.565 ± 0.012	0.467 ± 0.262	0.651 ± 0.013	0.406 ± 0.372	Linearly Weighted Kappa
T14	Entailment diagnostic sentences	Mixed	0.595 ± 0.027	0.647 ± 0.010	0.538 ± 0.301	0.660 ± 0.011	0.712 ± 0.019	Linearly Weighted Kappa
T15	Colon histopathology diagnosis	Domain	0.943 ± 0.005	0.930 ± 0.025	0.962 ± 0.004	0.954 ± 0.007	0.991 ± 0.011	Macro AUROC
T15	Colon histopathology diagnosis	General	0.956 ± 0.013	0.979 ± 0.006	0.992 ± 0.003	0.916 ± 0.022	0.990 ± 0.004	Macro AUROC
T15	Colon histopathology diagnosis	Mixed	0.977 ± 0.001	0.983 ± 0.006	0.995 ± 0.001	0.967 ± 0.026	0.993 ± 0.004	Macro AUROC
T16	RECIST lesion size presence	Domain	0.863 ± 0.003	0.870 ± 0.003	0.923 ± 0.008	0.879 ± 0.006	0.903 ± 0.016	AUROC
T16	RECIST lesion size presence	General	0.857 ± 0.002	0.850 ± 0.002	0.854 ± 0.011	0.861 ± 0.005	0.858 ± 0.007	AUROC
T16	RECIST lesion size presence	Mixed	0.868 ± 0.008	0.849 ± 0.004	0.839 ± 0.004	0.848 ± 0.004	0.848 ± 0.010	AUROC
T17	PDAC attributes	Domain	0.563 ± 0.009	0.560 ± 0.000	0.583 ± 0.014	0.566 ± 0.009	0.639 ± 0.083	Unweighted Kappa
T17	PDAC attributes	General	0.587 ± 0.005	0.560 ± 0.000	0.555 ± 0.018	0.560 ± 0.000	0.560 ± 0.000	Unweighted Kappa
T17	PDAC attributes	Mixed	0.568 ± 0.007	0.567 ± 0.009	0.554 ± 0.023	0.563 ± 0.004	0.564 ± 0.018	Unweighted Kappa
T18	Hip Kellgren-Lawrence scoring	Domain	0.683 ± 0.015	0.599 ± 0.047	0.566 ± 0.029	0.701 ± 0.008	0.686 ± 0.016	Unweighted Kappa
T18	Hip Kellgren-Lawrence scoring	General	0.626 ± 0.015	0.625 ± 0.018	0.618 ± 0.023	0.510 ± 0.096	0.641 ± 0.024	Unweighted Kappa
T18	Hip Kellgren-Lawrence scoring	Mixed	0.673 ± 0.011	0.664 ± 0.020	0.681 ± 0.019	0.678 ± 0.006	0.692 ± 0.018	Unweighted Kappa
T19	Prostate volume measurement	Domain	0.982 ± 0.001	0.976 ± 0.002	0.975 ± 0.002	0.978 ± 0.002	0.981 ± 0.002	RSMAPES (ε = 4 cm3)
T19	Prostate volume measurement	General	0.980 ± 0.002	0.980 ± 0.003	0.983 ± 0.003	0.970 ± 0.010	0.979 ± 0.005	RSMAPES (ε = 4 cm3)
T19	Prostate volume measurement	Mixed	0.981 ± 0.001	0.983 ± 0.002	0.986 ± 0.002	0.979 ± 0.003	0.984 ± 0.001	RSMAPES (ε = 4 cm3)
T20	Prostate specific antigen measurement	Domain	0.972 ± 0.002	0.961 ± 0.009	0.961 ± 0.010	0.968 ± 0.003	0.974 ± 0.002	RSMAPES (ε = 0.4 ng/mL)
T20	Prostate specific antigen measurement	General	0.973 ± 0.002	0.975 ± 0.002	0.975 ± 0.005	0.924 ± 0.084	0.965 ± 0.003	RSMAPES (ε = 0.4 ng/mL)
T20	Prostate specific antigen measurement	Mixed	0.975 ± 0.002	0.977 ± 0.002	0.971 ± 0.007	0.967 ± 0.003	0.975 ± 0.004	RSMAPES (ε = 0.4 ng/mL)
T21	Prostate specific antigen density measurement	Domain	0.956 ± 0.006	0.901 ± 0.086	0.940 ± 0.013	0.944 ± 0.009	0.955 ± 0.013	RSMAPES (ε = 0.04 ng/mL2)
T21	Prostate specific antigen density measurement	General	0.954 ± 0.004	0.964 ± 0.003	0.959 ± 0.008	0.929 ± 0.022	0.935 ± 0.018	RSMAPES (ε = 0.04 ng/mL2)
T21	Prostate specific antigen density measurement	Mixed	0.958 ± 0.010	0.961 ± 0.005	0.972 ± 0.009	0.958 ± 0.007	0.961 ± 0.009	RSMAPES (ε = 0.04 ng/mL2)
T22	PDAC size measurement	Domain	0.851 ± 0.002	0.849 ± 0.003	0.849 ± 0.004	0.854 ± 0.001	0.854 ± 0.003	RSMAPES (ε = 4 mm)
T22	PDAC size measurement	General	0.840 ± 0.003	0.844 ± 0.002	0.843 ± 0.007	0.846 ± 0.001	0.848 ± 0.002	RSMAPES (ε = 4 mm)
T22	PDAC size measurement	Mixed	0.841 ± 0.005	0.839 ± 0.006	0.834 ± 0.006	0.845 ± 0.002	0.845 ± 0.005	RSMAPES (ε = 4 mm)
T23	Pulmonary nodule size measurement	Domain	0.790 ± 0.011	0.772 ± 0.004	0.796 ± 0.017	0.800 ± 0.004	0.818 ± 0.014	RSMAPES (ε = 4 mm)
T23	Pulmonary nodule size measurement	General	0.779 ± 0.012	0.761 ± 0.005	0.761 ± 0.014	0.760 ± 0.004	0.764 ± 0.006	RSMAPES (ε = 4 mm)
T23	Pulmonary nodule size measurement	Mixed	0.791 ± 0.021	0.771 ± 0.007	0.783 ± 0.019	0.781 ± 0.003	0.792 ± 0.018	RSMAPES (ε = 4 mm)

T24	RECIST lesion size measurements	Domain	0.781 ± 0.003	0.775 ± 0.002	0.783 ± 0.004	0.787 ± 0.003	0.783 ± 0.003	RSMAPES (ε = 4 mm)
T24	RECIST lesion size measurements	General	0.776 ± 0.005	0.770 ± 0.004	0.769 ± 0.006	0.767 ± 0.006	0.769 ± 0.006	RSMAPES (ε = 4 mm)
T24	RECIST lesion size measurements	Mixed	0.776 ± 0.003	0.776 ± 0.004	0.770 ± 0.003	0.774 ± 0.003	0.777 ± 0.003	RSMAPES (ε = 4 mm)
T25	Anonymization	Domain	0.541 ± 0.028	0.503 ± 0.011	0.601 ± 0.032	0.554 ± 0.023	0.816 ± 0.008	Macro F1
T25	Anonymization	General	0.676 ± 0.019	0.654 ± 0.012	0.000 ± 0.000	0.680 ± 0.007	0.874 ± 0.024	Macro F1
T25	Anonymization	Mixed	0.709 ± 0.022	0.662 ± 0.019	0.000 ± 0.000	0.714 ± 0.043	0.897 ± 0.009	Macro F1
T26	Medical terminology recognition	Domain	0.673 ± 0.005	0.629 ± 0.027	0.807 ± 0.003	0.679 ± 0.007	0.835 ± 0.004	F1
T26	Medical terminology recognition	General	0.721 ± 0.011	0.695 ± 0.003	0.632 ± 0.084	0.731 ± 0.007	0.812 ± 0.023	F1
T26	Medical terminology recognition	Mixed	0.742 ± 0.008	0.722 ± 0.004	0.729 ± 0.063	0.726 ± 0.002	0.799 ± 0.005	F1
T27	Prostate biopsy sampling	Domain	0.723 ± 0.075	0.325 ± 0.030	0.627 ± 0.022	0.847 ± 0.027	0.898 ± 0.011	Weighted F1
T27	Prostate biopsy sampling	General	0.904 ± 0.009	0.908 ± 0.010	0.003 ± 0.006	0.894 ± 0.023	0.901 ± 0.016	Weighted F1
T27	Prostate biopsy sampling	Mixed	0.906 ± 0.008	0.916 ± 0.009	0.008 ± 0.011	0.917 ± 0.003	0.927 ± 0.009	Weighted F1
T28	Skin histopathology diagnosis	Domain	0.483 ± 0.007	0.352 ± 0.138	0.497 ± 0.009	0.506 ± 0.032	0.666 ± 0.040	Weighted F1
T28	Skin histopathology diagnosis	General	0.514 ± 0.011	0.344 ± 0.214	0.000 ± 0.001	0.454 ± 0.098	0.525 ± 0.015	Weighted F1
T28	Skin histopathology diagnosis	Mixed	0.401 ± 0.220	0.409 ± 0.223	0.002 ± 0.003	0.511 ± 0.017	0.563 ± 0.036	Weighted F1

Supplementary Note 3: Additional Performance Metrics

Supplementary Table 3: Additional performance metrics across tasks. For each task and architecture, the mean ± standard deviation across the five fine-tuning runs (with different partitions of the development data) is shown. Correlation = Pearson correlation. Ratio within = Ratio of cases with a prediction within the specified error margin.

ID	Name	Pretraining strategy	BERT base	Longformer base	Longformer large	RoBERTa base	RoBERTa large	Metric
T1	Adhesion presence	Domain	0.947 ± 0.012	0.949 ± 0.012	0.936 ± 0.031	0.975 ± 0.002	0.977 ± 0.006	Accuracy
T1	Adhesion presence	General	0.977 ± 0.010	0.861 ± 0.042	0.913 ± 0.033	0.870 ± 0.086	0.971 ± 0.012	Accuracy
T1	Adhesion presence	Mixed	0.977 ± 0.007	0.942 ± 0.045	0.953 ± 0.027	0.951 ± 0.033	0.983 ± 0.005	Accuracy
T2	Pulmonary nodule presence	Domain	0.897 ± 0.010	0.880 ± 0.007	0.872 ± 0.020	0.902 ± 0.006	0.909 ± 0.010	Accuracy
T2	Pulmonary nodule presence	General	0.783 ± 0.026	0.709 ± 0.052	0.704 ± 0.080	0.819 ± 0.036	0.874 ± 0.023	Accuracy
T2	Pulmonary nodule presence	Mixed	0.866 ± 0.016	0.839 ± 0.015	0.891 ± 0.005	0.893 ± 0.015	0.897 ± 0.012	Accuracy
T2	Pulmonary nodule presence	Domain	0.861 ± 0.011	0.827 ± 0.007	0.770 ± 0.034	0.873 ± 0.015	0.879 ± 0.037	Sensitivity
T2	Pulmonary nodule presence	General	0.591 ± 0.090	0.418 ± 0.187	0.306 ± 0.250	0.667 ± 0.106	0.788 ± 0.089	Sensitivity
T2	Pulmonary nodule presence	Mixed	0.797 ± 0.062	0.767 ± 0.071	0.852 ± 0.069	0.903 ± 0.015	0.827 ± 0.046	Sensitivity
T2	Pulmonary nodule presence	Domain	0.915 ± 0.010	0.906 ± 0.011	0.922 ± 0.032	0.916 ± 0.003	0.924 ± 0.015	Specificity
T2	Pulmonary nodule presence	General	0.878 ± 0.020	0.852 ± 0.076	0.900 ± 0.095	0.894 ± 0.013	0.916 ± 0.042	Specificity
T2	Pulmonary nodule presence	Mixed	0.900 ± 0.015	0.875 ± 0.039	0.910 ± 0.031	0.888 ± 0.019	0.931 ± 0.016	Specificity
T3	Kidney abnormality identification	Domain	0.690 ± 0.017	0.664 ± 0.007	0.855 ± 0.034	0.697 ± 0.011	0.783 ± 0.103	Accuracy
T3	Kidney abnormality identification	General	0.666 ± 0.051	0.567 ± 0.040	0.540 ± 0.043	0.666 ± 0.032	0.775 ± 0.033	Accuracy
T3	Kidney abnormality identification	Mixed	0.707 ± 0.019	0.530 ± 0.026	0.554 ± 0.049	0.600 ± 0.029	0.621 ± 0.021	Accuracy
T4	Skin histopathology case selection	Domain	0.992 ± 0.005	0.996 ± 0.000	0.996 ± 0.000	0.996 ± 0.000	0.995 ± 0.002	Accuracy
T4	Skin histopathology case selection	General	0.993 ± 0.005	0.994 ± 0.004	0.993 ± 0.004	0.956 ± 0.009	0.996 ± 0.000	Accuracy
T4	Skin histopathology case selection	Mixed	0.991 ± 0.004	0.995 ± 0.002	0.991 ± 0.006	0.994 ± 0.004	0.996 ± 0.000	Accuracy
T5	RECIST timeline	Domain	0.928 ± 0.004	0.926 ± 0.003	0.936 ± 0.007	0.924 ± 0.005	0.921 ± 0.011	Accuracy
T5	RECIST timeline	General	0.924 ± 0.005	0.911 ± 0.017	0.825 ± 0.071	0.701 ± 0.118	0.909 ± 0.023	Accuracy
T5	RECIST timeline	Mixed	0.936 ± 0.016	0.919 ± 0.010	0.847 ± 0.031	0.929 ± 0.004	0.923 ± 0.003	Accuracy
T6	Histopathology cancer origin	Domain	0.891 ± 0.000	0.891 ± 0.000	0.891 ± 0.000	0.891 ± 0.000	0.890 ± 0.003	Accuracy
T6	Histopathology cancer origin	General	0.891 ± 0.000	0.891 ± 0.000	0.891 ± 0.000	0.891 ± 0.000	0.891 ± 0.000	Accuracy
T6	Histopathology cancer origin	Mixed	0.891 ± 0.000	0.891 ± 0.000	0.891 ± 0.001	0.891 ± 0.000	0.891 ± 0.000	Accuracy
T7	Pulmonary nodule size presence	Domain	0.630 ± 0.046	0.664 ± 0.032	0.785 ± 0.035	0.733 ± 0.037	0.812 ± 0.041	Accuracy
T7	Pulmonary nodule size presence	General	0.718 ± 0.083	0.618 ± 0.144	0.530 ± 0.045	0.670 ± 0.100	0.555 ± 0.092	Accuracy
T7	Pulmonary nodule size presence	Mixed	0.612 ± 0.049	0.542 ± 0.076	0.548 ± 0.073	0.609 ± 0.078	0.618 ± 0.058	Accuracy
T7	Pulmonary nodule size presence	Domain	0.619 ± 0.102	0.762 ± 0.054	0.850 ± 0.054	0.775 ± 0.036	0.838 ± 0.050	Sensitivity
T7	Pulmonary nodule size presence	General	0.769 ± 0.098	0.769 ± 0.173	0.800 ± 0.177	0.894 ± 0.096	0.894 ± 0.139	Sensitivity
T7	Pulmonary nodule size presence	Mixed	0.681 ± 0.098	0.606 ± 0.108	0.600 ± 0.329	0.637 ± 0.129	0.625 ± 0.103	Sensitivity
T7	Pulmonary nodule size presence	Domain	0.641 ± 0.063	0.571 ± 0.071	0.724 ± 0.040	0.694 ± 0.069	0.788 ± 0.039	Specificity
T7	Pulmonary nodule size presence	General	0.671 ± 0.080	0.476 ± 0.128	0.276 ± 0.244	0.459 ± 0.256	0.235 ± 0.309	Specificity
T7	Pulmonary nodule size presence	Mixed	0.547 ± 0.030	0.482 ± 0.130	0.500 ± 0.284	0.582 ± 0.043	0.612 ± 0.078	Specificity
T8	PDAC size presence	Domain	0.838 ± 0.000	0.838 ± 0.000	0.868 ± 0.016	0.839 ± 0.002	0.828 ± 0.011	Accuracy
T8	PDAC size presence	General	0.839 ± 0.002	0.838 ± 0.000	0.845 ± 0.013	0.838 ± 0.000	0.838 ± 0.000	Accuracy
T8	PDAC size presence	Mixed	0.836 ± 0.003	0.838 ± 0.000	0.837 ± 0.002	0.838 ± 0.000	0.838 ± 0.000	Accuracy

T10	Prostate radiology suspicious lesions	Domain	0.983 ± 0.003	0.977 ± 0.001	0.966 ± 0.003	0.982 ± 0.002	0.983 ± 0.002	Accuracy
T10	Prostate radiology suspicious lesions	General	0.972 ± 0.002	0.973 ± 0.005	0.975 ± 0.005	0.977 ± 0.004	0.986 ± 0.001	Accuracy
T10	Prostate radiology suspicious lesions	Mixed	0.978 ± 0.003	0.982 ± 0.004	0.987 ± 0.002	0.981 ± 0.003	0.987 ± 0.002	Accuracy
T11	Prostate histopathology significant cancers	Domain	0.921 ± 0.004	0.914 ± 0.003	0.879 ± 0.008	0.922 ± 0.004	0.918 ± 0.005	Accuracy
T11	Prostate histopathology significant cancers	General	0.897 ± 0.012	0.917 ± 0.006	0.915 ± 0.009	0.912 ± 0.004	0.928 ± 0.003	Accuracy
T11	Prostate histopathology significant cancers	Mixed	0.924 ± 0.008	0.919 ± 0.002	0.912 ± 0.003	0.920 ± 0.004	0.922 ± 0.004	Accuracy
T19	Prostate volume measurement	Domain	0.950 ± 0.004	0.929 ± 0.006	0.878 ± 0.011	0.953 ± 0.006	0.950 ± 0.010	Correlation
T19	Prostate volume measurement	General	0.957 ± 0.003	0.955 ± 0.008	0.957 ± 0.004	0.935 ± 0.022	0.966 ± 0.005	Correlation
T19	Prostate volume measurement	Mixed	0.962 ± 0.002	0.965 ± 0.002	0.957 ± 0.005	0.953 ± 0.003	0.969 ± 0.008	Correlation
T19	Prostate volume measurement	Domain	0.409 ± 0.052	0.321 ± 0.041	0.357 ± 0.064	0.307 ± 0.035	0.379 ± 0.070	Ratio within 1 cm^3
T19	Prostate volume measurement	General	0.362 ± 0.052	0.389 ± 0.058	0.421 ± 0.162	0.279 ± 0.103	0.284 ± 0.131	Ratio within 1 cm^3
T19	Prostate volume measurement	Mixed	0.406 ± 0.074	0.440 ± 0.064	0.519 ± 0.036	0.376 ± 0.070	0.450 ± 0.051	Ratio within 1 cm^3
T19	Prostate volume measurement	Domain	0.906 ± 0.010	0.833 ± 0.020	0.856 ± 0.027	0.865 ± 0.029	0.882 ± 0.017	Ratio within 5 cm^3
T19	Prostate volume measurement	General	0.866 ± 0.020	0.866 ± 0.020	0.892 ± 0.053	0.766 ± 0.075	0.858 ± 0.071	Ratio within 5 cm^3
T19	Prostate volume measurement	Mixed	0.872 ± 0.015	0.874 ± 0.011	0.913 ± 0.038	0.834 ± 0.034	0.883 ± 0.019	Ratio within 5 cm^3
T20	Prostate specific antigen measurement	Domain	0.492 ± 0.016	0.462 ± 0.033	0.508 ± 0.006	0.495 ± 0.018	0.538 ± 0.005	Correlation
T20	Prostate specific antigen measurement	General	0.607 ± 0.041	0.578 ± 0.024	0.625 ± 0.081	0.468 ± 0.231	0.718 ± 0.017	Correlation
T20	Prostate specific antigen measurement	Mixed	0.609 ± 0.065	0.601 ± 0.046	0.623 ± 0.104	0.582 ± 0.024	0.770 ± 0.028	Correlation
T20	Prostate specific antigen measurement	Domain	0.895 ± 0.009	0.854 ± 0.036	0.855 ± 0.038	0.869 ± 0.030	0.895 ± 0.020	Ratio within 1 ng/mL
T20	Prostate specific antigen measurement	General	0.909 ± 0.013	0.899 ± 0.006	0.929 ± 0.008	0.698 ± 0.262	0.848 ± 0.044	Ratio within 1 ng/mL
T20	Prostate specific antigen measurement	Mixed	0.920 ± 0.005	0.916 ± 0.013	0.917 ± 0.021	0.863 ± 0.033	0.903 ± 0.013	Ratio within 1 ng/mL
T20	Prostate specific antigen measurement	Domain	0.975 ± 0.001	0.965 ± 0.009	0.961 ± 0.009	0.974 ± 0.002	0.976 ± 0.005	Ratio within 5 ng/mL
T20	Prostate specific antigen measurement	General	0.976 ± 0.002	0.974 ± 0.002	0.980 ± 0.003	0.921 ± 0.087	0.978 ± 0.001	Ratio within 5 ng/mL
T20	Prostate specific antigen measurement	Mixed	0.979 ± 0.002	0.979 ± 0.002	0.978 ± 0.007	0.967 ± 0.004	0.977 ± 0.005	Ratio within 5 ng/mL
T21	Prostate specific antigen density measurement	Domain	0.224 ± 0.027	0.165 ± 0.073	0.184 ± 0.026	0.238 ± 0.017	0.221 ± 0.008	Correlation
T21	Prostate specific antigen density measurement	General	0.193 ± 0.014	0.211 ± 0.032	0.271 ± 0.034	0.263 ± 0.033	0.297 ± 0.014	Correlation
T21	Prostate specific antigen density measurement	Mixed	0.260 ± 0.024	0.214 ± 0.015	0.209 ± 0.004	0.246 ± 0.036	0.218 ± 0.007	Correlation
T21	Prostate specific antigen density measurement	Domain	0.672 ± 0.064	0.382 ± 0.192	0.560 ± 0.090	0.495 ± 0.115	0.636 ± 0.119	Ratio within 0.01 ng/mL^2
T21	Prostate specific antigen density measurement	General	0.644 ± 0.074	0.815 ± 0.026	0.682 ± 0.165	0.404 ± 0.184	0.364 ± 0.163	Ratio within 0.01 ng/mL^2
T21	Prostate specific antigen density measurement	Mixed	0.664 ± 0.115	0.779 ± 0.037	0.852 ± 0.096	0.674 ± 0.099	0.691 ± 0.077	Ratio within 0.01 ng/mL^2
T21	Prostate specific antigen density measurement	Domain	0.956 ± 0.011	0.819 ± 0.249	0.915 ± 0.018	0.960 ± 0.002	0.960 ± 0.015	Ratio within 0.05 ng/mL^2
T21	Prostate specific antigen density measurement	General	0.963 ± 0.002	0.964 ± 0.005	0.970 ± 0.007	0.956 ± 0.004	0.957 ± 0.008	Ratio within 0.05 ng/mL^2
T21	Prostate specific antigen density measurement	Mixed	0.966 ± 0.001	0.966 ± 0.005	0.976 ± 0.001	0.959 ± 0.006	0.965 ± 0.009	Ratio within 0.05 ng/mL^2
T22	PDAC size measurement	Domain	0.073 ± 0.058	0.111 ± 0.020	0.243 ± 0.062	0.156 ± 0.026	0.327 ± 0.100	Correlation
T22	PDAC size measurement	General	-0.048 ± 0.036	0.006 ± 0.092	0.074 ± 0.068	0.043 ± 0.027	0.064 ± 0.025	Correlation
T22	PDAC size measurement	Mixed	-0.005 ± 0.080	-0.023 ± 0.042	-0.003 ± 0.023	0.000 ± 0.028	0.075 ± 0.093	Correlation
T22	PDAC size measurement	Domain	0.090 ± 0.020	0.067 ± 0.011	0.059 ± 0.010	0.097 ± 0.026	0.078 ± 0.024	Ratio within 1 mm
T22	PDAC size measurement	General	0.076 ± 0.021	0.090 ± 0.019	0.069 ± 0.037	0.079 ± 0.011	0.083 ± 0.024	Ratio within 1 mm

T22	PDAC size measurement	Mixed	0.056 ± 0.012	0.063 ± 0.019	0.071 ± 0.021	0.063 ± 0.011	0.078 ± 0.020	Ratio within 1 mm
T22	PDAC size measurement	Domain	0.400 ± 0.023	0.350 ± 0.005	0.316 ± 0.022	0.363 ± 0.018	0.328 ± 0.024	Ratio within 5 mm
T22	PDAC size measurement	General	0.320 ± 0.006	0.346 ± 0.023	0.325 ± 0.011	0.332 ± 0.008	0.341 ± 0.019	Ratio within 5 mm
T22	PDAC size measurement	Mixed	0.298 ± 0.033	0.324 ± 0.026	0.331 ± 0.027	0.327 ± 0.024	0.348 ± 0.030	Ratio within 5 mm
T23	Pulmonary nodule size measurement	Domain	0.562 ± 0.078	0.547 ± 0.087	0.564 ± 0.144	0.702 ± 0.043	0.581 ± 0.030	Correlation
T23	Pulmonary nodule size measurement	General	0.371 ± 0.058	0.349 ± 0.318	0.122 ± 0.277	0.352 ± 0.204	0.345 ± 0.179	Correlation
T23	Pulmonary nodule size measurement	Mixed	0.547 ± 0.197	0.196 ± 0.180	0.368 ± 0.249	0.356 ± 0.061	0.402 ± 0.120	Correlation
T23	Pulmonary nodule size measurement	Domain	0.069 ± 0.075	0.050 ± 0.051	0.119 ± 0.054	0.062 ± 0.056	0.181 ± 0.067	Ratio within 1 mm
T23	Pulmonary nodule size measurement	General	0.100 ± 0.036	0.069 ± 0.041	0.062 ± 0.034	0.100 ± 0.031	0.100 ± 0.031	Ratio within 1 mm
T23	Pulmonary nodule size measurement	Mixed	0.138 ± 0.083	0.094 ± 0.044	0.119 ± 0.064	0.094 ± 0.040	0.119 ± 0.072	Ratio within 1 mm
T23	Pulmonary nodule size measurement	Domain	0.537 ± 0.061	0.506 ± 0.067	0.512 ± 0.042	0.556 ± 0.023	0.613 ± 0.042	Ratio within 5 mm
T23	Pulmonary nodule size measurement	General	0.487 ± 0.081	0.463 ± 0.109	0.431 ± 0.089	0.494 ± 0.098	0.487 ± 0.078	Ratio within 5 mm
T23	Pulmonary nodule size measurement	Mixed	0.537 ± 0.023	0.512 ± 0.025	0.519 ± 0.058	0.525 ± 0.036	0.569 ± 0.075	Ratio within 5 mm
T24	RECIST lesion size measurements	Domain	0.323 ± 0.014	0.263 ± 0.022	0.344 ± 0.021	0.359 ± 0.017	0.308 ± 0.026	Correlation
T24	RECIST lesion size measurements	General	0.282 ± 0.037	0.174 ± 0.043	0.188 ± 0.065	0.178 ± 0.065	0.191 ± 0.052	Correlation
T24	RECIST lesion size measurements	Mixed	0.278 ± 0.019	0.251 ± 0.028	0.213 ± 0.044	0.296 ± 0.019	0.262 ± 0.021	Correlation
T24	RECIST lesion size measurements	Domain	0.054 ± 0.015	0.069 ± 0.008	0.060 ± 0.011	0.064 ± 0.012	0.069 ± 0.010	Ratio within 1 mm
T24	RECIST lesion size measurements	General	0.059 ± 0.008	0.073 ± 0.013	0.069 ± 0.011	0.072 ± 0.011	0.068 ± 0.006	Ratio within 1 mm
T24	RECIST lesion size measurements	Mixed	0.057 ± 0.007	0.064 ± 0.007	0.054 ± 0.014	0.064 ± 0.011	0.064 ± 0.016	Ratio within 1 mm
T24	RECIST lesion size measurements	Domain	0.309 ± 0.020	0.308 ± 0.017	0.322 ± 0.019	0.328 ± 0.017	0.338 ± 0.010	Ratio within 5 mm
T24	RECIST lesion size measurements	General	0.295 ± 0.019	0.296 ± 0.019	0.287 ± 0.018	0.278 ± 0.038	0.293 ± 0.029	Ratio within 5 mm
T24	RECIST lesion size measurements	Mixed	0.310 ± 0.014	0.301 ± 0.012	0.292 ± 0.016	0.290 ± 0.014	0.311 ± 0.005	Ratio within 5 mm

Comparison against previously reported systems

The performance of the best-performing model, RoBERTa large with domain-specific pretraining, is compared against systems reported in prior literature.

Task T2 (pulmonary nodule presence)

For this task, the selected model achieved a sensitivity of 88% ± 4% and a specificity of 92% ± 2% for identifying radiology reports with pulmonary nodules in the test set. This is lower than the rule-based system developed specifically for this dataset[1], which achieved a sensitivity of 94% (62/66) and specificity of 96% (128/134) in the same cases.

Task T7 (pulmonary nodule size presence)

For this task, the selected model achieved a sensitivity of 84% ± 5% and a specificity of 79% ± 4% for identifying the reports with nodule diameter measurements, for the 66 cases with a pulmonary nodule. This is lower than the rule-based system developed specifically for this dataset [1], which achieved a sensitivity of 90% (27/30) and specificity of 97% (31/32), for the 62 cases with true positive detections (i.e., the 62 cases described above).

Task T10 (prostate radiology suspicious lesions)

For this task, the selected model achieved an accuracy of 98% ± 0.2%. A rule-based system specifically developed for this task attained an accuracy of 99% on cases from a single hospital[2]. The AI model was tested on cases from four hospitals, which resulted in a more heterogeneous set of reports. Applying this rule-based system to the same test set resulted in an accuracy of 89.1% and a linearly weighted kappa of 0.799.

References

1. Hendrix, W. *et al.* Trends in the incidence of pulmonary nodules in chest computed tomography: 10-year results from two Dutch hospitals. *Eur Radiol* (2023) doi:10.1007/s00330-023-09826-3.
2. Bosma, J. S. *et al.* Semisupervised Learning with Report-guided Pseudo Labels for Deep Learning–based Prostate Cancer Detection Using Biparametric MRI. *Radiology: Artificial Intelligence* **5**, e230031 (2023).

Supplementary Note 4: Statistical Analysis Plan

As the primary outcome of the DRAGON (Diagnostic Report Analysis: General Optimization of NLP) study, we statistically compare the downstream performance of models pretrained with different pretraining strategies. The pretraining strategies we compare are domain-specific pretraining (using diagnostic reports), general-domain pretraining (using Wikipedia, books, etc.), and mixed-domain pretraining (general-domain followed by domain-specific pretraining). Performance is evaluated by calculating the test set performance across each architecture, each downstream task, and each fine-tuning training run. For each pretraining configuration, multiple architectures are pretrained and evaluated, and results are pooled together to compare the pretraining strategies. Downstream performance for each task is determined by fine-tuning the pretrained model using the training set of the downstream task and calculating the performance on the test set of the downstream task. This fine-tuning step is performed five times, where the training set is a different 80% of the development dataset each run.

Statistical tests are reserved for four primary outcomes only, in compliance with expert guidelines (1). To maintain the type I error while investigating multiple comparisons, all study objectives are prespecified in a hierarchical family and tested accordingly (2).

Supplementary Figure 1 shows the statistical tests for the four primary outcomes:
1A. Is domain-specific pretraining superior to general-domain pretraining?
1B. Is mixed-domain pretraining superior to general-domain pretraining?
2A. Is domain-specific pretraining superior to mixed-domain pretraining?
2B. Is mixed-domain pretraining superior to domain-specific pretraining?

Multiplicity is corrected for at each stage using the Holm–Bonferroni method, considering a base alpha value of 0.05.

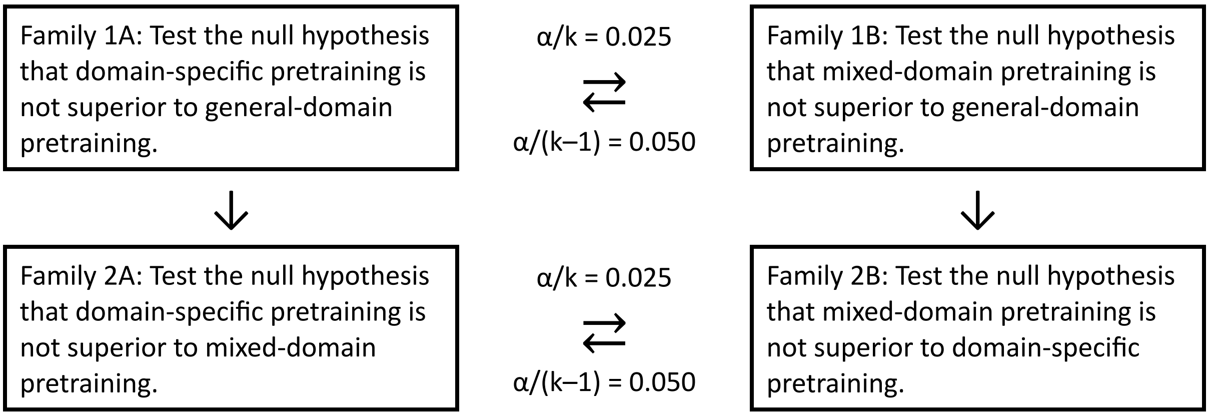

Supplementary Figure 1: Flowchart illustrating the strategic plan to test the study objectives, while maintaining the type I error rate. Significance thresholds used for family 1A and 1B are adjusted using the Holm–Bonferroni method, considering a base alpha value of 0.05. If the null hypothesis for family 1A is rejected, but that of family 1B is not rejected, then we move on to testing family 2A with an alpha value of 0.025 but do not test family 2B. Conversely, if the null hypothesis for family 1B is rejected but that of family 1A is not rejected, then we move on to testing family 2B with an alpha value of 0.025, but do not test family 2A. If the null hypotheses for both family 1A and 1B are rejected, then we move on to testing both family 2A and 2B, where the significance threshold is once again adjusted using the Holm–Bonferroni method considering a base alpha value of 0.05. If neither family 1A nor 1B are rejected, neither family 2A nor 2B will be tested.

Calculation of P Values

The primary endpoints of this study are compared using the permutation test. The efficacy of each pretraining approach is defined by the performance observed for each of the 28 tasks, with each of the five network architectures, across five fine-tuning runs (the tasks and architectures are listed below). This results in $28 \cdot 5 \cdot 5 = 700$ independent training runs and performance metrics per pretraining configuration. Performance metrics are comparable between architectures and fine-tuning runs, but not between tasks. To isolate the effect of pretraining from the effect of the architecture, we only compare performance metrics from the same architecture. As such, in $5 \times 28 = 140$ groups, the five performance metrics from the five fine-tuning runs of the baseline configuration are compared with the five performance metrics from the five fine-tuning runs of the alternative configuration. We permute the performance metrics within each of these 140 groups to obtain the null distribution of no performance difference.

The test statistic for the permutation test is the mean order-AUC across all groups, following (3):

$$S = \frac{1}{140} \sum_{i=1}^{28} \sum_{j=1}^{5} \mathrm{AUC}\{S_{ij,baseline}, S_{ij,alternative}\}$$

Where $S_{ij,baseline}$ and $S_{ij,alternative}$ each denote a set of five performance metrics, for the baseline and alternative configuration, respectively.

The P value for superiority in the context of a permutation test can be calculated directly from the null distribution. The null distribution is constructed as follows: calculate the test statistic for the observed data; randomly permute the performance metrics between modalities (baseline and alternative configurations) and recalculate the test statistic; repeat permutations and recalculation of the test statistic a large number of times (100,000) to generate a distribution under the null hypothesis. The P value is then calculated as the proportion of permuted statistics that are equal to or more extreme than the observed statistic:

$$p = \frac{1}{N} \sum_{i=1}^{N} \mathbb{1}\{\mathrm{S_i} \geq \mathrm{S}_o\}$$

Where $\mathbb{1}\{S_i \geq S_o\}$ equals 1 if the statistic (S_i) is equal to or more extreme than the observed statistic (S_o) in iteration i of the permutation test, or 0 otherwise. N is the number of permutation test iterations.

Architectures and tasks

The following five architectures are included:

1. Dutch BERT (HuggingFace: GroNLP/bert-base-dutch-cased)
2. Multi-lingual RoBERTa base (HuggingFace: xlm-roberta-base)
3. Multi-lingual RoBERTa large (HuggingFace: xlm-roberta-large)
4. English Longformer base (HuggingFace: allenai/longformer-base-4096)
5. English Longformer large (HuggingFace: allenai/longformer-large-4096)

The following 28 tasks are used to evaluate model performance:

1. Adhesion presence
2. Pulmonary nodule presence
3. Kidney abnormality identification
4. Skin histopathology case selection
5. RECIST timeline
6. Histopathology cancer origin
7. Pulmonary nodule size presence

8. Pancreatic ductal adenocarcinoma size presence
9. Pancreatic ductal adenocarcinoma diagnosis
10. Prostate radiology suspicious lesions
11. Prostate histopathology significant cancers
12. Histopathology sample origin
13. Histopathology sample type
14. Entailment diagnostic sentences
15. Colon histopathology diagnosis
16. RECIST lesion size presence
17. Pancreatic ductal adenocarcinoma attributes
18. Hip Kellgren-Lawrence scoring
19. Prostate volume extraction
20. Prostate specific antigen extraction
21. Prostate specific antigen density extraction
22. Pancreatic ductal adenocarcinoma size measurement
23. Pulmonary nodule size measurement
24. RECIST lesion size measurement
25. Anonymization
26. Medical terminology recognition
27. Prostate biopsy sampling
28. Skin histopathology diagnosis

References

1. Harrington D, D'Agostino RB, Gatsonis C, Hogan JW, Hunter DJ, Normand SLT, et al. New Guidelines for Statistical Reporting in the *Journal*. N Engl J Med. 2019 Jul 18;381(3):285–6.

2. Obuchowski NA, Bullen J. Multireader Diagnostic Accuracy Imaging Studies: Fundamentals of Design and Analysis. Radiology. 2022 Apr;303(1):26–34.

3. Bosma JS, Saha A, Hosseinzadeh M, Slootweg I, De Rooij M, Huisman H. Semisupervised Learning with Report-guided Pseudo Labels for Deep Learning–based Prostate Cancer Detection Using Biparametric MRI. Radiology: Artificial Intelligence. 2023 Sep 1;5(5):e230031.

Supplementary Note 5: Inter-annotator Agreement

To evaluate annotation consistency across the tasks included in the DRAGON benchmark, we conducted a reader study to estimate inter-annotator agreement. The original annotation workflows used to construct the DRAGON benchmark did not include formal assessment of inter-annotator reliability. These original workflows involved more extensive annotator training, with opportunities to consult clinicians on challenging cases. Moreover, many of the original annotations were produced by trained investigators with significant experience in the relevant tasks. In contrast, the readers recruited for the reader study were not involved in the original annotation process and, in general, had less specialized experience. Consequently, the inter-annotator agreement values obtained from this reader study should be interpreted as a lower bound on the consistency of the original benchmark annotations.

To enhance efficiency, certain tasks with similar annotation requirements were grouped, resulting in 16 reader study blocks. For each block, we randomly sampled 30 to 50 reports from the respective test sets. In total, 550 reports were included in the reader study. Each block was independently annotated by two readers from Radboudumc who were not involved in the original labeling process. Patient privacy was preserved by ensuring that all reports remained on premises throughout the annotation process. Reader assignment was designed so that neither of the two study readers had previously labeled the specific task, thereby enabling a comparison among three independent annotations per report: the two from the reader study and the original benchmark annotation. Due to the scale of the study and time constraints, we could not replicate the comprehensive training, expert consultation, or post-annotation consistency checks applied during the original annotation effort. As such, the inter-annotator agreement reported here reflects a conservative estimate of annotation reliability in the benchmark.

Inter-annotator agreement was quantified using Krippendorff's alpha, a chance-corrected metric that supports nominal, ordinal and numeric data, accommodates multiple raters, and is robust to missing values. This makes Krippendorff's alpha suitable for a wide range of task types, and we used it for all case-level annotation tasks (T1–T24). Krippendorff's alpha was calculated using the Python package krippendorff[1], version 0.8.1. For named entity recognition (NER) tasks (T25–T28), we computed F1 scores. Additionally, we also calculated for each reader the number of annotations that matched the original benchmark labels. Specifically, this agreement count was defined as follows:

- For single-label binary classification, single-label multi-class classification, and single-label regression tasks: the number of correctly matched cases.
- For multi-label binary classification, multi-label multi-class and multi-label regression tasks: the number of correctly matched labels.
- For NER tasks: the number of correctly matched entities (TP). For NER tasks the number of false positives (FP) and false negatives (FN) was also reported.

The average Krippendorff's alpha (tasks T1–T24) was 0.859 and the average F1 score (tasks T25–T28) was 0.860. The full task-level agreement overview is given in Supplementary Table 4 and Supplementary Table 5. The agreement for each model with the benchmark labels is shown in Supplementary Table 6. For task T22, agreement was calculated using only 9 available reports due to the report sampling setup of the reader study. For all other tasks, the same set of reports as in the reader study was used to compute agreement.

These findings confirm the high quality of the DRAGON benchmark annotations. In particular, the strong agreement of RoBERTa large with domain-specific pretraining on task T26 (α=0.97 ± 0.01) and its higher agreement on Task18 (α=0.70 ± 0.04) indicate that the lower agreement observed in the reader study was likely due to less training among the readers compared to the original annotators.

Supplementary Table 4: Summary of inter-annotator agreement across DRAGON benchmark tasks, measured using Krippendorff's alpha. Each task includes the number of reports evaluated, the level of measurement used for Krippendorff's alpha (e.g., nominal, ordinal), and accuracy metrics for both readers. Accuracy is computed as the number of evaluation units (e.g., cases or labels) with exact matches to the original benchmark annotation, divided by the total number of units.

ID	Name	Number of Reports	Krippendorff's Alpha – Level of Measurement	Krippendorff's Alpha	Reader 1: Exact Matches / Total Units (Accuracy)	Reader 2: Exact Matches / Total Units (Accuracy)
T1	Adhesion presence	30	nominal	1.000	30/30 (100.0%)	30/30 (100.0%)
T2	Pulmonary nodule presence	30	nominal	0.888	29/30 (96.7%)	29/30 (96.7%)
T3	Kidney abnormality identification	30	nominal	0.863	29/30 (96.7%)	27/30 (90.0%)
T4	Skin histopathology case selection	30	nominal	1.000	30/30 (100.0%)	30/30 (100.0%)
T5	RECIST timeline	30	nominal	0.823	27/30 (90.0%)	29/30 (96.7%)
T6	Histopathology cancer origin	30	nominal	0.333	24/30 (80.0%)	25/30 (83.3%)
T7	Pulmonary nodule size presence	5	nominal	1.000	5/5 (100.0%)	5/5 (100.0%)
T8	PDAC size presence	30	nominal	0.889	30/30 (100.0%)	29/30 (96.7%)
T9	PDAC diagnosis	30	nominal	0.817	28/30 (93.3%)	25/30 (83.3%)
T10	Prostate radiology suspicious lesions	30	ordinal	0.996	30/30 (100.0%)	29/30 (96.7%)
T11	Prostate histopathology significant cancers	30	ordinal	0.982	30/30 (100.0%)	28/30 (93.3%)
T12	Histopathology tissue type	30	nominal	0.748	26/30 (86.7%)	26/30 (86.7%)
T13	Histopathology tissue origin	30	nominal	0.769	25/30 (83.3%)	25/30 (83.3%)
T14	Entailment diagnostic sentences	50	ordinal	0.550	35/50 (70.0%)	34/50 (68.0%)
T15	Colon histopathology diagnosis	30	nominal per label, then averaged	0.875	201/210 (95.7%)	207/210 (98.6%)
T16	RECIST lesion size presence	30	pooled then nominal	0.978	150/150 (100.0%)	148/150 (98.7%)
T17	PDAC attributes	30	nominal	0.677	53/60 (88.3%)	49/60 (81.7%)
T18	Hip Kellgren-Lawrence scoring	30	pooled then nominal	0.557	42/60 (70.0%)	37/60 (61.7%)
T19	Prostate volume measurement	30	interval	1.000	30/30 (100.0%)	30/30 (100.0%)
T20	Prostate specific antigen measurement	30	interval	0.999	29/30 (96.7%)	29/30 (96.7%)
T21	Prostate specific antigen density measurement	30	interval	1.000	30/30 (100.0%)	30/30 (100.0%)
T22	PDAC size measurement	27	interval	0.875	23/27 (85.2%)	24/27 (88.9%)
T23	Pulmonary nodule size measurement	4	interval	1.000	4/4 (100.0%)	4/4 (100.0%)
T24	RECIST lesion size measurements	30	pooled then interval	0.997	105/109 (96.3%)	104/109 (95.4%)

Supplementary Table 5: Summary of inter-annotator agreement across DRAGON benchmark named entity recognition tasks, measured using the micro-averaged F1 score. Each task includes the number of reports evaluated, the average F1 score across both readers, and detailed performance metrics for each reader including the number of true positives (TP), false positives (FP), false negatives (FN), and corresponding F1 score.

ID	Name	Number of Reports	Average F1 Score	Reader 1: TP / FP / FN (F1 Score)	Reader 2: TP / FP / FN (F1 Score)
T25	Anonymization	30	0.784	87, 29, 10 (81.7%)	80, 36, 17 (75.1%)
T26	Medical terminology recognition	30	0.765	921, 224, 191 (81.6%)	852, 422, 260 (71.4%)
T27	Prostate biopsy sampling	30	0.965	168, 2, 2 (98.8%)	161, 11, 9 (94.2%)
T28	Skin histopathology diagnosis	50	0.917	245, 27, 29 (89.7%)	262, 15, 12 (95.1%)

Supplementary Table 6: Agreement scores for each model across DRAGON benchmark tasks, presented as mean ± standard deviation (across five fine-tuning runs with different development data partitions). Agreement was quantified using Krippendorff's alpha for tasks T1–T24 and micro-averaged F1 score for tasks T25–T28 (as described in Supplementary Table 4 and Supplementary Table 5). For task T22, agreement was calculated based on only 9 available reports due to the report sampling setup of the reader study.

ID	Name	Pretraining strategy	BERT base	Longformer base	Longformer large	RoBERTa base	RoBERTa large	Reader Study
T1	Adhesion presence	Domain	0.75 ± 0.11	0.80 ± 0.09	0.77 ± 0.11	0.91 ± 0.05	0.94 ± 0.06	1.000
T1	Adhesion presence	General	0.96 ± 0.06	0.40 ± 0.31	0.52 ± 0.26	0.42 ± 0.47	0.86 ± 0.11	1.000
T1	Adhesion presence	Mixed	0.96 ± 0.06	0.82 ± 0.17	0.89 ± 0.07	0.88 ± 0.11	0.96 ± 0.06	1.000
T2	Pulmonary nodule presence	Domain	0.83 ± 0.03	0.65 ± 0.06	0.66 ± 0.11	0.83 ± 0.03	0.84 ± 0.01	0.888
T2	Pulmonary nodule presence	General	0.44 ± 0.12	0.18 ± 0.25	0.15 ± 0.19	0.59 ± 0.16	0.74 ± 0.06	0.888
T2	Pulmonary nodule presence	Mixed	0.64 ± 0.05	0.61 ± 0.05	0.76 ± 0.06	0.84 ± 0.00	0.72 ± 0.05	0.888
T3	Kidney abnormality identification	Domain	0.31 ± 0.08	0.12 ± 0.07	0.66 ± 0.07	0.25 ± 0.08	0.54 ± 0.31	0.863
T3	Kidney abnormality identification	General	0.23 ± 0.25	-0.00 ± 0.26	-0.00 ± 0.30	0.14 ± 0.27	0.63 ± 0.13	0.863
T3	Kidney abnormality identification	Mixed	0.32 ± 0.09	-0.34 ± 0.09	-0.04 ± 0.24	0.11 ± 0.12	0.15 ± 0.14	0.863
T4	Skin histopathology case selection	Domain	1.00 ± 0.00	1.00 ± 0.00	1.00 ± 0.00	1.00 ± 0.00	1.00 ± 0.00	1.000
T4	Skin histopathology case selection	General	1.00 ± 0.00	1.00 ± 0.00	0.98 ± 0.05	0.93 ± 0.06	1.00 ± 0.00	1.000
T4	Skin histopathology case selection	Mixed	1.00 ± 0.00	1.00 ± 0.00	1.00 ± 0.00	1.00 ± 0.00	1.00 ± 0.00	1.000
T5	RECIST timeline	Domain	0.83 ± 0.04	0.85 ± 0.03	0.85 ± 0.03	0.81 ± 0.03	0.80 ± 0.05	0.823
T5	RECIST timeline	General	0.83 ± 0.04	0.74 ± 0.09	0.61 ± 0.23	0.15 ± 0.44	0.80 ± 0.09	0.823
T5	RECIST timeline	Mixed	0.87 ± 0.05	0.79 ± 0.03	0.70 ± 0.12	0.84 ± 0.04	0.83 ± 0.04	0.823
T6	Histopathology cancer origin	Domain	-0.11 ± 0.00	-0.11 ± 0.00	-0.11 ± 0.00	-0.11 ± 0.00	-0.11 ± 0.00	0.333
T6	Histopathology cancer origin	General	-0.11 ± 0.00	-0.11 ± 0.00	-0.11 ± 0.00	-0.11 ± 0.00	-0.11 ± 0.00	0.333
T6	Histopathology cancer origin	Mixed	-0.11 ± 0.00	-0.11 ± 0.00	-0.11 ± 0.00	-0.11 ± 0.00	-0.11 ± 0.00	0.333
T7	Pulmonary nodule size presence	Domain	0.25 ± 0.23	0.31 ± 0.14	0.86 ± 0.20	0.59 ± 0.27	0.85 ± 0.34	1.000
T7	Pulmonary nodule size presence	General	0.64 ± 0.00	0.39 ± 0.57	-0.16 ± 0.07	0.62 ± 0.46	0.17 ± 0.40	1.000
T7	Pulmonary nodule size presence	Mixed	0.18 ± 0.15	0.40 ± 0.43	0.35 ± 0.39	0.33 ± 0.17	0.33 ± 0.29	1.000
T8	PDAC size presence	Domain	-0.04 ± 0.00	-0.04 ± 0.00	-0.05 ± 0.01	-0.04 ± 0.00	0.04 ± 0.17	0.889
T8	PDAC size presence	General	-0.04 ± 0.00	-0.04 ± 0.00	-0.04 ± 0.00	-0.04 ± 0.00	-0.04 ± 0.00	0.889
T8	PDAC size presence	Mixed	-0.04 ± 0.00	-0.04 ± 0.00	-0.04 ± 0.00	-0.04 ± 0.00	-0.04 ± 0.00	0.889
T9	PDAC diagnosis	Domain	0.75 ± 0.03	0.61 ± 0.03	0.72 ± 0.07	0.78 ± 0.04	0.80 ± 0.07	0.817
T9	PDAC diagnosis	General	0.70 ± 0.04	0.67 ± 0.03	0.60 ± 0.12	0.70 ± 0.08	0.73 ± 0.04	0.817
T9	PDAC diagnosis	Mixed	0.79 ± 0.10	0.72 ± 0.13	0.81 ± 0.07	0.75 ± 0.06	0.82 ± 0.06	0.817
T10	Prostate radiology suspicious lesions	Domain	0.99 ± 0.00	0.99 ± 0.00	0.99 ± 0.00	1.00 ± 0.00	1.00 ± 0.00	0.996
T10	Prostate radiology suspicious lesions	General	1.00 ± 0.00	1.00 ± 0.00	1.00 ± 0.00	1.00 ± 0.00	1.00 ± 0.00	0.996
T10	Prostate radiology suspicious lesions	Mixed	1.00 ± 0.00	1.00 ± 0.00	1.00 ± 0.00	1.00 ± 0.00	1.00 ± 0.00	0.996
T11	Prostate histopathology significant cancers	Domain	0.96 ± 0.01	0.96 ± 0.03	0.86 ± 0.03	0.97 ± 0.02	0.95 ± 0.03	0.982
T11	Prostate histopathology significant cancers	General	0.90 ± 0.02	0.99 ± 0.00	0.99 ± 0.00	0.97 ± 0.03	0.96 ± 0.06	0.982
T11	Prostate histopathology significant cancers	Mixed	0.97 ± 0.02	0.99 ± 0.00	0.97 ± 0.03	0.98 ± 0.01	0.98 ± 0.01	0.982
T12	Histopathology tissue type	Domain	0.18 ± 0.08	0.12 ± 0.04	0.25 ± 0.08	0.14 ± 0.04	0.29 ± 0.11	0.748
T12	Histopathology tissue type	General	0.15 ± 0.07	0.08 ± 0.28	0.22 ± 0.26	-0.03 ± 0.16	0.15 ± 0.15	0.748
T12	Histopathology tissue type	Mixed	0.17 ± 0.12	0.06 ± 0.20	0.70 ± 0.10	-0.09 ± 0.07	0.05 ± 0.10	0.748
T13	Histopathology tissue origin	Domain	0.07 ± 0.24	-0.12 ± 0.00	0.45 ± 0.16	-0.12 ± 0.00	0.79 ± 0.10	0.769
T13	Histopathology tissue origin	General	0.26 ± 0.25	0.09 ± 0.26	0.51 ± 0.18	-0.12 ± 0.00	0.27 ± 0.24	0.769
T13	Histopathology tissue origin	Mixed	0.73 ± 0.22	0.11 ± 0.42	0.64 ± 0.26	0.02 ± 0.32	0.87 ± 0.14	0.769
T14	Entailment diagnostic sentences	Domain	0.55 ± 0.03	0.56 ± 0.05	0.46 ± 0.07	0.63 ± 0.04	0.59 ± 0.06	0.550
T14	Entailment diagnostic sentences	General	0.53 ± 0.03	0.51 ± 0.07	0.45 ± 0.27	0.53 ± 0.07	0.21 ± 0.51	0.550
T14	Entailment diagnostic sentences	Mixed	0.56 ± 0.08	0.57 ± 0.09	0.28 ± 0.49	0.56 ± 0.09	0.58 ± 0.07	0.550

T15	Colon histopathology diagnosis	Domain	0.88 ± 0.08	0.89 ± 0.07	0.91 ± 0.07	0.93 ± 0.03	0.92 ± 0.03	0.875
T15	Colon histopathology diagnosis	General	0.94 ± 0.04	0.98 ± 0.02	1.00 ± 0.00	0.86 ± 0.11	1.00 ± 0.00	0.875
T15	Colon histopathology diagnosis	Mixed	0.99 ± 0.01	1.00 ± 0.00	0.99 ± 0.03	0.96 ± 0.04	1.00 ± 0.00	0.875
T16	RECIST lesion size presence	Domain	0.46 ± 0.02	0.47 ± 0.03	0.64 ± 0.02	0.55 ± 0.03	0.60 ± 0.03	0.978
T16	RECIST lesion size presence	General	0.49 ± 0.03	0.40 ± 0.02	0.48 ± 0.05	0.41 ± 0.04	0.47 ± 0.04	0.978
T16	RECIST lesion size presence	Mixed	0.45 ± 0.02	0.40 ± 0.02	0.45 ± 0.04	0.47 ± 0.03	0.44 ± 0.03	0.978
T17	PDAC attributes	Domain	-0.09 ± 0.00	-0.09 ± 0.00	0.05 ± 0.07	-0.09 ± 0.00	0.23 ± 0.31	0.677
T17	PDAC attributes	General	-0.09 ± 0.00	-0.09 ± 0.00	-0.07 ± 0.04	-0.09 ± 0.00	-0.09 ± 0.00	0.677
T17	PDAC attributes	Mixed	-0.09 ± 0.00	-0.09 ± 0.00	-0.08 ± 0.02	-0.07 ± 0.05	-0.00 ± 0.06	0.677
T18	Hip Kellgren-Lawrence scoring	Domain	0.71 ± 0.01	0.65 ± 0.02	0.62 ± 0.02	0.69 ± 0.02	0.70 ± 0.04	0.557
T18	Hip Kellgren-Lawrence scoring	General	0.66 ± 0.03	0.62 ± 0.03	0.64 ± 0.04	0.47 ± 0.07	0.69 ± 0.04	0.557
T18	Hip Kellgren-Lawrence scoring	Mixed	0.69 ± 0.01	0.64 ± 0.03	0.67 ± 0.05	0.66 ± 0.02	0.71 ± 0.02	0.557
T19	Prostate volume measurement	Domain	1.00 ± 0.00	1.00 ± 0.00	0.93 ± 0.04	1.00 ± 0.00	1.00 ± 0.00	1.000
T19	Prostate volume measurement	General	0.99 ± 0.00	0.99 ± 0.01	1.00 ± 0.00	0.96 ± 0.03	0.99 ± 0.01	1.000
T19	Prostate volume measurement	Mixed	1.00 ± 0.00	1.00 ± 0.00	1.00 ± 0.00	0.99 ± 0.00	0.98 ± 0.01	1.000
T20	Prostate specific antigen measurement	Domain	0.64 ± 0.08	0.39 ± 0.24	0.97 ± 0.02	0.65 ± 0.08	0.99 ± 0.02	0.999
T20	Prostate specific antigen measurement	General	0.38 ± 0.04	0.92 ± 0.05	0.96 ± 0.02	0.56 ± 0.37	0.98 ± 0.02	0.999
T20	Prostate specific antigen measurement	Mixed	0.38 ± 0.09	0.95 ± 0.04	0.82 ± 0.31	0.84 ± 0.06	0.99 ± 0.01	0.999
T21	Prostate specific antigen density measurement	Domain	0.50 ± 0.09	0.25 ± 0.16	0.16 ± 0.03	0.66 ± 0.05	0.86 ± 0.05	1.000
T21	Prostate specific antigen density measurement	General	0.25 ± 0.05	0.59 ± 0.17	0.83 ± 0.07	0.64 ± 0.07	0.70 ± 0.09	1.000
T21	Prostate specific antigen density measurement	Mixed	0.49 ± 0.14	0.69 ± 0.08	0.82 ± 0.13	0.65 ± 0.07	0.83 ± 0.12	1.000
T22	PDAC size measurement	Domain	0.03 ± 0.05	0.04 ± 0.04	0.05 ± 0.09	0.15 ± 0.07	0.12 ± 0.14	0.875
T22	PDAC size measurement	General	-0.10 ± 0.03	0.04 ± 0.04	0.06 ± 0.10	0.07 ± 0.05	0.07 ± 0.06	0.875
T22	PDAC size measurement	Mixed	0.03 ± 0.08	-0.07 ± 0.09	-0.05 ± 0.14	0.01 ± 0.05	0.07 ± 0.13	0.875
T23	Pulmonary nodule size measurement	Domain	0.35 ± 0.13	0.22 ± 0.02	0.37 ± 0.17	0.37 ± 0.08	0.65 ± 0.09	1.000
T23	Pulmonary nodule size measurement	General	0.48 ± 0.14	0.14 ± 0.05	0.23 ± 0.17	0.13 ± 0.02	0.14 ± 0.02	1.000
T23	Pulmonary nodule size measurement	Mixed	0.27 ± 0.14	0.29 ± 0.14	0.43 ± 0.16	0.41 ± 0.09	0.63 ± 0.23	1.000
T24	RECIST lesion size measurements	Domain	0.10 ± 0.02	0.07 ± 0.02	0.12 ± 0.03	0.14 ± 0.02	0.16 ± 0.03	0.997
T24	RECIST lesion size measurements	General	0.10 ± 0.03	0.03 ± 0.02	0.06 ± 0.05	0.03 ± 0.02	0.04 ± 0.02	0.997
T24	RECIST lesion size measurements	Mixed	0.10 ± 0.01	0.08 ± 0.04	0.07 ± 0.04	0.13 ± 0.02	0.11 ± 0.02	0.997
T25	Anonymization	Domain	0.82 ± 0.01	0.82 ± 0.02	0.84 ± 0.01	0.83 ± 0.02	0.89 ± 0.01	0.784
T25	Anonymization	General	0.84 ± 0.01	0.85 ± 0.02	0.00 ± 0.00	0.92 ± 0.01	0.97 ± 0.02	0.784
T25	Anonymization	Mixed	0.88 ± 0.01	0.88 ± 0.02	0.00 ± 0.00	0.89 ± 0.02	0.93 ± 0.04	0.784
T26	Medical terminology recognition	Domain	0.65 ± 0.01	0.60 ± 0.03	0.80 ± 0.01	0.66 ± 0.00	0.84 ± 0.01	0.765
T26	Medical terminology recognition	General	0.70 ± 0.01	0.68 ± 0.00	0.62 ± 0.08	0.71 ± 0.01	0.81 ± 0.03	0.765
T26	Medical terminology recognition	Mixed	0.72 ± 0.01	0.70 ± 0.01	0.71 ± 0.07	0.71 ± 0.00	0.79 ± 0.00	0.765
T27	Prostate biopsy sampling	Domain	0.74 ± 0.08	0.33 ± 0.04	0.64 ± 0.01	0.91 ± 0.03	0.97 ± 0.01	0.965
T27	Prostate biopsy sampling	General	0.96 ± 0.01	0.95 ± 0.01	0.00 ± 0.01	0.94 ± 0.04	0.94 ± 0.02	0.965
T27	Prostate biopsy sampling	Mixed	0.96 ± 0.01	0.97 ± 0.01	0.01 ± 0.01	0.96 ± 0.01	0.97 ± 0.01	0.965
T28	Skin histopathology diagnosis	Domain	0.46 ± 0.01	0.31 ± 0.14	0.45 ± 0.01	0.49 ± 0.05	0.63 ± 0.05	0.917
T28	Skin histopathology diagnosis	General	0.47 ± 0.04	0.31 ± 0.20	0.00 ± 0.00	0.42 ± 0.09	0.48 ± 0.01	0.917
T28	Skin histopathology diagnosis	Mixed	0.38 ± 0.21	0.37 ± 0.20	0.00 ± 0.00	0.49 ± 0.01	0.53 ± 0.03	0.917

References

1. Castro, S. Fast Krippendorff: Fast computation of Krippendorff's alpha agreement measure. (2017).

Supplementary Note 6: Anonymisation

Protected health information (PHI) was automatically identified using institutional PHI detection software. For a subset of reports, automatically detected PHI was subsequently refined through manual review. Additionally, manual anonymization was performed on another subset of reports by removing PHI. Detected PHI was replaced with realistic surrogates[1].

The performance of the PHI detection software was evaluated on an institutional dataset containing 1,600 clinical reports (Supplementary Table 7). Beyond the PHI categories listed, we also anonymized hospital names, patient ages, report identifiers (e.g., pathology T-numbers), hospital accreditation numbers, clinical trial names, and hospital-specific URLs. Identification and anonymization procedures involved manual, semi-automatic, and fully automatic approaches designed to optimize both precision and recall throughout the dataset.

The anonymization protocol was formally reviewed and approved by our institutional Data Protection Office after a thorough Data Protection Impact Assessment (DPIA) related to the secure transfer of data to trusted third-party processors where it remains sequestered.

Supplementary Table 7: Performance metrics of the PHI detection software on the institutional dataset of 1,600 clinical reports. Metrics include counts, precision, and recall for each PHI category detected.

PHI Tag	Count	Precision	Recall
Name	129	0.97 (124/128)	0.96 (124/129)
Date	1837	0.97 (1779/1843)	0.97 (1779/1837)
Time	81	0.97 (77/79)	0.95 (77/81)
Phone number	18	0.89 (16/18)	0.89 (16/18)
Patient number	0	0.0 (0/0)	0.0 (0/0)
Z-number	0	0.0 (0/0)	0.0 (0/0)
Location	5	1.0 (2/2)	0.4 (2/5)
Total	2072	-	-

References

1. Carrell, D. *et al.* Hiding in plain sight: use of realistic surrogates to reduce exposure of protected health information in clinical text. *J Am Med Inform Assoc* **20**, 342–348 (2013).

Supplementary Note 7: Tasks in the DRAGON Benchmark

Task 1: Adhesion presence

Background: Chronic abdominal pain after surgery is affecting many patients worldwide, without clearly established diagnostic and treatment paths[1]. Cine-MRI is a relatively novel modality, which can be used to diagnose abdominal adhesions, as introduced by Lienemann et al. in 2000[2]. Patients push their belly slowly in and out, to generate as much visceral motion as possible. Radiologists detect adhesions by looking for irregular motion patterns and anatomy. In the specialized center for chronic postoperative pain at the Radboud University Medical Center and the Rijnstate Hospital, cine-MRI is an integral part of the patient pathway[3].

Task: Predict adhesion presence (binary) based on the radiology report. Provide your prediction as a confidence score between 0 and 1, where 0 corresponds to False (no adhesion) and 1 corresponds to True (adhesion described).

Dataset: Data was gathered retrospectively and the requirement for written informed consent was waived by the Institutional Review Board (METC Oost-Nederland, registration number 19082). All patients were admitted to our specialized center for managing chronic postoperative pain. This center operates through a partnership between the Radboud University Medical Center and Rijnstate Hospital in The Netherlands. We collected all cine-MRI studies conducted between 1 January 2012 and 31 December 2020, resulting in 563 studies (527 patients). The presence of adhesions in each study was determined by an experienced researcher based on the radiology reports.

Dataset splits: 30% of patients were randomly selected for the test set (166 cases) and the remaining 70% of patients (397 cases) were used for model development with 5-fold cross-validation.

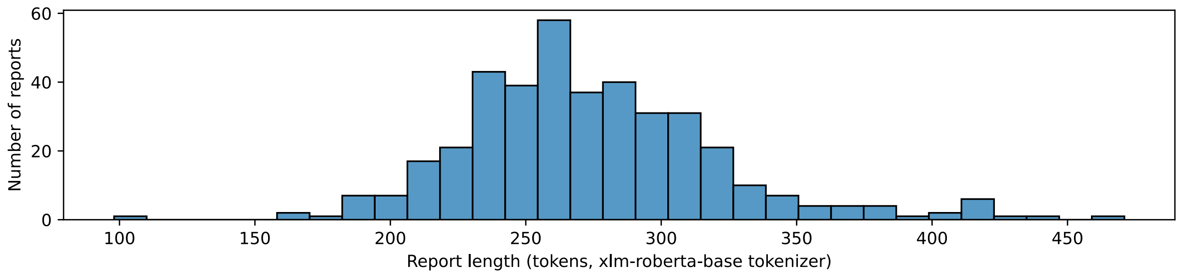

Supplementary Figure 2: Distribution of report lengths. The reports in the development dataset have a median length of 267 (95% CI: 192, 399) [min 98, max 471] tokens with the xlm-roberta-base tokenizer.

Metric: The labels for this task are binary. The frequency of the labels is imbalanced. We use the Area Under the Receiver Operating Characteristic Curve (AUROC) to evaluate model performance.

Supplementary References

1. Ten Broek, R. P. G. *et al.* Burden of adhesions in abdominal and pelvic surgery: systematic review and met-analysis. *BMJ* **347**, f5588–f5588 (2013).
2. Lienemann, A., Sprenger, D., Steitz, H. O., Korell, M. & Reiser, M. Detection and Mapping of Intraabdominal Adhesions by Using Functional Cine MR Imaging: Preliminary Results. *Radiology* **217**, 421–425 (2000).

3. Van Den Beukel, B. A. W. *et al.* A Shared Decision Approach to Chronic Abdominal Pain Based on Cine-MRI: A Prospective Cohort Study. *American Journal of Gastroenterology* **113**, 1229–1237 (2018).

Example

Label: True

Notes: the report describes an adhesion ("Adhesies van dunnedarmlis in het kleine bekken.")

Anonymous sample report:
###############################
DISCLAIMER:
THIS REPORT HAS BEEN ANONYMIZED BY REPLACING PATIENT HEALTH INFORMATION WITH RANDOM SURROGATES.
ANY RESEMBLANCE TO REAL PERSONS, LIVING OR DEAD, IS PURELY COINCIDENTAL.
###############################

Medische gegevens:
Status na sectio en appendectomie, gecompliceerd door abces. Pijnklachten met name links bovenin de buik. Adhesies? Anderszins?

MRI cine abdomen.
Geen gedilateerde darmlissen. Geen aanwijzingen voor adhesies tussen darm pakket en de voorste buikwand. Adhesies in het kleine bekken tussen darmlissen en het blaasdak en uterus. Geen aanwijzingen voor adhesies tussen darmlissen onderling.

Conclusie.
Adhesies van dunnedarmlis in het kleine bekken.

Task 2: Pulmonary nodule presence

Background: Outside a screening setting, early-stage lung cancer is generally detected after the detection of an incidental pulmonary nodule in a clinically ordered chest CT scan[1,2]. Trend analysis of the incidence of pulmonary nodules is important, because these statistics are essential in clinical decision-making and risk communication between physicians and patients with such a nodule[3]. Nodule findings are stored in unstructured radiology reports, making them difficult to extract for analysis. For large-scale trend analysis, it is unfeasible to manually analyse the reports and an automated solution would be required. Automated extraction of nodule findings could also facilitate dataset curation for the development of deep learning-based pulmonary nodule detection systems[4]. Nodule size is an important risk factor for malignancy and should therefore be extracted from the reports[5,6].

Task: Predict whether a pulmonary nodule is described in the radiology report. Provide your prediction as a confidence score between 0 and 1, where 0 corresponds to False (no pulmonary nodule described) and 1 corresponds to True (pulmonary nodule present).

Dataset: Hendrix et al.[1] created a dataset for nodule trend analysis with chest CT reports from two Dutch hospitals, acquired between 1 January 2008 and 31 December 2019 from Radboudumc and Jeroen Bosch Ziekenhuis. This dataset consists of a development set and an independent test set: the development set contains 1000 randomly sampled radiology reports (500 per hospital). The test set contains 200 randomly sampled reports (100 per hospital). Each report was given a label indicating (1) whether a pulmonary nodule was reported and (2) the diameter of the largest reported nodule if available (otherwise, a missing value was registered). All pulmonary nodules were included with a maximum diameter of 30 mm, regardless of morphology, type (i.e., solid, part-solid, non-solid, calcified, or perifissural), and malignancy status.

Dataset splits: The test and development datasets were kept the same as in the study of Hendrix et al.[1]. This selected 200 cases for testing and 1000 cases for development. We used the development dataset for 5-fold cross-validation.

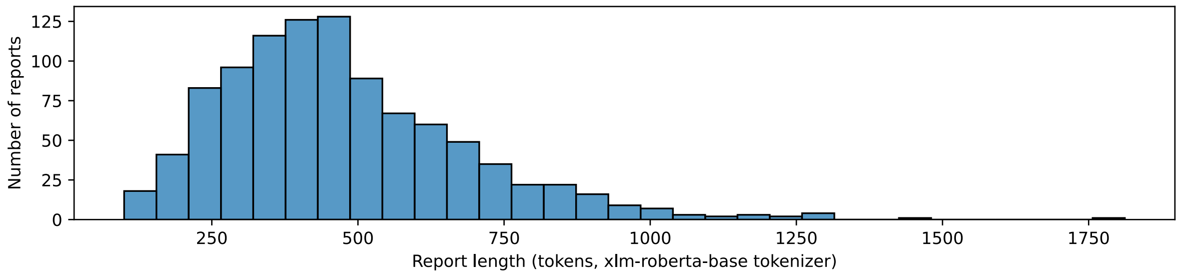

Supplementary Figure 3: Distribution of report lengths. The reports in the development dataset have a median length of 440 (95% CI: 167, 968) [min 100, max 1812] tokens with the xlm-roberta-base tokenizer.

Metric: The labels for this task are binary. The frequency of the labels is unbalanced. We use the Area Under the Receiver Operating Characteristic Curve (AUROC) to evaluate model performance.

Supplementary References

1. Hendrix, W. *et al.* Trends in the incidence of pulmonary nodules in chest computed tomography: 10-year results from two Dutch hospitals. *Eur Radiol* (2023) doi:10.1007/s00330-023-09826-3.
2. Gould, M. K. *et al.* Recent Trends in the Identification of Incidental Pulmonary Nodules. *American Journal of Respiratory and Critical Care Medicine* **192**, 1208–1214 (2015).

3. Wiener, R. S., Gould, M. K., Woloshin, S., Schwartz, L. M. & Clark, J. A. What Do You Mean, a Spot?: A Qualitative Analysis of Patients' Reactions to Discussions With Their Physicians About Pulmonary Nodules. *CHEST* **143**, 672–677 (2013).
4. Gu, Y. *et al.* A survey of computer-aided diagnosis of lung nodules from CT scans using deep learning. *Computers in Biology and Medicine* **137**, 104806 (2021).
5. MacMahon, H. *et al.* Guidelines for management of incidental pulmonary nodules detected on CT images: From the Fleischner Society 2017. *Radiology* **284**, 228–243 (2017).
6. Callister, M. E. J. *et al.* British thoracic society guidelines for the investigation and management of pulmonary nodules. *Thorax* **70**, ii1–ii54 (2015).

Example

Label: True

Notes: A pulmonary nodule is described in this report ("Meerdere aspecifieke longnoduli, vooral in de rechter onderkwab.").

Anonymous sample report:
################################
DISCLAIMER:
THIS REPORT HAS BEEN ANONYMIZED BY REPLACING PATIENT HEALTH INFORMATION WITH RANDOM SURROGATES.
ANY RESEMBLANCE TO REAL PERSONS, LIVING OR DEAD, IS PURELY COINCIDENTAL.
################################
Vraagstelling: Aanvraag voor aanvullende CT van de thorax, abdomen en nieren om de linker nier en mogelijke metastasen te evalueren.
Postcontrastonderzoek: Ter vergelijking met de scans van 13-12-2009.
Thorax:
- Geen veranderingen in het pulmonale beeld met sterke aanwijzingen voor aanwezigheid van longmetastasen.
- Mogelijke kleine bronchiëctasieën, maar geen verandering ten opzichte van voorgaande scans.
- Geen pleuravocht aanwezig.
- Mediastinum ongewijzigd zonder pathologisch vergrote klieren.
- Ongewijzigde lymfklier rechts axillair.
- Meerdere aspecifieke longnoduli, vooral in de rechter onderkwab.
Abdomen:
- Geen veranderingen waargenomen.
- Geen pathologisch vergrote klieren of aanwijzingen voor metastasen.
- Geen duidelijke afwijkingen zichtbaar intra-abdominaal.
- Beperkt intra-abdominaal vet.
Conclusie: Geen veranderingen ten opzichte van de scans van 13-12-2009.
Autorisatiedatum: 23-05-2023
Contrastmiddel: Optiray 300, 98 cc.

Task 3: Kidney abnormality identification

Background: In the United States in 2023, kidney cancer and renal pelvis cancer have emerged as the sixth most prevalent types of malignancies[1]. Early diagnosis plays a pivotal role in enhancing patient survival rates, as timely intervention through partial or full nephrectomy can effectively manage tumors while preserving a relatively high quality of life for afflicted individuals. Utilizing computed tomography (CT) scans for the discrimination of healthy kidney tissue from renal cell carcinoma offers diagnostic advantages and facilitates the identification of additional abnormalities such as cysts and kidney stones. Implementing a classifier trained to detect these anomalies holds the potential to streamline radiologist workflows. However, the manual labeling of medical images for training purposes remains a laborious and time-intensive task. Alternatively, extracting such information from medical reports presents a more streamlined approach, enabling training on a larger dataset.

Task: Classify whether any form of kidney abnormality is mentioned in the radiology report. Provide your prediction as a confidence score between 0 and 1, where 0 corresponds to False (no abnormality) and 1 corresponds to True (abnormality present).

Dataset: The Radboudumc has an internal dataset of about 300.000 Thorax-Abdomen CT scans along with their respective radiology reports. Cases were manually analyzed by Luc Builtjes (PhD candidate) and classified as either normal or abnormal. Cases were selected to form a balanced dataset with 300 normal and 300 abnormal cases by randomly sampling from the dataset and performing the classification, or opting to skip a case when classification was unclear. After 300 normal cases had been reached, additional normal cases were discarded until 300 abnormal cases were found. Abnormalities include renal cell carcinoma, angiomyolipoma, cysts, kidney stones, conjoined kidneys, cases with partial or full nephrectomy, and several other rare abnormalities.

Dataset splits: 30% of patients were randomly selected for the test set (183 cases) and the remaining 70% of patients (417 cases) were used for model development with 5-fold cross-validation.

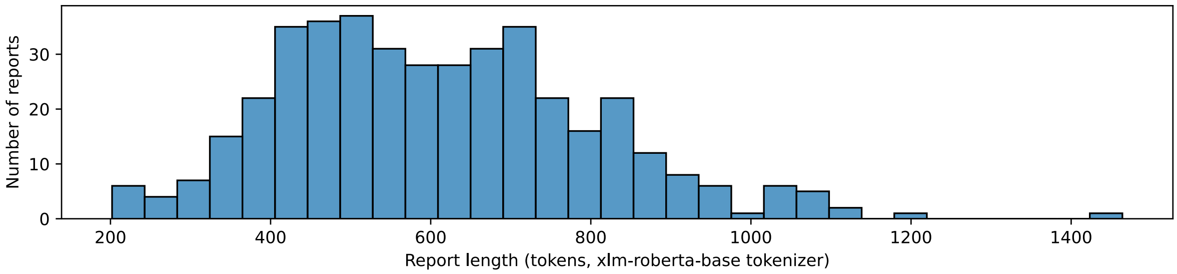

Supplementary Figure 4: Distribution of report lengths. The reports in the development dataset have a median length of 590 (95% CI: 289, 1031) [min 202, max 1464] tokens with the xlm-roberta-base tokenizer.

Metric: The labels for this task are binary. The frequency of the labels is balanced. Therefore, we use the Area Under the Receiver Operating Characteristic Curve (AUROC) to evaluate model performance.

Supplementary References

1. Siegel, R. L., Miller, K. D., Wagle, N. S. & Jemal, A. Cancer statistics, 2023. *CA A Cancer J Clinicians* **73**, 17–48 (2023).

Example

Label: True

Notes: a kidney abnormality is described, in this case both kidneys were removed ("status na bilaterale nefrectomie").

Anonymous sample report:
################################
DISCLAIMER:
THIS REPORT HAS BEEN ANONYMIZED BY REPLACING PATIENT HEALTH INFORMATION WITH RANDOM SURROGATES.
ANY RESEMBLANCE TO REAL PERSONS, LIVING OR DEAD, IS PURELY COINCIDENTAL.
################################
klinische gegevens: status na bilaterale nefrectomie. gemetastaseerd medullair schildkliercarcinoom. evaluatie van progressie?
verslag:
ct-thorax en abdomen: protocollair gescand met 85 ml iomeron 300. vergelijking met onderzoek van 3 mei is mogelijk.
geen adenopathie in de axillaire regio; geen veranderingen. er is een bekende sterk vergrote schildklierkwab aan de rechterzijde met doorgroei in het mediastinum. dit veroorzaakt een deviatie van de trachea naar links en compressie van de trachea. de maximale afmeting van de schildklierkwab is momenteel 43 mm, voorheen was dit 35 mm. geen toename van pericardvocht of pleuravocht. mediastinaal zijn er bekende vergrote klieren aanwezig. hilair geen duidelijke adenopathie. op de longvensters zijn enkele longnoduli zichtbaar, consistent met voorgaande onderzoeken en zonder toename in grootte; de grootste heeft een diameter van ongeveer 6 mm.
er zijn bekende hypervasculaire lesies in de lever, die in grootte zijn toegenomen. sommige van deze lesies tonen meer necrose. normale bevindingen van de milt, pancreas en galblaas. geen duidelijke adenopathie para-aorta, iliacaal en inguinaal. geen evidente afwijkingen aan het darmkanaal.
op de botvensters zijn geen focale skeletlesies waargenomen.
impressie:
progressie van de lesies.

Task 4: Skin histopathology case selection

Background: The frequency of basal cell carcinoma (BCC) cases is putting an increasing strain on dermatopathologists. BCC is the most common type of skin cancer, and its incidence is increasing rapidly worldwide. AI can play a significant role in reducing the time and effort required for BCC diagnostics and thus improve the overall efficiency of the process.

The diagnostic procedure for BCC biopsies involves two assessments by the pathologist: first, determining the presence of BCC, and second, identifying its subtype. BCC is categorized into four main subtypes: nodular, superficial, micronodular, and infiltrative. Correctly identifying these subtypes is needed, as each requires a specific surgical approach for effective treatment.

Given this context, a large dataset of pathology reports is an invaluable place to find and label cases. It serves as a foundational resource for training AI models to recognize and differentiate between BCC subtypes from biopsy samples. The process of gathering and labeling a significant volume of BCC cases involves extracting relevant information from the unstructured text of pathology reports. The challenge with unstructured text is its susceptibility to varied interpretations, making it difficult to understand the meaning of sentences using standard text filtering strategies like regex.

To overcome this hurdle, a method should be developed that enables accurate labeling and structuring of data within the reports. The effectiveness of the method is directly linked to the ability to accurately identify the presence of BCC: the higher the sensitivity, the more data can be extracted from archives, and the higher the specificity, the higher the quality of the data.

Task: predict whether the report matches any of the exclusion criteria (incomplete report or incomplete diagnosis). Provide your prediction as a confidence score between 0 and 1, where 0 corresponds to False (complete report) and 1 corresponds to True (incomplete report).

Dataset: 13183 cases (6555 patients) were selected from patients who underwent a skin cancer biopsy between 1 January 2003 and 31 December 2022 at Radboudumc with the diagnostic code for BCC. This dataset was constructed as part of the work by D.G.[1]. 756 cases (685 patients) were sampled and manually annotated by trained investigators (D.G. and J.S.B.). Reports were annotated at the report level as being "included" or "excluded".

Dataset splits: 30% of patients were randomly selected for the test set (225 cases) and the remaining 70% of patients (531 cases) were used for model development with 5-fold cross-validation.

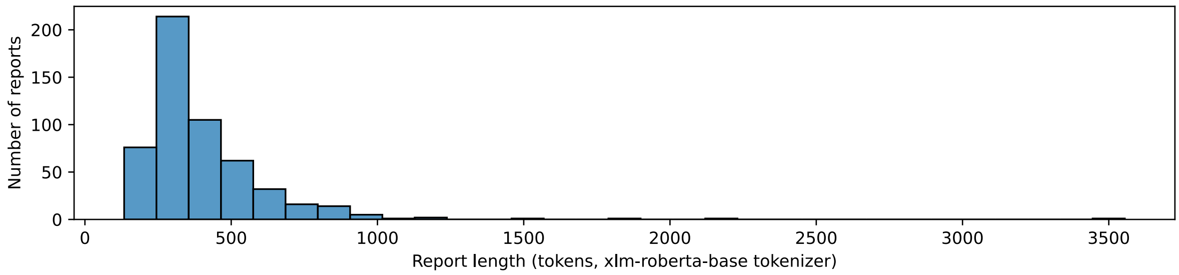

Supplementary Figure 5: Distribution of report lengths. The reports in the development dataset have a median length of 337 (95% CI: 196, 880) [min 134, max 3555] tokens with the xlm-roberta-base tokenizer.

Metric: The labels for this task are binary. The frequency of the labels is unbalanced. We use the Area Under the Receiver Operating Characteristic Curve (AUROC) to evaluate model performance.

Supplementary References

1. Geijs, D. J. *et al.* Detection and subtyping of basal cell carcinoma in whole-slide histopathology using weakly-supervised learning. *Medical Image Analysis* **93**, 103063 (2024).

Example

Label: True

Notes: the report is incomplete and does not present the diagnosis.

Anonymous sample report:
################################
DISCLAIMER:
THIS REPORT HAS BEEN ANONYMIZED BY REPLACING PATIENT HEALTH INFORMATION WITH RANDOM SURROGATES.
ANY RESEMBLANCE TO REAL PERSONS, LIVING OR DEAD, IS PURELY COINCIDENTAL.
################################

MACROSCOPIE:
8 06 Op formaline. I: huidstans met doorsnede van 0,4 cm en een diepte van 0,3 cm. Oppervlak korrelig beige. Onaangesneden t.i. 1b. Abev/Slev

HISTOLOGIE OPMERKING/GEWENST ONDERZOEK:
basaalcelcarcinoom? type?

UMCN IP PATH KLIN GEGEVENS:
op de rechter wang een 5mm grote glanzende huidskleurige papel met teleangectasieen

UMCN IP PATHO VERKRIJGINGSWIJZE HISTO:
biopt

UMCN IP HISTOLOGIE POTJE I:
3mm biopt rechter wang

Task 5: RECIST timeline

Background: In oncological CT, medical practitioners commonly use the Response Evaluation Criteria In Solid Tumors (RECIST) guidelines[1,2] to gauge the efficacy of treatments. This method involves identifying specific target lesions in the initial scan, evaluating their growth or shrinkage, and subsequently comparing these changes across multiple follow-up scans. However, complications arise when radiology reports are stored in an unstructured manner, impeding the identification of the baseline scan and the establishment of a chronological framework for subsequent scans.

Despite the inclusion of examination dates in reports, opting for the earliest date does not consistently yield the correct baseline scan. This discrepancy is attributable to the potential selection of new baselines following events such as the initiation of a new treatment or patient referral from a different treatment center. Consequently, a more nuanced approach is needed, entailing an examination of the report text to either confirm or deny its status as a baseline scan or through the absence of prior measurements for target lesions. Doing this manually proves impractical when confronted with a substantial volume of reports, essential for the construction of RECIST outcome trajectories across comprehensive patient populations over an extended time period.

Task: Predict on a report level whether this is a baseline or follow-up scan. Provide your prediction as a confidence score between 0 and 1, where 0 corresponds to False (follow-up report) and 1 corresponds to True (baseline report).

Dataset: 253 radiology reports of the Radboudumc and 144 reports of the Jeroen Bosch Ziekenhuis hospitals in the Netherlands were selected from archives containing thorax-abdomen CT scans conducted between 1 January 2000 and 31 December 2020. Reports were selected by filtering using regular expressions, searching for mention of ‘RECIST’, ‘target-’ or ‘indicator-lesions’. Patients with more than one such scan in the archive were included. Reports were manually annotated by a trained investigator (M.J.J.d.G.) under the supervision of a radiologist (E.Th.S.) to determine whether they were baseline or follow-up scans.

Dataset splits: 30% of patients were randomly selected for the test set (119 cases) and the remaining 70% of patients (278 cases) were used for model development with 5-fold cross-validation.

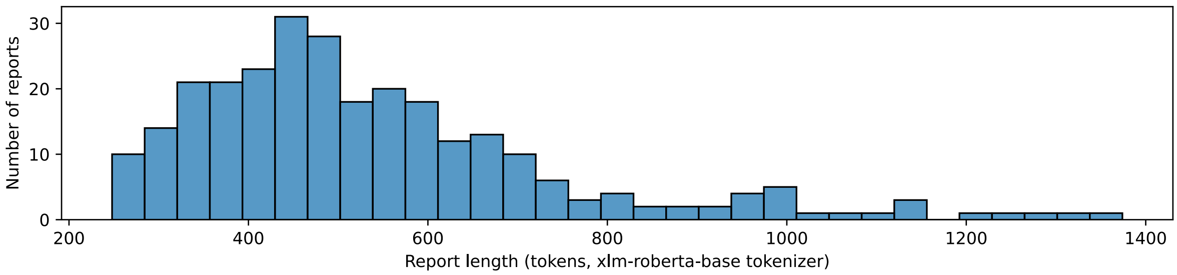

Supplementary Figure 6: Distribution of report lengths. The reports in the development dataset have a median length of 485 (95% CI: 268, 1127) [min 248, max 1374] tokens with the xlm-roberta-base tokenizer.

Metric: The labels for this task are binary. The frequency of the labels is slightly unbalanced. We use the Area Under the Receiver Operating Characteristic Curve (AUROC) to evaluate model performance.

Supplementary References

1. Eisenhauer, E. A. *et al.* New response evaluation criteria in solid tumours: Revised RECIST guideline (version 1.1). *European Journal of Cancer* **45**, 228–247 (2009).

2. Schwartz, L. H. *et al.* RECIST 1.1—Update and clarification: From the RECIST committee. *European Journal of Cancer* **62**, 132–137 (2016).

Example

Label: False

Notes: the report describes a follow-up report.

Anonymous sample report:
################################
DISCLAIMER:
THIS REPORT HAS BEEN ANONYMIZED BY REPLACING PATIENT HEALTH INFORMATION WITH RANDOM SURROGATES.
ANY RESEMBLANCE TO REAL PERSONS, LIVING OR DEAD, IS PURELY COINCIDENTAL.
################################

--Addendum start--
Target lesies: 12-06 Axillair links 68 x 40 mm (IMA 8-34) Axillair rechts 58 x 32 mm (IMA 3-38) Mediastinaal station 4R 27 x 14 mm (IMA 3-28) Precavaal 29 x 18 mm (IMA 8-104) Inguinaal links 36 x
22 mm (IMA 4-168) Inguinaal rechts 42 x 24 mm (IMA 4-172) SPD 5810 Non-target lesies: prominente
ring van Waldeyer, ongewijzigd craniocaudale afmeting van de milt bedraagt 11 cm Extranodaal: geen
Overige bevindingen: zie oorspronkelijk verslag
--Addendum einde--

Vraagstelling: Evaluatie tov CT 2 januari Evaluatie na 2 kuren chemotherapie

CT thorax abdomen, gescand na toediening van jodiumhoudend contrast intraveneus. Er is vergeleken met onderzoek van 5 maart en eerder.

Verslag:
CT-thorax en abdomen met intraveneus contrast. Ter correlatie de PET/CT van dezelfde datum. Ter vergelijking de CT van 5 maart 2020.

Thorax.
Beiderzijds axillair conglomeraat van pathologisch vergrote lymfklieren, met korte as diameter stand 35 mm rechts en 37 mm links. Tevens vergrote lymfklieren mediastinaal, twee van 14 mm op level 4 rechts. Tevens beiderzijds perihilair vergrote klieren en laag cervicaal enkele opvallende lymfklieren.
Gedilateerde atria, verwijde truncus pulmonalis en prominente aorta ascendens.

Coronaire sclerose. Focale gecalcificeerde atherosclerose in de aortaboog.
Scan in expiratie fase, waarbij matig beoordeelbaarheid van het longparenchym.
Bronchuswandverdikkingen en bronchiectasieen bij bronchopathie met versterkte interstitiele tekening en matglas.
Pulmonale laesies, met name rechts; subpleuraal 9 mm (9-101) en perifissuraal 6 mm (7-123) en 11 mm (11-159), meest waarschijnlijk lymfeklieren. Tevens ongewijzigde nodi van 5 mm (8-173), 7 mm (11-86) en 9 mm (11-189).
Inhomogene schildklier bij bekende calcificatie rechts, niet vergroot.

Abdomen.
Multipele opvallende lymfklieren abdominaal, waaronder in de leverhilus van 11 mm (3-86) en precavaal van 16 mm (8-104).
Multipele vergrote lymfklieren inguinaal en in de obturatorloge, met korte as diameter tot 19 mm links en de 24 mm rechts (4-168).
Bekende hyperperfusie in segment 6 van de lever. Slanke intrahepatische galwegen. Indruk van een heterogene lever, echter suboptimale scan fase ter beoordeling van de lever. Een en ander berust mogelijk nog op wisselende perfusie.
Bekend verwijde ductus choledochus, bij status na cholecystectomie?
Symmetrische aankleuring van de nieren. Bekende corticale intrekking rechts (9-193). Slank verzamelsysteem. Slanke bijnieren.
Normaal aspect van de milt. Geen splenomegalie.
Induratie van het mesenteriale vetweefsel.
Voorts normaal aspect van de tractus digestivus.
Atherosclerotische afwijkingen van de abdominale aorta en iliacaalvaten.
Normaal aspect van de blaas. Geen vrij vocht.

Skelet en weke delen.
Degeneratief aspect van het skelet. Geen ossale haarden.
Enkele intracutane noduli in de bekkenregio.

Impressie:

Multipele (sterk) vergrote lymfklieren verspreid over thorax en abdomen, met name beiderzijds axillair en inguinaal. Mogelijk in kader van progressie van ziekte bij chronische lymfatische leukemie.

Autorisatiedatum:
9 juni Contrastmiddel: Optiray 300: 100 cc

Task 6: Histopathology cancer origin

Background: The development of deep learning systems in the context of digital pathology requires large scale, retrospective collection of pathology slides from archives. Even when collection happens in a structured, case-specific fashion, it remains challenging to know for every individual pathology slide what kind of cancer it contains (if any) and what kind of tissue it is, as there may be many slides per case (originating from different biopsy locations, different slices within formalin-fixed, paraffin embedded blocks, et cetera). This information is only documented in unstructured pathology reports, which are difficult to process using rule-based systems and tedious to read manually.

Task: predict whether the cancer in this case originated in the lung, as opposed to a metastasis to the lung from a cancer in a different organ. Provide your prediction as a confidence score between 0 and 1, where 0 corresponds to False (metastasis from another organ) and 1 corresponds to True (originated in the lung).

Dataset: The patient population was selected based on patients suspected of having non-small cell lung cancer between 1 January 2016 and 31 December 2022 at Radboudumc. 1019 pathology reports were collected originating from 902 consecutive patients. The reports were read by trained student assistants and manually annotated for cancer origin (the cancer did/did not originate in the lung), sample type (the material is a biopsy, resection, or excision), and sample origin (the material was taken from the lungs/liver/brain/et cetera). Only the cancer origin labels are used for this task.

Dataset splits: 30% of patients were randomly selected for the test set (304 cases) and the remaining 70% of patients (715 cases) were used for model development with 5-fold cross-validation.

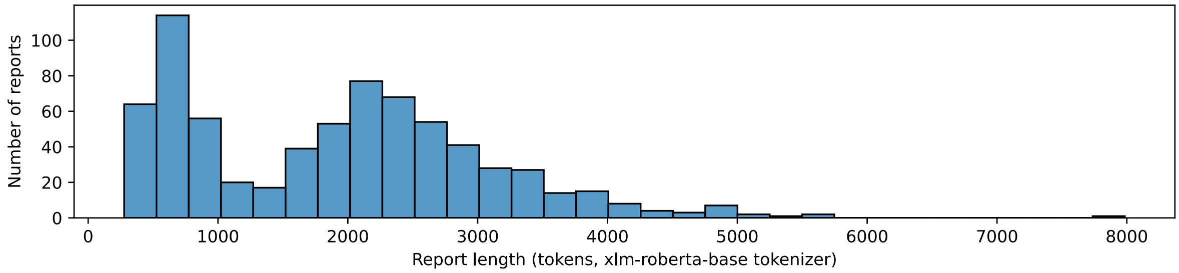

Supplementary Figure 7: Distribution of report lengths. The reports in the development dataset have a median length of 2001 (95% CI: 398, 4395) [min 277, max 7985] tokens with the xlm-roberta-base tokenizer.

Metric: The labels for this task are binary. The frequency of the labels is highly unbalanced. We use the Area Under the Receiver Operating Characteristic Curve (AUROC) to evaluate model performance.

Example

Label: True

Notes: the report describes that the cancer originates from the lung (“primaire origine in de long”).

Anonymous sample report:
################################
DISCLAIMER:
THIS REPORT HAS BEEN ANONYMIZED BY REPLACING PATIENT HEALTH INFORMATION WITH RANDOM SURROGATES.
ANY RESEMBLANCE TO REAL PERSONS, LIVING OR DEAD, IS PURELY COINCIDENTAL.

##############################

SAMENVATTING: Patholoog: Violet Marinus Datum ontvangst: 2 okt Datum antwoord : 4/10/2024 Aard materiaal : hersenen Klinische gegevens: Verkrijgingswijze resectie Klinische gegevens verdenking long metastase intracerebraal links parafalcien Vraagstelling aard van het weefsel. Longarts: G. Van Veen Bethesda Materiaal voor biobank Nee Inzending I tumor Ter informatie: dit verslag is elektronisch aangemaakt middels een spraakherkenningsysteem en elektronisch geautoriseerd. CONCLUSIE: excisie klinisch tumor, intracerebraal links parafalcien: beeld passend bij een metastase van een niet-kleincellig carcinoom, met immunohistochemisch aankleuringspatroon passend bij adenocarcinoom met primaire origine in de long. 4 okt 2024 AANVULLING (Lbu): PD-L1 immunohistochemisch positief (>50%). ALK rearrangement immunohistochemisch negatief. Voor DNA mutatieanalysen, zie T 24-817623. Voor fusiegenanalysen, zie T24-817631. MACROSCOPIE: 2 10 2024 Jaar I: vers ontvangen, fragment voor NEOt waarna op formaline. Macro volgt. Jbo 3/10/2024 In formaline. I: onregelmatig, elastisch, deels beige deels bruine biopt van maximaal 2,2 cm x 1,9 cm x 1,6 cm. Aan een zijde is het fragment afgekapseld met een dun vlies, waaronder een donkerblauwe verkleuring. In 4 lamellen. T.i. 3c. Hja MICROSCOPIE: hersenweefsel, met hierin een deels vitale tumor, opgebouwd uit in confluerende velden gelegen epithelioide cellen met overwegend ruim eosinofiel cytoplasma en vergrote soms sterk polymorfe kern, deels met prominente nucleoli. Hierbij op enkele plaatsen suggestie van buisvorming. Deze tumorcellen kleuren aan in de CK7 en TTF1 kleuring. 04.10 AANVULLING (Lbu); PD-L1 (22C3 DAKO) toont immunohistochemisch membraneuze, diffuse aankleuring in ca 80% van de tumorcellen. ALK-rearrangement immunohistochemisch negatief. Aanvullende bevindingen telefonisch medegedeeld aan behandelend logarts op 04.10, 13u45. ISO: Het laboratorium voor pathologie is geaccrediteerd volgens ISO 15189 RvA_TESTEN_M123. Voor de moleculaire analyses wordt deels gebruik gemaakt van faciliteiten van Genoomdiagnostiek Groningen onder ISO 15189 accreditatie M321. Moleculair microbiologisch onderzoek wordt uitbesteed aan Laboratorium Medische Microbiologie onder ISO 15189 accreditatie M456. Bij niet geaccrediteerde testen wordt dit expliciet vermeld.

Task 7: Pulmonary nodule size presence

Background: Outside a screening setting, early-stage lung cancer is generally detected after the detection of an incidental pulmonary nodule in a clinically ordered chest CT scan[1,2]. Trend analysis of the incidence of pulmonary nodules is important, as these statistics are essential in clinical decision-making and risk communication between physicians and patients with such a nodule[3]. However, identifying and analyzing nodules from unstructured radiology reports at scale is challenging. Automated detection of the presence of a size measurement – without necessarily extracting its exact value – can already be highly valuable. For example, confirming which reports contain nodule sizes can facilitate large-scale trend analyses (such as examining whether size reporting has increased over time) and efficient development of deep learning-based pulmonary nodule detection systems[4]. In addition, separating the task of determining whether a size is present from the task of extracting its exact value enables clearer and fairer evaluation metrics, ensuring that classification (size present vs. not present) and measurement extraction can be benchmarked independently.

Task: Predict whether the described pulmonary nodule in the radiology report has a size measurement. Provide your prediction as a confidence score between 0 and 1, where 0 corresponds to False (no size reported) and 1 corresponds to True (size reported).

Dataset: Hendrix et al.[1] created a dataset for nodule trend analysis with chest CT reports from two Dutch hospitals, acquired between 1 January 2008 and 31 December 2019 from Radboudumc and Jeroen Bosch Ziekenhuis. This dataset consists of a development set and an independent test set: the development set contains 1000 randomly sampled radiology reports (500 per hospital). The test set contains 200 randomly sampled reports (100 per hospital). Each report was given a label indicating (1) whether a pulmonary nodule was reported and (2) the diameter of the largest reported nodule if available (otherwise, a missing value was registered). All pulmonary nodules were included with a maximum diameter of 30 mm, regardless of morphology, type (i.e., solid, part-solid, non-solid, calcified, or perifissural), and malignancy status. For this task, all cases with a pulmonary nodule were selected.

Dataset splits: The test and development datasets were kept the same as in the study of Hendrix et al.[1]. This selected 66 cases for testing and 348 cases for development. We used the development dataset for 5-fold cross-validation.

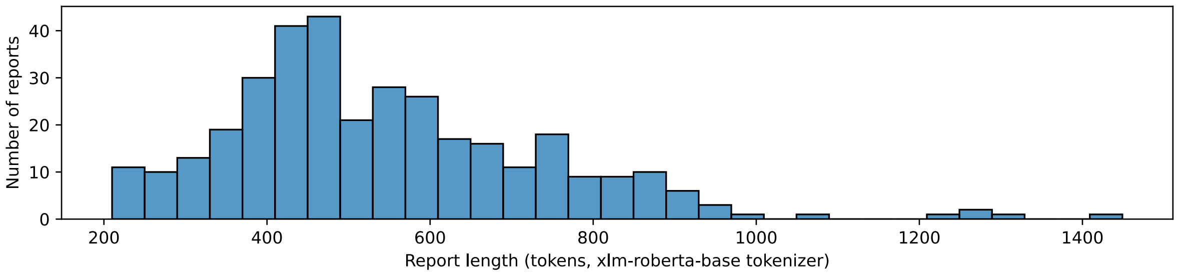

Supplementary Figure 8: Distribution of report lengths. The reports in the development dataset have a median length of 504 (95% CI: 241, 937) [min 210, max 1449] tokens with the xlm-roberta-base tokenizer.

Metric: The labels for this task are binary. The frequency of the labels is unbalanced. We use the Area Under the Receiver Operating Characteristic Curve (AUROC) to evaluate model performance.

Supplementary References

1. Hendrix, W. *et al.* Trends in the incidence of pulmonary nodules in chest computed tomography: 10-year results from two Dutch hospitals. *Eur Radiol* (2023) doi:10.1007/s00330-023-09826-3.

2. Gould, M. K. *et al.* Recent Trends in the Identification of Incidental Pulmonary Nodules. *American Journal of Respiratory and Critical Care Medicine* **192**, 1208–1214 (2015).
3. Wiener, R. S., Gould, M. K., Woloshin, S., Schwartz, L. M. & Clark, J. A. What Do You Mean, a Spot?: A Qualitative Analysis of Patients' Reactions to Discussions With Their Physicians About Pulmonary Nodules. *CHEST* **143**, 672–677 (2013).
4. Gu, Y. *et al.* A survey of computer-aided diagnosis of lung nodules from CT scans using deep learning. *Computers in Biology and Medicine* **137**, 104806 (2021).

Example

Label: True

Notes: the report describes a nodule of 7 mm ("7 millimeter grote nodus").

Anonymous sample report:
###############################
DISCLAIMER:
THIS REPORT HAS BEEN ANONYMIZED BY REPLACING PATIENT HEALTH INFORMATION WITH RANDOM SURROGATES.
ANY RESEMBLANCE TO REAL PERSONS, LIVING OR DEAD, IS PURELY COINCIDENTAL.
###############################

Relevante voorgeschiedenis: Status na resectie sarcoom rechts parailiacaal.
Status na recidief rechterlies (27-05) status na rectumamputatie en stoma
('05). Vraagstelling: pulmonale metastasen?

Verslag:
Ter hoogte van de linker onderkwab segment 6 is aan 7 millimeter grote
nodus zichtbaar met centrale calcificatie. (Serie 5 ima 456. Radiologisch
meest passend bij granuloom. Verder met name apicaal centrilobulair
emfysemateuze veranderingen . Enkele kleine subpleurale strengetjes zonder
verdere pathologie.
Mediastinaal normaal verloop van de grote vaten. Geringe
atherosclerotische veranderingen. Relatief uitgebreide coronaire
calcificaties en enkele verkalkingen in de mitralisklep ring. Mediastinaal
geen pathologisch vergrote klieren. Axillair geen bijzonderheden.
Voorzover afgebeeld in bovenbuik normaal aspect van parenchymateuze
organen behoudens een prominente cyste in de ventrale zijde van milt met
een doorsnede van 38 mm. Atherosclerotische veranderingen. Degeneratieve
veranderingen van afgebeelde ossale structuren zonder verdere pathologie.
In de subcutane weke delen hoog links dorsaal ter hoogte van C6 bevindt
zich een bolronde verdichting met een doorsnede van 30 mm.

Impressie:

Intrapulmonale nodus in de linker onderkwab met radiologische kenmerken
van granuloom.
Geen aanwijzingen voor metastasen.
Bolronde verdichting subcutaan in de weke delen ter hoogte van C6 links
paramediaan. Gaarne klinische informatie. Atheroomcyste?

Task 8: Pancreatic ductal adenocarcinoma size presence

Background: Pancreatic ductal adenocarcinoma (PDAC) is estimated to become the second leading cause of cancer-related deaths in Western countries by 2030[1]. Due to the lack of early, disease-specific symptoms, 80–85% of patients are diagnosed at advanced disease stages (stages III-IV). However, patients diagnosed at an early stage (stage I) present a significantly more favorable prognosis than stage IV patients (median survival: 26 vs. 4.8 months), making early detection the current most effective approach to improving outcomes[2]. Identifying whether a report includes a tumor size measurement is an important first step for large-scale dataset selection and management. This ensures that only relevant reports containing size data are flagged for further analysis or model training. By separating this classification task from the more complex extraction of exact measurements, we reduce ambiguity in performance metrics and enable more consistent evaluation of models designed for automated size presence detection versus those intended for accurate size quantification.

Task: predict whether the described PDAC in the radiology report has a size measurement. Provide your prediction as a confidence score between 0 and 1, where 0 corresponds to False (no size measurement) and 1 corresponds to True (size measurement provided).

Dataset: 2343 consecutive patients undergoing pancreatic and liver CECT scans between 1 January 2011 and 31 December 2022 at Radboud University Medical Center were included. Additionally, all CT scans for 1076 consecutive patients who underwent histopathology analysis of pancreas specimens or distant metastasis of pancreatic cancer between 1 January 2006 and 31 December 2022 at Radboud University Medical Center were retrospectively collected. This resulted in 3419 selected patients. For each patient, the baseline diagnostic scan was considered for positive cases and the most recent scan mentioning a normal pancreas was considered for negative cases. 121 patients were excluded for the absence of a baseline diagnostic report or no mention of the pancreas. This is the dataset used also in the PANORAMA study[3]. 2035 cases (2035 patients) were sampled and manually annotated by one of three trained investigators (N.A., M.Sch., I.M.E.S.). The 651 cases with a PDAC lesion were selected for this task. 54 cases were excluded for the absence of a radiology report. This included 597 cases (597 patients) for this task.

Dataset splits: 30% of patients were randomly selected for the test set (179 cases) and the remaining 70% of patients (418 cases) were used for model development with 5-fold cross-validation.

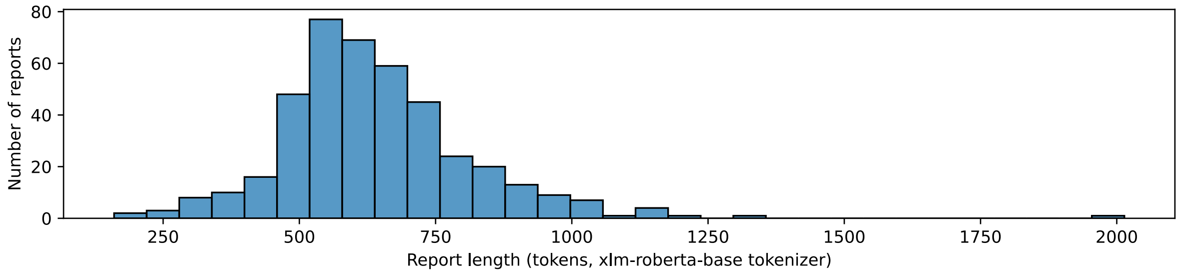

Supplementary Figure 9: Distribution of report lengths. The reports in the development dataset have a median length of 615 (95% CI: 324, 1021) [min 160, max 2014] tokens with the xlm-roberta-base tokenizer.

Metric: The labels for this task are binary. The frequency of the labels is highly unbalanced. We use the Area Under the Receiver Operating Characteristic curve (AUROC) to evaluate model performance.

Supplementary References

1. Ryan, D. P., Hong, T. S. & Bardeesy, N. Pancreatic Adenocarcinoma. *N Engl J Med* **371**, 1039–1049 (2014).
2. Schwartz, N. R. M. *et al.* Potential Cost-Effectiveness of Risk-Based Pancreatic Cancer Screening in Patients With New-Onset Diabetes. *Journal of the National Comprehensive Cancer Network* **20**, 451–459 (2022).
3. Alves, N. *et al. The PANORAMA Study Protocol: Pancreatic Cancer Diagnosis - Radiologists Meet AI*. https://zenodo.org/doi/10.5281/zenodo.10599559 (2024) doi:10.5281/ZENODO.10599559.

Example

Label: True

Notes: the report describes a pancreas lesion of 43 mm ("Hypovasculaire tumor in pancreascorpus (43 mm)").

Anonymous sample report:
################################
DISCLAIMER:
THIS REPORT HAS BEEN ANONYMIZED BY REPLACING PATIENT HEALTH INFORMATION WITH RANDOM SURROGATES.
ANY RESEMBLANCE TO REAL PERSONS, LIVING OR DEAD, IS PURELY COINCIDENTAL.
################################
Gerichte vraagstelling: metastase?
Verslag:
Herbeoordeling CT d.d. 20 maart 2006. Hypovasculaire tumor in pancreascorpus
en staart (43 mm) met occlusie vena lienalis; mesenteriale en
perigastrische collateralen; vervorming portomesenterisch confluens met
doorgroei langs VMS; volledige encasement truncus coeliacus en a. lienalis
met irregulaire contour; partiele encasement art. Hepatica communis;
doorgroei richting aorticorenale plexus rechts. Enkele kleine lymfklieren
craniaal van de truncus coeliacus (6 mm) Normale grootte van de lever, met
meerdere cysten. Hypodense lesie in leversegment 5 (32 mm, 204-236).
Degeneratief skelet. Dikke galwegen. Normaal aspect galblaas. Normaal
aspect bijnieren. Normale aankleuring en grootte van de nieren met
meerdere corticale cysten. Arteriosclerose grote retroperitoneale vaten.
Normaal aspect urineblaas. Normale grootte prostaat en zaadblazen.
Verkalkingen in vas deferens rechts. Diverticulosis colon sigmoideum.
Volumeverlies linker onderkwab. Geen pleuravocht en/of pericardvocht.
Conclusie.
Hypovasculaire tumor in pancreascorpus (43 mm) met doorgroei in
de mesenteriale wortel en encasement truncus coeliacus, a. hepatica
communis en art. Lienalis; doorgroei richting aorticorenale plexus rechts.
Waarschijnlijk metastase in leversegment 7 (32 mm).

Task 9: Pancreatic ductal adenocarcinoma diagnosis

Background: Pancreatic ductal adenocarcinoma (PDAC) is estimated to become the second leading cause of cancer-related deaths in Western countries by 2030[1]. Due to the lack of early, disease-specific symptoms, 80–85% of patients are diagnosed in advanced disease stages (stages III-IV). However, patients diagnosed at an early stage (stage I) present a significantly more favorable prognosis than stage IV patients (median survival: 26 vs. 4.8 months), making early detection the current most effective approach to improving outcomes[2]. Abdominal contrast-enhanced computed tomography (CECT) scans are usually the first line of diagnosis for PDAC, as there are no validated early diagnostic biomarkers. Automatic detection of PDAC on CECT scans is crucial to improving early detection on routine abdominal imaging. Artificial intelligence (AI) algorithms show great potential for this task but require large annotated data sets for model training. Since PDAC imaging can be done with multiple contrast CT protocols, the first step to curating large, representative data sets for model training is parsing radiology reports to extract the PDAC diagnosis, and information regarding the tumor (lesion attenuation and location). This is a straightforward task for trained investigators, but tedious to perform manually for thousands of reports.

Task: predict whether the radiologist describes pancreatic ductal adenocarcinoma (PDAC), other pancreatic disease, or a normal pancreas, as shown on the chest CT scan. Provide your prediction as a string, being “PDAC”, “Other pancreatic disease” or “Normal pancreas”.

Dataset: 2343 consecutive patients undergoing pancreatic and liver CECT scans between 1 January 2011 and 31 December 2022 at Radboud University Medical Center were included. Additionally, all CT scans for 1076 consecutive patients who underwent histopathology analysis of pancreas specimens or distant metastasis of pancreatic cancer between 1 January 2006 and 31 December 2022 at Radboud University Medical Center were retrospectively collected. This resulted in 3419 selected patients. For each patient, the baseline diagnostic scan was considered for positive cases and the most recent scan mentioning a normal pancreas was considered for negative cases. 121 patients were excluded for the absence of a baseline diagnostic report or no mention of the pancreas. This is the dataset used also in the PANORAMA study[3]. 2035 cases (2035 patients) were sampled and manually annotated by one of three trained investigators (N.A., M.Sch., I.M.E.S.). 73 cases were excluded for the absence of a radiology report. This included 1962 cases (1962 patients) for this task.

Dataset splits: 30% of patients were randomly selected for the test set (588 cases) and the remaining 70% of patients (1374 cases) were used for model development with 5-fold cross-validation.

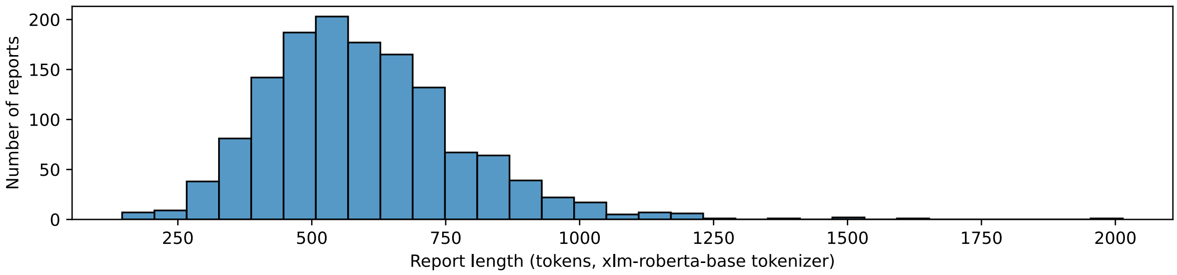

Supplementary Figure 10: Distribution of report lengths. The reports in the development dataset have a median length of 572 (95% CI: 303, 1010) [min 146, max 2014] tokens with the xlm-roberta-base tokenizer.

Metric: The labels for this task are categorical. The frequency of the labels is unbalanced, and the labels are not ordinal. Therefore, we use the Unweighted Kappa to evaluate model performance.

Supplementary References

1. Ryan, D. P., Hong, T. S. & Bardeesy, N. Pancreatic Adenocarcinoma. *N Engl J Med* **371**, 1039–1049 (2014).
2. Schwartz, N. R. M. *et al.* Potential Cost-Effectiveness of Risk-Based Pancreatic Cancer Screening in Patients With New-Onset Diabetes. *Journal of the National Comprehensive Cancer Network* **20**, 451–459 (2022).
3. Alves, N. *et al. The PANORAMA Study Protocol: Pancreatic Cancer Diagnosis - Radiologists Meet AI*. https://zenodo.org/doi/10.5281/zenodo.10599559 (2024) doi:10.5281/ZENODO.10599559.

Example

Label: PDAC

Notes: the report describes a pancreas tumor of 43 mm ("Hypovasculaire tumor in pancreascorpus (43 mm)").

Anonymous sample report:
################################
DISCLAIMER:
THIS REPORT HAS BEEN ANONYMIZED BY REPLACING PATIENT HEALTH INFORMATION WITH RANDOM SURROGATES.
ANY RESEMBLANCE TO REAL PERSONS, LIVING OR DEAD, IS PURELY COINCIDENTAL.
################################
Gerichte vraagstelling: metastase?
Verslag:
Herbeoordeling CT d.d. 20 maart 2006. Hypovasculaire tumor in pancreascorpus
en staart (43 mm) met occlusie vena lienalis; mesenteriale en
perigastrische collateralen; vervorming portomesenterisch confluens met
doorgroei langs VMS; volledige encasement truncus coeliacus en a. lienalis
met irregulaire contour; partiele encasement art. Hepatica communis;
doorgroei richting aorticorenale plexus rechts. Enkele kleine lymfklieren
craniaal van de truncus coeliacus (6 mm) Normale grootte van de lever, met
meerdere cysten. Hypodense lesie in leversegment 5 (32 mm, 204-236).
Degeneratief skelet. Dikke galwegen. Normaal aspect galblaas. Normaal
aspect bijnieren. Normale aankleuring en grootte van de nieren met
meerdere corticale cysten. Arteriosclerose grote retroperitoneale vaten.
Normaal aspect urineblaas. Normale grootte prostaat en zaadblazen.
Verkalkingen in vas deferens rechts. Diverticulosis colon sigmoideum.
Volumeverlies linker onderkwab. Geen pleuravocht en/of pericardvocht.
Conclusie.
Hypovasculaire tumor in pancreascorpus (43 mm) met doorgroei in
de mesenteriale wortel en encasement truncus coeliacus, a. hepatica
communis en art. Lienalis; doorgroei richting aorticorenale plexus rechts.
Waarschijnlijk metastase in leversegment 7 (32 mm).

Task 10: Prostate radiology suspicious lesions on prostate MRI

Background: Prostate cancer has 1.4 million new cases each year[1], a high incidence-to-mortality ratio and risks associated with treatment and biopsy; making non-invasive diagnosis of clinically significant prostate cancer (csPCa) crucial to reduce both unnecessary (confirmatory) biopsies and overtreatment[2]. Multiparametric MRI (mpMRI) scans interpreted by expert prostate radiologists provide the best non-invasive diagnosis[3]. These radiological findings are described using the Prostate Imaging – Reporting and Data System (PI-RADS)[4]. The PI-RADS scoring system assigns scores to describe the likelihood of a lesion being csPCa: 1 (very unlikely), 2 (unlikely), 3 (equivocal), 4 (likely), or 5 (very likely). Lesions that are not csPCa can be, for example, indolent cancer, prostatitis, or benign prostatic hyperplasia. Lesions with a PI-RADS score of 3, 4 or 5 are typically biopsied to confirm or rule-out the presence of csPCa. The number of PI-RADS 3 – 5 lesions is therefore clinically relevant, and can be used to train deep learning algorithms using Report-guided semi-supervised learning[5]. This is a straightforward task for trained investigators, but tedious to perform manually for thousands of reports.

Task: predict the number of PI-RADS 3 – 5 lesions based on the radiology report. Provide your prediction as a string, being "0", "1", "2", "3", or "4".

Dataset: This dataset is constructed from three sources:

1. Radboudumc. The PI-CAI Challenge[6] was hosted in 2022. The PI-CAI Public and Private Training dataset contains 9107 cases for which lesion-level scores and clinical parameters were manually annotated by one of six trained investigators (I.S., A.S., J.J.T., J.S.B., G.S., and E.M.G.T.) under supervision of a radiologist with 7 years of experience with prostate MRI (M.d.R.). For this task, we selected all cases with a radiology report from Radboudumc, selecting 6578 cases (5679 patients). Cases from Ziekenhuisgroep Twente were excluded to prevent overlap between the pretraining dataset and benchmark. Cases from University Medical Center Groningen were excluded because radiology reports were not available for the PI-CAI Public and Private Training dataset. 4 cases were excluded due to the report being English. This included 6574 cases (5676 patients).
2. University Medical Center Groningen. 416 consecutive cases (392 patients) were selected from patients who had a suspicion of prostate cancer (elevated PSA levels, and/or abnormal digital rectal examination findings, and/or lower urinary tract symptoms) between 1 January 2021 and 31 December 2022 at University Medical Center Groningen. 39 cases were excluded because of previous prostate cancer treatment (24), missing PI-RADS values in the report (14) or not being a detection study (1). This selected 377 cases (367 patients). Findings were manually annotated by trained investigators (C.R., S.J.F., and QY.v.L.), under supervision of a radiologist with 9 years of experience with prostate MRI (D.Y.).
3. Antoni van Leeuwenhoek Hospital. 9766 consecutive cases (7841 patients) were selected from patients who had a suspicion of prostate cancer (elevated PSA levels, and/or abnormal digital rectal examination findings, and/or lower urinary tract symptoms) between 1 January 2012 and 17 February 2023 at Antoni van Leeuwenhoek Ziekenhuis. 273 cases (269 patients) were randomly sampled. Additionally, 250 consecutive cases (250 patients) between 14 October 2015 and 3 March 2016 were selected. 9 cases were selected in both sets and only included once. This selected 514 cases (498 patients), which were manually annotated by a trained investigator (C.R.), under supervision of a radiologist with 9 years of experience with prostate MRI (D.Y.). 125 cases were excluded, due to not being a prostate MRI detection study (25), having no PI-RADS scores reported (75), or having ambiguous PI-RADS scores (25). This included 389 cases (385 patients) for this task.

Combining these data sources included 7340 cases (6428 patients).

Dataset splits: 30% of patients were randomly selected for the test set (2229 cases) and the remaining 70% of patients (5111 cases) were used for model development with 5-fold cross-validation.

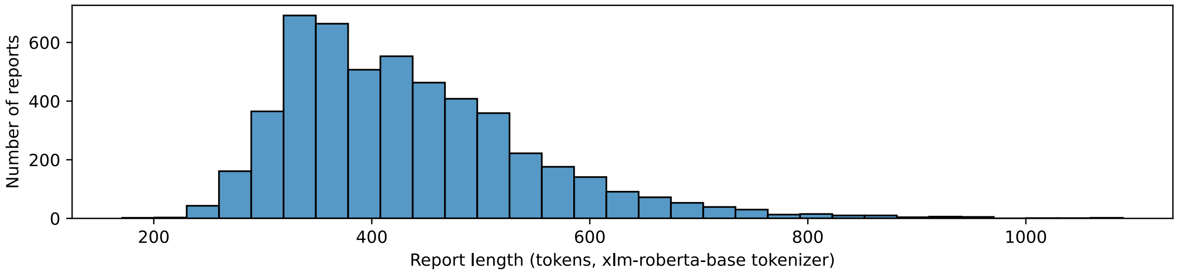

Supplementary Figure 11: Distribution of report lengths. The reports in the development dataset have a median length of 414 (95% CI: 275, 711) [min 171, max 1089] tokens with the xlm-roberta-base tokenizer.

Metric: The labels for this task range from 0 up to and including 4 and are ordinal. The frequency of the labels is highly imbalanced. Therefore, we use the Linearly Weighted Cohen's Kappa to evaluate model performance.

Supplementary References

1. Sung, H. *et al.* Global Cancer Statistics 2020: GLOBOCAN Estimates of Incidence and Mortality Worldwide for 36 Cancers in 185 Countries. *CA A Cancer J Clinicians* **71**, 209–249 (2021).
2. Stavrinides, V., Giganti, F., Emberton, M. & Moore, C. M. MRI in active surveillance: a critical review. *Prostate Cancer Prostatic Dis* **22**, 5–15 (2019).
3. Eldred-Evans, D. *et al.* Population-Based Prostate Cancer Screening With Magnetic Resonance Imaging or Ultrasonography: The IP1-PROSTAGRAM Study. *JAMA Oncol* **7**, 395 (2021).
4. Weinreb, J. C. *et al.* PI-RADS Prostate Imaging – Reporting and Data System: 2015, Version 2. *European Urology* **69**, 16–40 (2016).
5. Bosma, J. S. *et al.* Semisupervised Learning with Report-guided Pseudo Labels for Deep Learning–based Prostate Cancer Detection Using Biparametric MRI. *Radiology: Artificial Intelligence* **5**, e230031 (2023).
6. Saha, A. *et al.* Artificial intelligence and radiologists in prostate cancer detection on MRI (PI-CAI): an international, paired, non-inferiority, confirmatory study. *The Lancet Oncology* **25**, 879–887 (2024).

Example

Label: 1

Notes: The report describes a single PI-RADS 4.

Anonymous sample report:
##############################
DISCLAIMER:
THIS REPORT HAS BEEN ANONYMIZED BY REPLACING PATIENT HEALTH INFORMATION WITH RANDOM SURROGATES.
ANY RESEMBLANCE TO REAL PERSONS, LIVING OR DEAD, IS PURELY COINCIDENTAL.
##############################
Klinische gegevens:
PSA: 9,7; geen eerdere TRUS biopt(en); klinisch stadium: T 0 .
Verslag:
Er werd gescand met het detectie-protocol: locale prostaat T2WI (drie richtingen), DWI en DCE (Gadovist i.v.).
Bevindingen:
Index laesie mark1:
Plaats: rechts apicaal ventraal
Laesie grootte: 13 x 9 x 7mm.
T2W/DWI/DCE score: 4/4/-.
Minimale ADC waarde: 634 (normaal groter dan 950).
Risico categorie: Intermediate/high-grade Cancer (PIRADS v2 categorie: 4).
Onzekere extra-prostatische uitbreiding.
Impressie:
De prostaat heeft een volume van 68 cc. PSA densiteit: 0,14.
Afwijking passend bij significant carcinoom rechts ventraal apicaal (PIRADS 4). Onzekere extra-prostatische uitbreiding.

Task 11: Prostate histopathology significant cancers

Background: Prostate cancer has 1.4 million new cases each year, leading to over 300,000 cancer-related deaths each year[1]. Histopathological evaluation of biopsy or radical prostatectomy specimen provide conclusive evidence about lesions suspected of harboring clinically significant prostate cancer. These pathological findings are described using the Gleason grading system[2]. The Gleason grading system assigns scores to describe the features of the prostate tissue: 1 (well differentiated carcinoma), 2 (moderately differentiated carcinoma), 3 (moderately differentiated carcinoma), 4 (poorly differentiated carcinoma), or 5 (anaplastic carcinoma). Tissue with a pattern of 4 or 5 is considered clinically significant prostate cancer. Prostate tissue is typically assigned a primary and secondary grade, with the primary grade describing the dominant pattern (more than 50% of the tissue) and the secondary grade describing the next-most dominant pattern. The overall Gleason score is then represented as the primary score + secondary score (for example 4+3). Prostate tissue with a Gleason score sum of at least 7 is considered aggressive prostate cancer, and these scores are crucial to inform the patient treatment pathway. The number of Gleason score ≥ 7 lesions is therefore clinically relevant, and it can be used to train deep learning algorithms using Report-guided semi-supervised learning[3]. Determining this number is a straightforward task for trained investigators, but tedious to perform manually for thousands of reports.

Task: predict the number of Gleason score ≥ 7 (≥ 3 + 4) lesions described in the pathology report. Provide your prediction as a string, being "0", "1", "2", or "3".

Dataset: This dataset is sourced from three hospitals:

4. Radboudumc. The PI-CAI Challenge[4] was hosted in 2022. The PI-CAI Public and Private Training dataset contains 9107 cases for which lesion-level scores and clinical parameters were manually annotated by trained investigators (I.S., A.S., J.J.T., J.S.B., G.S., and E.M.G.T.) under supervision of a radiologist with 7 years of experience with prostate MRI (M.d.R.). For this task, we selected all cases with a pathology report from Radboudumc, selecting 2575 cases (2448 patients). Cases from Ziekenhuisgroep Twente were excluded to prevent overlap between the pretraining dataset and benchmark. Cases from University Medical Center Groningen were excluded because pathology reports were not available for the PI-CAI Public and Private Training dataset.
5. University Medical Center Groningen. 235 consecutive cases (228 patients) were selected from patients who had a suspicion of prostate cancer (elevated PSA levels, and/or abnormal digital rectal examination findings, and/or lower urinary tract symptoms) between 1 January 2021 and 31 December 2022 at University Medical Center Groningen, and subsequently received prostate histological evaluation. 8 cases were excluded because of previous prostate cancer treatment. This included 227 cases (220 patients). Findings were manually annotated by trained investigators (C.R., S.J.F., and QY.v.L.), under supervision of a radiologist with 9 years of experience with prostate MRI (D.Y.).
6. Antoni van Leeuwenhoek Ziekenhuis. 7581 consecutive cases (5017 patients) were selected from patients who received a prostate histopathological evaluation between 1 January 1995 and 17 February 2023 at Antoni van Leeuwenhoek Ziekenhuis. 462 cases (441 patients) were randomly sampled and manually annotated by a trained investigator (J.S.B.). 89 cases (76 patients) were excluded, because the report did not describe prostate histopathology (88) or because the Gleason score was invalid (1). This included 373 cases (366 patients).

Combining these data sources selected 3172 cases (3034 patients). From these, 7 cases with 4 or more ISUP ≥ 2 lesions were excluded, due to the sample size. This included 3165 cases (3027 patients) for this task.

Dataset splits: 30% of patients were randomly selected for the test set (952 cases) and the remaining 70% of patients (2213 cases) were used for model development with 5-fold cross-validation.

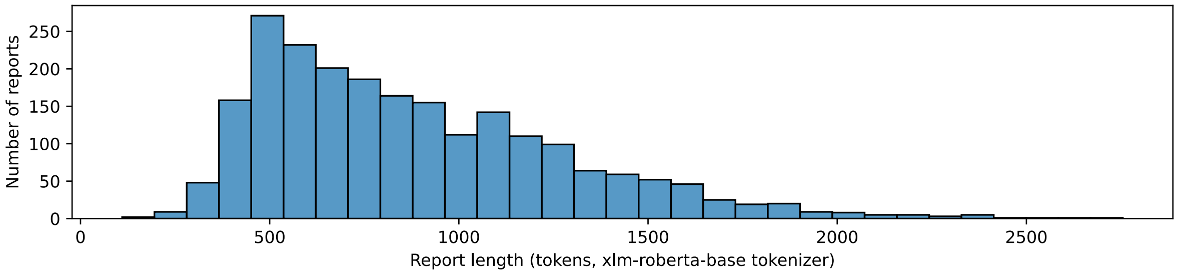

Supplementary Figure 12: Distribution of report lengths. The reports in the development dataset have a median length of 792 (95% CI: 364, 1836) [min 110, max 2755] tokens with the xlm-roberta-base tokenizer.

Metric: The labels for this task range from 0 up to and including 3 and are ordinal. The frequency of the labels is highly imbalanced. We use the Linearly Weighted Cohen's Kappa to evaluate model performance.

Supplementary References

1. Sung, H. *et al.* Global Cancer Statistics 2020: GLOBOCAN Estimates of Incidence and Mortality Worldwide for 36 Cancers in 185 Countries. *CA A Cancer J Clinicians* **71**, 209–249 (2021).
2. Epstein, J. I., Egevad, L., Srigley, J. R. & Humphrey, P. A. The 2014 International Society of Urological Pathology (ISUP) Consensus Conference on Gleason Grading of Prostatic Carcinoma. *Am J Surg Pathol* **40**, (2016).
3. Bosma, J. S. *et al.* Semisupervised Learning with Report-guided Pseudo Labels for Deep Learning–based Prostate Cancer Detection Using Biparametric MRI. *Radiology: Artificial Intelligence* **5**, e230031 (2023).
4. Saha, A. *et al.* Artificial intelligence and radiologists in prostate cancer detection on MRI (PI-CAI): an international, paired, non-inferiority, confirmatory study. *The Lancet Oncology* **25**, 879–887 (2024).

Example

Label: 2

Notes: the report describes (1) a lesion in the PI-RADS 5 lesion in the left peripheral zone continuing in the medial transition zone with Gleason score 3+4, (2) a lesion in the PI-RADS 4 lesion in the right transition zone with Gleason score 3+4, (3) a lesion in the random left biopsy with Gleason score 3+4. The lesions (1) and (3) likely come from the same lesion, given the location and size of the PI-RADS 5 lesion (19 x 11 mm).

Anonymous sample report:
################################
DISCLAIMER:
THIS REPORT HAS BEEN ANONYMIZED BY REPLACING PATIENT HEALTH INFORMATION WITH RANDOM SURROGATES.
ANY RESEMBLANCE TO REAL PERSONS, LIVING OR DEAD, IS PURELY COINCIDENTAL.
################################

Klinische Gegevens:

P.S.A.: 7,5. Volume: 18 cc. M.R.I.: Pirads 5: Li. PZ doorlopend tot medial TZ: 19 11 mm. Pirads 4: TZ rechts, midprostaat: 9 x 4 mm. Vraagstelling: maligniteit? Omschrijving materiaal: prostaatbiopten. I-III: Pirads 5. IV-V: Pirads 4. VI-VIII: Random Re. IX-X: Random Li. Ter informatie: dit verslag is elektronisch aangemaakt middels een spraakherkenningsysteem en elektronisch geautoriseerd.

Macroscope:
24 december. I t/m X op formaline. I: pijpvormig weefselfragment 14 mm. T.i. 1b. II: pijpvormig weefselfragment 15 mm. Ingesloten bij I. III: pijpvormig weefselfragment 9 mm. T.i. 1b. IV: Pijpvormig weefselfragment 11 mm. T.i. 1b. V: pijpvormig weefselfragment 12 mm. Ingesloten bij IV. VI: pijpvormig weefselfragment van 8 mm. T.i. 1b. VII: pijpvormig weefselfragment van 11 mm. Ingesloten bij VI. VIII: pijpvormig weefselfragment van 13 mm. T.i. 1b. IX: pijpvormig weefselfragment van 14 mm. T.i. 1b. X: pijpvormig weefselfragment van 16 mm ingesloten bij IX. Alles aangekleurd eosine en ingepakt een papiertje. Linda Koe.

Microscope:
24 december. I en II tweetal pijpvormige prostaatweefselbiopten bestaande uit fibromusculair stroma, enkele preexsistente niet atypische klierbuizen alsmede diffuus infiltrerende atypische klierbuizen bekleed met slechts een enkelvoudige laag van kubocilindrisch epitheel waarin licht vergrote kernen met prominente nucleoli. In 1 biopt ook een focus van cribriforme groei. Cytoplasma deels wat basofiel. Uitbreiding over traject van 11 mm. III pijpvormig prostaatweefselbiopten bestaande uit fibromusculair stroma, organoid gelegen preexsistente klierbuizen alsmede deels nog dicht bij elkaar gelegen maar focaal ook infiltratief groeiende individuele buizen dit met eenlagig cilindrisch epitheel met atypische kernen en basofiel cytoplasma zoals in I/II beschreven, over een traject van 4 mm. IV en V een tweetal pijpvormige prostaatweefselbiopten met fibromusculair stroma, enkele preexsistente niet atypische klierbuizen alsmede diffuus infiltrerende atypische klierbuizen bekleed met eenlagig kubocilindrisch epitheel waarin vergrote kernen en prominente nucleoli. Overwegend acinair groeipatroon maar focaal ook fuserend. Dit over een traject van 9 mm. VI en VII tweetal pijpvormige prostaatweefselbiopten met fibromusculair stroma en preexsistente, organoid gelegen klierbuisjes bekleed met niet atypisch tweelagig epitheel. Focaal gemengdcellige, voornamelijk lymfocytaire ontsteking. In een groepje buizen enige kernstapeling en kernhyperchromasie, echter geen prominente nucleoli: onvoldoende kenmerken van high grade PIN. VIII een pijpvormig prostaatweefselbiopten met fibromusculair stroma en preexsistente, organoid gelegen klierbuisjes. Op een plaats uitgebreide gemengdcellige ontsteking neutrofiele component; basaalcel hyperplasie in de aangetaste klierbuizen. Geen atypie. IX en X een tweetal pijpvormige prostaatweefselbiopten met fibromusculair stroma en meer existente, organoid gelegen klierbuizen waarin focaal basaalcelhyperplasie/urotheliale metaplasie. In een biopt ook aantal diffuus infiltrerende klierbuizen bekleed met eenlagig cilindrisch epitheel waarin vergrote kernen met prominente nucleoli. Ook hier overwegend acinaire, deels fuserende groei. Over een traject van 3 mm.

Conclusion:
I t/m III PIRADS 5: in alle drie biopten adenocarcinoom, Gleason score 3 +4 is 7, totale tumorlengte 14 mm. IV en V PIRADS 4: in beide biopten adenocarcinoom, Gleason score 3 +4 = 7, totale tumorlengte 9 mm. VI t/m VIII random rechts: chronische actieve ontsteking, geen maligniteit. IX en X random links: in een van de twee biopten adenocarcinoom, Gleason score 3 +4 = 7, tumorlengte 7,5 mm. Prognostische score volgens Epstein: groep 2.

Diagnosis:
prostaat*rechts*links*biopt*adenocarcinoom

Task 12: Histopathology tissue type

Background: The development of deep learning systems in the context of digital pathology requires large scale, retrospective collection of pathology slides from archives. Even when collection happens in a structured, case-specific fashion, it remains challenging to know for every individual pathology slide what kind of cancer it contains (if any) and what kind of tissue it is, as there may be many slides per case (originating from different biopsy locations, different slices within formalin-fixed, paraffin embedded blocks, et cetera). This information is only documented in unstructured pathology reports, which are difficult to process using rule-based systems and tedious to read manually.

Task: predict whether the material is a biopsy, resection, or excision. Provide your prediction as a string, being “Biopsy”, “Resection”, or “Excision”.

Dataset: The patient population was selected based on patients suspected of having non-small cell lung cancer between 1 January 2016 and 31 December 2022 at Radboudumc. 1019 pathology reports were collected originating from 902 consecutive patients. The reports were read by trained student assistants and manually annotated for cancer origin (the cancer did/did not originate in the lung), sample type (the material is a biopsy, resection, or excision), and sample origin (the material was taken from the lungs/liver/brain/et cetera). Only the sample type label was used for this task. 8 cases were excluded for having an undeterminable sample type (4), being a frozen section (2), or cytology (2). This included 1011 cases (898 patients).

Dataset splits: 30% of patients were randomly selected for the test set (304 cases) and the remaining 70% of patients (707 cases) were used for model development with 5-fold cross-validation.

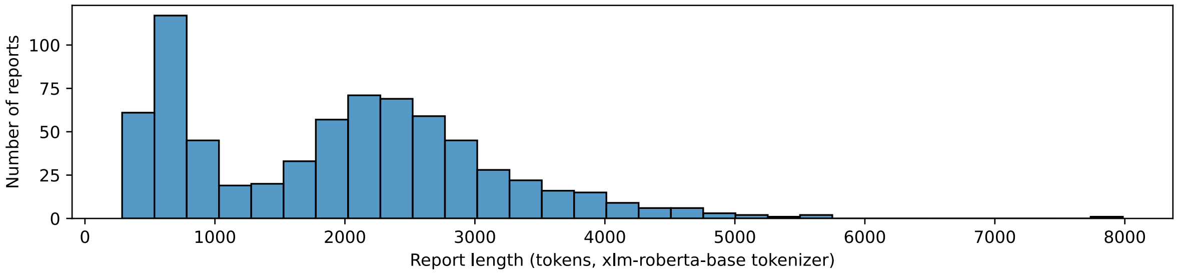

Supplementary Figure 13: Distribution of report lengths. The reports in the development dataset have a median length of 2025 (95% CI: 405, 4404) [min 286, max 7985] tokens with the xlm-roberta-base tokenizer.

Metric: The labels for this task are categorical. The frequency of the labels is unbalanced. The labels are not ordinal. Therefore, we use the Unweighted Kappa to evaluate model performance.

Example

Label: Biopsy

Notes: the report indicates the tissue is from a biopsy (“Verkrijgingswijze biopt”).

Anonymous sample report:
################################
DISCLAIMER:
THIS REPORT HAS BEEN ANONYMIZED BY REPLACING PATIENT HEALTH INFORMATION WITH RANDOM SURROGATES.
ANY RESEMBLANCE TO REAL PERSONS, LIVING OR DEAD, IS PURELY COINCIDENTAL.
################################

SAMENVATTING: Patholoog: PPan Datum ontvangst: 01/04/2019 Datum antwoord : 09 apr 2019 Aard materiaal : bot Klinische gegevens: Materiaal voor biobank->No Verkrijgingswijze biopt Klinische gegevens maligniteit bronchus Vraagstelling aard? metastase? Inzending I 2,5 g 16 x 16 mm biopt manubrium sterni Ter informatie: dit verslag is elektronisch aangemaakt middels een spraakherkenningsysteem en elektronisch geautoriseerd. CONCLUSIE: Botbiopten manubrium sterni : subcorticaal botweefsel gedeeltelijk geinfiltreerd door een matig gedifferentieerd adenocarcinoom. Immunoprofiel past bij een primair longcarcinoom (TTF-1 +). PD-L1 kleuring streng genomen als niet repraesentatief te beschouwen bij < 100 tumorcellen (3 van 86 tumorcellen positief). AANVULLING 3 april: helaas te weinig tumorcellen voor mutatieanalyse. MACROSCOPIE: 1 04 2019 Vers ontvangen. I: 2 botharde fragmenten. Een fragment van 2,5 mm en een fragment van 1,0 x 0,2 cm. Van het tweede fragment fragment afgenomen voor vries. Overige fragmenten ingesloten in cassette 1 deze in EDTA ontkalking. T.i. 1b. PPan MICROSCOPIE: 2 aprl. Het volledig ingesloten materiaal toont 3 grotere en meerdere zeer kleine biopten (de laatste voornamelijk bestaande uit necrotische botfragmentjes). In de grotere biopten corticale en spongieuze botbalkjes met focaal verhoogde activiteit van osteoclasten. In de mergholte oedemateus en deels desmoplastisch veranderd stroma, geen hematopoiese. Op meerdere plaatsen buisformatie is van atypische kubocilindrische epitheelcellen met hoge kerncytoplasmaverhouding en kernpolymorfie. Immunohistochemie: Tumorcellen positief met CK7 en TTF-1. CK7 is negatief. PD-L1 (E1L3N, Cell Signalling) toont immunohistochemisch membraneuze aankleuring in 3 van 86 tumorcellen. 4-04 AANVULLING. N.a.v. verzoek van behandelend skeletarts om EGFR mutatieanalyse zijn de preparaten gereviseerd. Er zijn minder dan 200 tumorcellen aanwezig hetgeen te weinig is voor mutatieanalyse.

Task 13: Histopathology tissue origin

Background: The development of deep learning systems in the context of digital pathology requires large scale, retrospective collection of pathology slides from archives. Even when collection happens in a structured, case-specific fashion, it remains challenging to know for every individual pathology slide what kind of cancer it contains (if any) and what kind of tissue it is, as there may be many slides per case (originating from different biopsy locations, different slices within formalin-fixed, paraffin embedded blocks, et cetera). This information is only documented in unstructured pathology reports, which are difficult to process using rule-based systems and tedious to read manually.

Task: predict from which organ the material was taken (lung, lymph node, bronchus, liver, brain, bone, or other). Provide your prediction as a string, being “lung”, “lymph node”, “bronchus”, “liver”, “brain”, “bone”, or “other”.

Dataset: The patient population was selected based on patients suspected of having non-small cell lung cancer between 1 January 2016 and 31 December 2022 at Radboudumc. 1019 pathology reports were collected originating from 902 consecutive patients. The reports were read by trained student assistants and manually annotated for cancer origin (the cancer did/did not originate in the lung), sample type (the material is a biopsy, resection, or excision), and sample origin (the material was taken from the lungs/liver/brain/et cetera). Only the sample origin label was used for this task. 4 cases were excluded because the report described samples from different origins. This included 1015 cases (899 patients).

Dataset splits: 30% of patients were randomly selected for the test set (297 cases) and the remaining 70% of patients (718 cases) were used for model development with 5-fold cross-validation.

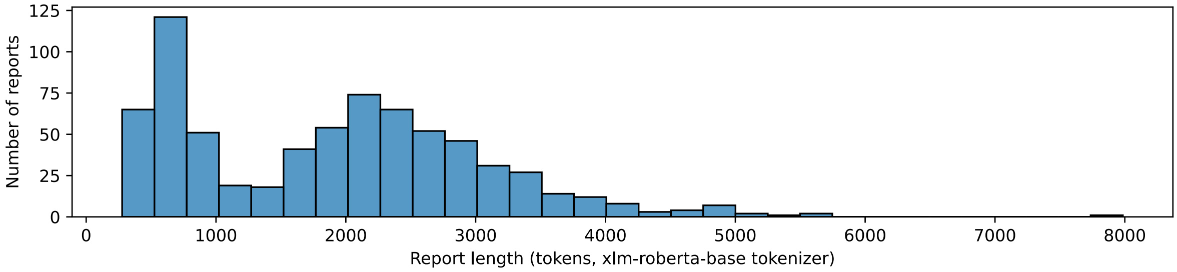

Supplementary Figure 14: Distribution of report lengths. The reports in the development dataset have a median length of 1992 (95% CI: 400, 4396) [min 277, max 7985] tokens with the xlm-roberta-base tokenizer.

Metric: The labels for this task are categorical. The frequency of the labels is unbalanced. The labels are not ordinal. Therefore, we use the Unweighted Kappa to evaluate model performance.

Example

Label: lung

Notes: the tissue originates from the lungs ("Aard materiaal : long").

Anonymous sample report:
################################
DISCLAIMER:
THIS REPORT HAS BEEN ANONYMIZED BY REPLACING PATIENT HEALTH INFORMATION WITH RANDOM SURROGATES.
ANY RESEMBLANCE TO REAL PERSONS, LIVING OR DEAD, IS PURELY COINCIDENTAL.

##############################
SAMENVATTING: Patholoog: Luigi Cascarino Datum ontvangst: 3 juli Datum antwoord : 4 juli Aard materiaal : long Klinische gegevens: Klinische gegevens Klinische gegevens:-; Indicatie aanvraag: progressieve verdichting LOK dorsaal tegen pleura aan. 2009-2010 urotheelcelcarcinoom operatie; Opmerking van radioloog: opmerkingen radioloog: meta? prim. RIP? Infiltraat? Anderszins? Slechts 1 biopt, hierna pneu.svp pat. op tijd ivm bespreking om 12:45. Armond; Datum onderzoek: 2 juli; Tijd onderzoek: : Inzending I materiaal: Long; locatie: rechts; verkrijgingswijze: biopt; zijdigheid: Rechts; formaline: Ja; Ter informatie: dit verslag is elektronisch aangemaakt middels een spraakherkenningsysteem en elektronisch geautoriseerd. CONCLUSIE: Biopt rechter onderkwab dorsaal tegen pleura: lokalisatie van ongedifferentieerd carcinoom. Immuunhistochemie volgt voor verdere subtypering. 5 juli. IHC beeld past niet bij een primair longadenocarcinoom (TTF1-) of plaveiselcelcarcinoom; het beeld kan passen bij bepaalde subtypes urotheelcelcarcinomen, maar het beeld komt niet helemaal overeen met de uretertumor uit 2010. p53/CDKN2A mutatie-analyse staat in voor onderzoek op verwantschap (primair-meta). 5 juli. De tumoren in T12-09539 (long) en Toorspronkelijk10-17282 (ureter) hebben een verschillende mutatiepatroon in TP53 en NRAS. Beide tumoren zijn derhalve naar alle waarschijnlijkheid afkomstig van verschillende primaire tumoren. AANVULLING 7 juli (I.V.): door behandelend arts werd TSO500 analyse aangevraagd voor identificatie van additionele driver mutaties/amplificaties. Echter, gezien de aanwezigheid van de NRAS driver mutatie is de verwachting van de Klinisch Moleculair Bioloog in Pathologie (KMBP) dat er geen additionele therapie targets aangetoond zullen worden. Deze analyse wordt daarom niet uitgevoerd. AANVULLING 8 juli (Armond) PD-L1 immunohistochemisch positief (>1%, \<50%). MACROSCOPIE: 9 juli. Op formaline: een grijs beige naaldbiopt met lengte van 17 mm. T.i. 1b. Peter. MICROSCOPIE: 10 juli. Naaldbiopt met een solide aspect, vrijwel geheel ingenomen door tumor. Aan randen nog wat alveolair weefsel. Uitgebreide desmoplastische stromareactie waarin individueel en in groepen gelegen zeer sterk atypische cellen met grote hyperchrome kernen, polymorfie soms wat spoelvormig cellaag, soms meer klierbuizen of carcinoom veld vormend en gelijkend op een UC. Necrose. In vergelijking met T 10-17282, tumor ureter, is de polymorfie sterker, hoewel ook in deze primaire tumor plaatselijk forse atypie wordt gezien. Immuunhistochemie volgt. 9 juli. De tumorcellen zijn positief voor ck5,6,14,7 en p53, negatief voor ck20,p63,GATA3, calretinin en d2-40, en TTF1. Hoewel deels overeenkomend is dit patroon ook deels anders dan de uretertumor (T10-17282); Ck5,6,7, en Ck20 en GATA 3 en p63+. Ook hier p53+. p16 (CDKN2A)+, E-cadherin + Negatief : ER, PR, CA19-9. WT1, pax8, pCEA. (Tr. genitalis niet waarschijnlijk) p53 en CDKN2A mutatie-analyse staat in op onderzoek primair, metastase. 10 juli Laboratorium Tumorgenetica onderzoek T11-185147: Bepaling clonale verwantschap met TP53 mutatie-analyse. RESULTAAT EN MOLECULAIRE INTERPRETATIE Analyses zijn uitgevoerd op DNA geisoleerd uit paraffine weefsel: T 11-627548 I1, long met 70% neoplastische cellen (gecontroleerd door EBus): DPA11-02376 T11-857908 I5, ureter met 70% neoplastische cellen (gecontroleerd door EBus): DPA11-02376 Single molecule Molecular Inversion Probe (smMIP) gebaseerde sequentie-analyse van het 'Radboud Cancer Hotspot genenpanel' (versie 1.0) en het 'TP53/CDKN2A genenpanel' (versie 1.0) op een NextSeq 500, waarbij mutaties met een analytische sensitiviteit van 1% kunnen worden aangetoond. Voor dit monster geldt dat de kans gemiddeld kleiner is dan 5% dat mutaties met een allelfrequentie >20% zijn gemist voor de onderstaande genen. EGFR (NM_005228.3) codon 465-499, 688-823, 849-875: geen mutatie KRAS (NM_004985.4) codon 12, 13, 59, 61, 117 en 146: mutatie c.34_35delinsTT (p.(Gly12Phe)); 15% mutant allel TP53 (NM_000546.5) >91% van het coderende gebied: mutatie c.818G>A (p.(Arg273His)); 35% mutant allel. CONCLUSIE De tumoren in T12-09539 (long) en T10-17282 (ureter) hebben een verschillende mutatiepatroon in TP53 en NRAS. Beide tumoren zijn derhalve naar alle waarschijnlijkheid afkomstig van verschillende primaire tumoren. TOELICHTING Met het 'Radboud Cancer Hotspot genenpanel' (versie 1.0) worden de volgende gebieden geanalyseerd: AKT1 (NM_005163.2): codon 17, BRAF (NM_004333.4): codon 582-615, CTNNB1 (NM_001904.3): codon 19-48, CXCR4 (NM_001008540.1): codon 281-357, EGFR (NM_005228.3): codon 434-499, 688-823, 849-875, ERBB2 (NM_004448.3): codon 770-785, EZH2 (NM_004456.4): codon 471-502, 618-645,

679-704, GNA11 (NM_002067.4): codon 183 en 209, GNAQ (NM_002072.3): codon 183 en 209, GNAS (NM_000516.4): codon 201 en 227, H3F3A (NM_002107.4): codon 28 en 35, H3F3B (NM_005324.4): codon 37, HRAS (NM_005343.2): codon 12, 13, 59 en 61, IDH1 (NM_005896.3): codon 132, IDH2 (NM_002168.3): codon 140 en 172, JAK2 (NM_004972.3): codon 617, KIT (NM_000222.2): codon 412-513, 550-591, 628-713, 799-828, KRAS (NM_004985.4): codon 12, 13, 59, 61, 117 en 146, MPL (NM_005373.2): codon 515, MYD88 (NM_002468.4): codon 169-280, NRAS (NM_002524.4): codon 12, 13, 59, 61, 117 en 146, PDGFRA (NM_006206.4): codon 552-596, 632-667, 814-848, PIK3CA (NM_006218.2): codon 520-554, 1020-1069, SF3B1 (NM_012433.2): codon 603-471, 833-906 en de markers BAT25, BAT26, NR21, NR24 en NR27 ten behoeve van microsatelliet instabiliteit (MSI) bepaling. Voor deze indicatie worden de relevante bevindingen in EGFR en KRAS gerapporteerd, aangevuld met gedetecteerde mutaties die voor andere vraagstellingen klinisch relevant zijn. Geautoriseerd door LGod 12 juli AANVULLLING (EBus): PD-L1 (E1L3N, Cell Signalling) toont immunohistochemisch membraneuze,pleksgewijze aankleuring in ca 30% van de tumorcellen.

Task 14: Entailment diagnostic sentences

Background: Textual entailment is a natural language processing task that involves determining the relationship between two pieces of text: a *premise* and a *hypothesis*. It aims to determine if the given texts are unrelated, contradictory or imply the same thing. It is used to create models that are able to judge semantic similarity between two texts.

The original MedNLI dataset[1] is a medical natural language inference dataset annotated by doctors, grounded in the medical history of patients. It is based on data from the MIMIC-III dataset[2]. The textual entailment dataset used in the DRAGON challenge is the MedNLI dataset translated to Dutch[3].

Task: Classify whether two sentences are semantically similar, contradictory, or neutral. Provide your prediction as a string, being "entailment", "contradiction", or "neutral".

Dataset: The original training dataset (11,222 sentence pairs) and validation dataset (1395 sentence pairs) are combined into the development dataset (12617 sentence pairs). The development dataset is used for model development with 5-fold cross-validation. The original test dataset (1422 sentence pairs) is used as-is for testing. Each example consists of two sentences and a label (entailment/contradiction/neutral).

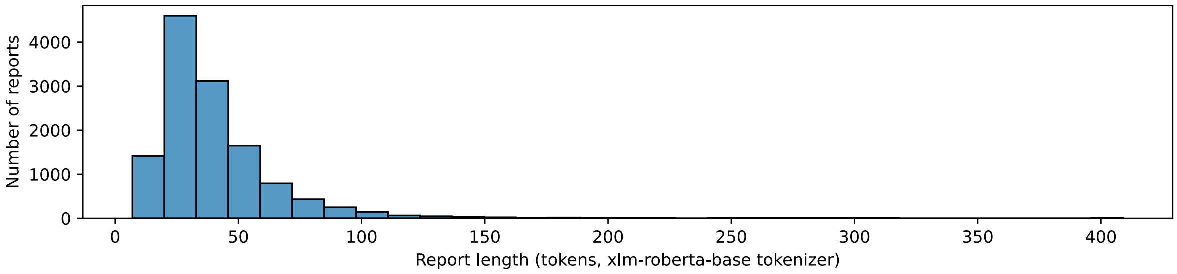

Supplementary Figure 15: Distribution of report lengths. The reports in the development dataset have a median length of 33 (95% CI: 14, 102) [min 7, max 409] tokens with the xlm-roberta-base tokenizer.

Metric: The labels for this task are entailment, neutral and contradiction. The frequency of the labels is balanced. The labels are ordinal, with "neutral" in between "entailment" and "contradiction". Therefore, we use the Linearly Weighted Cohen's Kappa metric to evaluate model performance.

Supplementary References

1. Romanov, A. & Shivade, C. Lessons from Natural Language Inference in the Clinical Domain. Preprint at http://arxiv.org/abs/1808.06752 (2018).
2. Johnson, A. E. W. *et al.* MIMIC-III, a freely accessible critical care database. *Sci Data* **3**, 160035 (2016).
3. Van Leijenhorst, L., De Vries, A. P., Habben Jansen, T. & Wertheim, H. SOPalign: A Tool for Automatic Estimation of Compliance with Medical Guidelines. in *Advances in Information Retrieval* (eds. Kamps, J. et al.) vol. 13982 307–312 (Springer Nature Switzerland, Cham, 2023).

Example

Label: contradiction

Notes: the two provided sentences contradict.

Anonymous sample report:

[
    "CAD, status na angioplastiek met stent twee jaar geleden.",
    "Geen voorgeschiedenis van hartziekte"
]

Task 15: Colon histopathology diagnosis

Background: Colorectal cancer (CRC) causes over 140,000 deaths annually in the US, ranking as the second leading cancer fatality. The standard diagnostic procedure for CRC is colonoscopy, examining the entire colon and rectum to detect abnormal tissue growth early.
During colonoscopy, precancerous polyps are removed, and biopsies may be taken for microscopic analysis. The primary objective is to identify adenocarcinomas, constituting approximately 96 percent of all CRC cases; additionally, polyps are categorized and graded for dysplasia.
The increasing number of patients due to cancer screening programs has significantly increased pathologist workloads, and biopsy visual inspection is time-consuming and susceptible to inter-observer variability. Computer-aided diagnosis using artificial intelligence applied to digital pathology whole-slide images can enhance histological analysis, reducing time, effort, and subjective bias. Specifically, pre-screening tissue samples to automatically exclude normal or benign cases would greatly assist histopathological analysis of polyps and colon biopsies, alleviating the pathologist's workload. To build AI-based systems at scale, to classify polyps and biopsies into all clinically relevant categories, the main clinical categories reported in the clinical report need to be extracted automatically from large corpora of pathology reports, to be used as weak labels for training of AI models.

Task: for the specified block, predict whether the specimen was obtained from 1) biopsy or polypectomy, and whether the pathologist rated the specimen as 2) cancer, 3) high-grade dysplasia (hgd), 4) hyperplastic polyps, 5) low-grade dysplasia (lgd), 6) non-informative (ni), or 7) serrated polyps. Each of these seven properties is binary, and multiple can be present per block. Provide your prediction as a list of confidence scores between 0 and 1, where:

1. 0 corresponds to False (polypectomy) and 1 corresponds to True (biopsy).
2. 0 corresponds to False (no cancer) and 1 corresponds to True (cancer).
3. 0 corresponds to False (no hgd) and 1 corresponds to True (hgd).
4. 0 corresponds to False (no hyperplastic polyps) and 1 corresponds to True (hyperplastic polyps).
5. 0 corresponds to False (no lgd) and 1 corresponds to True (lgd).
6. 0 corresponds to False (informative) and 1 corresponds to True (non-informative).
7. 0 corresponds to False (no serrated polyps) and 1 corresponds to True (serrated polyps).

Dataset: 4073 consecutive patients who received a histopathology diagnosis of colon biopsies between 1 January 2000 and 31 December 2009 at Radboudumc were included. For patients with multiple visits in this period, the first visit was selected. At the time of diagnosis, a set of 8832 tissue blocks was generated for these patients, each of which was individually diagnosed by gastrointestinal pathologists. In this work, we collected the conclusion of the pathology reports for each block in Dutch. We excluded 437 blocks due to missing reports (8), the report being English (4), or because the slide contained tissue from the ileum, duodenum, stomach, or small intestine (425). As a result, we included reports from 8395 blocks from 3925 patients. These blocks were described across 3925 reports. Each report conclusion was annotated by native Dutch data analysts under the supervision of two gastrointestinal pathologists (C.v.d.P. and I.N., 13-21 years of experience) into the classes: a) normal, b) hyperplastic polyps, c) low-grade dysplasia (lgd), d) high-grade dysplasia (hgd) e) cancer, f) non-informative and g) serrated polyps. Additionally, for each block, the procedure used to acquire the tissue, such as biopsy or polypectomy, was annotated. Previous studies[1,2,3] have utilized parts of this dataset, applying varying selection criteria, and have included additional details.

Dataset splits: 30% of patients were randomly selected for the test set (2519 blocks across 1177 reports) and the remaining 70% of patients (5876 blocks across 2748 reports) were used for model development with 5-fold cross-validation.

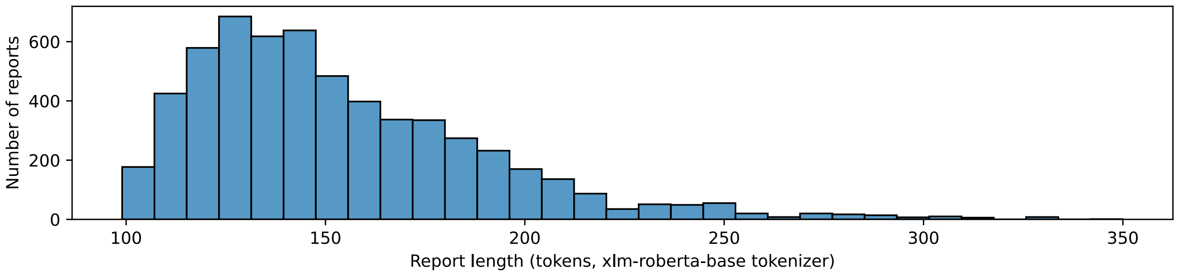

Supplementary Figure 16: Distribution of report lengths. The reports in the development dataset have a median length of 145 (95% CI: 106, 247) [min 99, max 350] tokens with the xlm-roberta-base tokenizer.

Metric: The labels for this task are binary for each of the eight properties. The frequency of the labels is highly unbalanced. Since the properties are not interchangeable, we calculate the Area Under the Receiver Operating Characteristic Curve (AUROC) for each property individually to evaluate model performance. The overall model performance is defined as the average of the individual AUROC values.

Supplementary References

1. Marini, N. *et al.* Unleashing the potential of digital pathology data by training computer-aided diagnosis models without human annotations. *npj Digit. Med.* **5**, 102 (2022).
2. Menotti, L. *et al.* Modelling digital health data: The ExaMode ontology for computational pathology. *Journal of Pathology Informatics* **14**, 100332 (2023).
3. Marchesin, S. *et al.* Empowering digital pathology applications through explainable knowledge extraction tools. *Journal of Pathology Informatics* **13**, 100139 (2022).

Example

Label: True, False, False, False, False, True, False

Notes: the report describes a biopsy (“Colonslijmvliesbiopten”) without specifying any of the other aspects, so it’s non-informative (ni).

Anonymous sample report:

[
"I",
"################################\nDISCLAIMER: \nTHIS REPORT HAS BEEN ANONYMIZED BY REPLACING PATIENT HEALTH INFORMATION WITH RANDOM SURROGATES.\nANY RESEMBLANCE TO REAL PERSONS, LIVING OR DEAD, IS PURELY COINCIDENTAL.\n################################\n\nColonslijmvliesbiopten: chronische ideopatische colitis, waarvan het histologische beeld beter past bij colitis ulcerosa dan bij morbus Crohn."
]

Task 16: RECIST lesion size presence

Background: The RECIST (Response Evaluation Criteria In Solid Tumors)[1,2] guidelines form the basis of standardized cancer response assessment by identifying and measuring up to five target lesions at baseline and comparing their dimensions in follow-up scans. Although these measurements are critical for determining response to treatment, the unstructured nature of many radiology reports complicates automated extraction. Before attempting precise extraction and standardization of measurements (e.g., identifying long-axis vs. short-axis diameters), it is useful to first establish whether a size measurement is present in the report at all. This initial classification step simplifies downstream processing and ensures more reliable evaluation: models first confirm that size information exists, and only then proceed to more demanding tasks such as extracting exact values. This division reduces confusion in performance scoring, clarifies how different models perform on distinct subtasks, and supports more robust and interpretable benchmarking.

Task: predict whether the size is mentioned for up to five of the target lesions. Provide your prediction as a list of confidence scores between 0 and 1, where:

8. 0 corresponds to False (no size for the first lesion) and 1 corresponds to True (size described for the first lesion).
9. 0 corresponds to False (no size for the second lesion) and 1 corresponds to True (size described for the second lesion).
10. 0 corresponds to False (no size for the third lesion) and 1 corresponds to True (size described for the third lesion).
11. 0 corresponds to False (no size for the fourth lesion) and 1 corresponds to True (size described for the fourth lesion).
12. 0 corresponds to False (no size for the fifth lesion) and 1 corresponds to True (size described for the fifth lesion).

Dataset: 253 radiology reports of the Radboudumc and 144 reports of the JBZ hospitals in the Netherlands were selected from archives containing thorax-abdomen CT scans conducted between 2000-2020. Reports were selected by filtering using regular expressions, searching for mention of 'RECIST', 'target-' or 'indicator-lesions'. Patients with more than one such scan in the archive were included. Reports were manually annotated by a trained investigator (M.J.J.d.G.) under the supervision of a radiologist (E.Th.S.) to determine whether the lesion sizes were mentioned in the report.

Dataset splits: 30% of patients were randomly selected for the test set (119 cases) and the remaining 70% of patients (278 cases) were used for model development with 5-fold cross-validation.

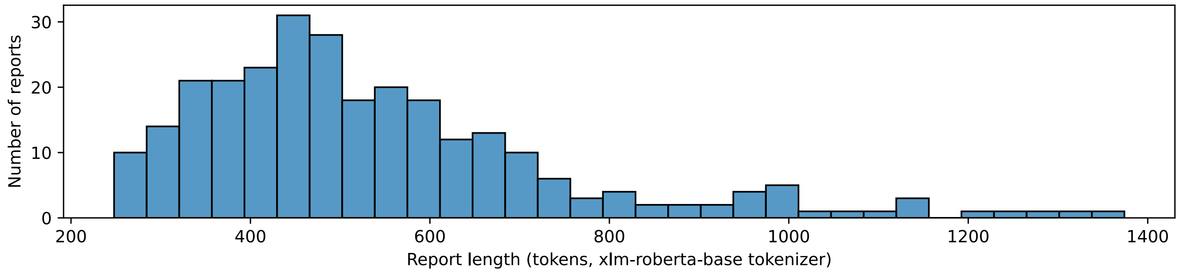

Supplementary Figure 17: Distribution of report lengths. The reports in the development dataset have a median length of 485 (95% CI: 268, 1127) [min 248, max 1374] tokens with the xlm-roberta-base tokenizer.

Metric: The labels for this task are binary for each of the five lesions. The frequency of the labels is moderately unbalanced. Since the task is the same for each lesion, we pool all predictions and labels. The Area Under the Receiver Operating Characteristic Curve (AUROC) is used to evaluate model performance.

Supplementary References

1. Eisenhauer, E. A. *et al.* New response evaluation criteria in solid tumours: Revised RECIST guideline (version 1.1). *European Journal of Cancer* **45**, 228–247 (2009).
2. Schwartz, L. H. *et al.* RECIST 1.1—Update and clarification: From the RECIST committee. *European Journal of Cancer* **62**, 132–137 (2016).

Example

Label: True, True, True, True, True

Notes: the report describes six target lesions, meaning all first five are present.

Anonymous sample report:
###############################
DISCLAIMER:
THIS REPORT HAS BEEN ANONYMIZED BY REPLACING PATIENT HEALTH INFORMATION WITH RANDOM SURROGATES.
ANY RESEMBLANCE TO REAL PERSONS, LIVING OR DEAD, IS PURELY COINCIDENTAL.
###############################

---Addendum start---
Target lesies: 12-06 Axillair links 68 x 40 mm (IMA 8-34) Axillair rechts 58 x 32 mm (IMA 3-38) Mediastinaal station 4R 27 x 14 mm (IMA 3-28) Precavaal 29 x 18 mm (IMA 8-104) Inguinaal links 36 x
22 mm (IMA 4-168) Inguinaal rechts 42 x 24 mm (IMA 4-172) SPD 5810 Non-target lesies: prominente
ring van Waldeyer, ongewijzigd craniocaudale afmeting van de milt bedraagt 11 cm Extranodaal: geen
Overige bevindingen: zie oorspronkelijk verslag
---Addendum einde---

Vraagstelling: Evaluatie tov CT 2 januari Evaluatie na 2 kuren chemotherapie

CT thorax abdomen, gescand na toediening van jodiumhoudend contrast intraveneus. Er is vergeleken met onderzoek van 5 maart en eerder.

Verslag:
CT-thorax en abdomen met intraveneus contrast. Ter correlatie de PET/CT
van dezelfde datum. Ter vergelijking de CT van 5 maart 2020.

Thorax.
Beiderzijds axillair conglomeraat van pathologisch vergrote lymfklieren,

met korte as diameter stand 35 mm rechts en 37 mm links. Tevens vergrote lymfklieren mediastinaal, twee van 14 mm op level 4 rechts. Tevens beiderzijds perihilair vergrote klieren en laag cervicaal enkele opvallende lymfklieren.
Gedilateerde atria, verwijde truncus pulmonalis en prominente aorta ascendens.
Coronaire sclerose. Focale gecalcificeerde atherosclerose in de aortaboog.
Scan in expiratie fase, waarbij matig beoordeelbaarheid van het longparenchym.
Bronchuswandverdikkingen en bronchiectasieen bij bronchopathie met versterkte interstitiele tekening en matglas.
Pulmonale laesies, met name rechts; subpleuraal 9 mm (9-101) en perifissuraal 6 mm (7-123) en 11 mm (11-159), meest waarschijnlijk lymfeklieren. Tevens ongewijzigde nodi van 5 mm (8-173), 7 mm (11-86) en 9 mm (11-189).
Inhomogene schildklier bij bekende calcificatie rechts, niet vergroot.

Abdomen.
Multipele opvallende lymfklieren abdominaal, waaronder in de leverhilus van 11 mm (3-86) en precavaal van 16 mm (8-104).
Multipele vergrote lymfklieren inguinaal en in de obturatorloge, met korte as diameter tot 19 mm links en de 24 mm rechts (4-168).
Bekende hyperperfusie in segment 6 van de lever. Slanke intrahepatische galwegen. Indruk van een heterogene lever, echter suboptimale scan fase ter beoordeling van de lever. Een en ander berust mogelijk nog op wisselende perfusie.
Bekend verwijde ductus choledochus, bij status na cholecystectomie?
Symmetrische aankleuring van de nieren. Bekende corticale intrekking rechts (9-193). Slank verzamelsysteem. Slanke bijnieren.
Normaal aspect van de milt. Geen splenomegalie.
Induratie van het mesenteriale vetweefsel.
Voorts normaal aspect van de tractus digestivus.
Atherosclerotische afwijkingen van de abdominale aorta en iliacaalvaten.
Normaal aspect van de blaas. Geen vrij vocht.

Skelet en weke delen.
Degeneratief aspect van het skelet. Geen ossale haarden.
Enkele intracutane noduli in de bekkenregio.

Impressie:

Multipele (sterk) vergrote lymfklieren verspreid over thorax en abdomen, met name beiderzijds axillair en inguinaal. Mogelijk in kader van progressie van ziekte bij chronische lymfatische leukemie.

Autorisatiedatum:
9 juni Contrastmiddel: Optiray 300: 100 cc

Task 17: Pancreatic ductal adenocarcinoma attributes

Background: Pancreatic ductal adenocarcinoma (PDAC) is estimated to become the second leading cause of cancer-related deaths in Western countries by 2030[1]. Due to the lack of early, disease-specific symptoms, 80–85% of patients are diagnosed in advanced disease stages (stages III-IV). However, patients diagnosed at an early stage (stage I) present a significantly more favorable prognosis than stage IV patients (median survival: 26 vs. 4.8 months), making early detection the current most effective approach to improving outcomes[2]. Abdominal contrast-enhanced computed tomography (CECT) scans are usually the first line of diagnosis for PDAC, as there are no validated early diagnostic biomarkers. Automatic detection of PDAC on CECT scans is crucial to improving early detection on routine abdominal imaging. Artificial intelligence (AI) algorithms show great potential for this task but require large annotated data sets for model training. Since PDAC imaging can be done with multiple contrast CT protocols, the first step to curating large, representative data sets for model training is parsing radiology reports to extract the PDAC diagnosis, and information regarding the tumor (lesion attenuation and location). This is a straightforward task for trained investigators, but tedious to perform manually for thousands of reports.

Task: predict the attributes of the pancreatic ductal adenocarcinoma (PDAC): attenuation (iso/hyper/hypo/not mentioned) and location (head/body/tail/not mentioned). Provide your prediction as a list of strings, being:

13. "iso", "hyper", "hypo", or "not mentioned"
14. "head", "body", "tail", or "not mentioned"

Dataset: 2343 consecutive patients undergoing pancreatic and liver CECT scans between 1 January 2011 and 31 December 2022 at Radboud University Medical Center were included. Additionally, all CT scans for 1076 consecutive patients who underwent histopathology analysis of pancreas specimens or distant metastasis of pancreatic cancer between 1 January 2006 and 31 December 2022 at Radboud University Medical Center were retrospectively collected. This resulted in 3419 selected patients. For each patient, the baseline diagnostic scan was considered for positive cases and the most recent scan mentioning a normal pancreas was considered for negative cases. 121 patients were excluded for the absence of a baseline diagnostic report or no mention of the pancreas. This is the dataset used also in the PANORAMA study[3]. 2035 cases (2035 patients) were sampled and manually annotated by one of three trained investigators (N.A., M.Sch., I.M.E.S.). The 651 cases with a PDAC lesion were selected for this task. 54 cases were excluded for the absence of a radiology report. This included 597 cases (597 patients) for this task.

Dataset splits: 30% of patients were randomly selected for the test set (179 cases) and the remaining 70% of patients (418 cases) were used for model development with 5-fold cross-validation.

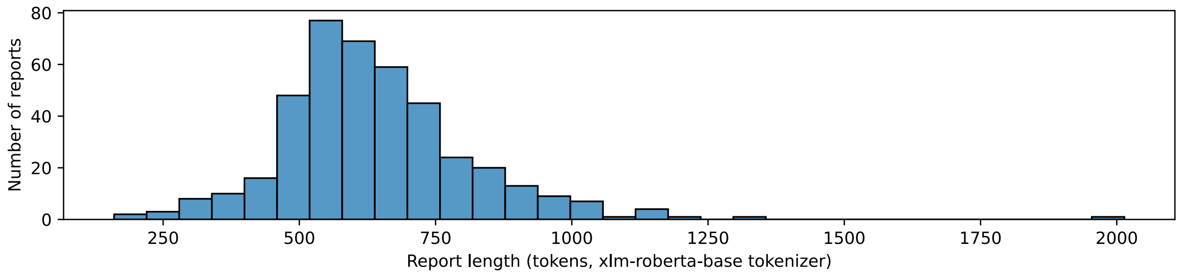

Supplementary Figure 18: Distribution of report lengths. The reports in the development dataset have a median length of 615 (95% CI: 324, 1021) [min 160, max 2014] tokens with the xlm-roberta-base tokenizer.

Metric: The labels are multi-class for each attribute and non-ordinal. The frequency of the labels is unbalanced. Therefore, the Unweighted Kappa is used to evaluate model performance.

Supplementary References

1. Ryan, D. P., Hong, T. S. & Bardeesy, N. Pancreatic Adenocarcinoma. *N Engl J Med* **371,** 1039–1049 (2014).
2. Schwartz, N. R. M. *et al.* Potential Cost-Effectiveness of Risk-Based Pancreatic Cancer Screening in Patients With New-Onset Diabetes. *Journal of the National Comprehensive Cancer Network* **20,** 451–459 (2022).
3. Alves, N. *et al. The PANORAMA Study Protocol: Pancreatic Cancer Diagnosis - Radiologists Meet AI*. https://zenodo.org/doi/10.5281/zenodo.10599559 (2024) doi:10.5281/ZENODO.10599559.

Example

Label: hypo, body

Notes: the report describes a pancreas lesion of 43 mm with hypo attenuation located in the pancreas body ("Hypovasculaire tumor in pancreascorpus (43 mm)").

Anonymous sample report:
################################
DISCLAIMER:
THIS REPORT HAS BEEN ANONYMIZED BY REPLACING PATIENT HEALTH INFORMATION WITH RANDOM SURROGATES.
ANY RESEMBLANCE TO REAL PERSONS, LIVING OR DEAD, IS PURELY COINCIDENTAL.
################################
Gerichte vraagstelling: metastase?
Verslag:
Herbeoordeling CT d.d. 20 maart 2006. Hypovasculaire tumor in pancreascorpus en staart (43 mm) met occlusie vena lienalis; mesenteriale en perigastrische collateralen; vervorming portomesenterisch confluens met doorgroei langs VMS; volledige encasement truncus coeliacus en a. lienalis met irregulaire contour; partiele encasement art. Hepatica communis; doorgroei richting aorticorenale plexus rechts. Enkele kleine lymfklieren craniaal van de truncus coeliacus (6 mm) Normale grootte van de lever, met meerdere cysten. Hypodense lesie in leversegment 5 (32 mm, 204-236). Degeneratief skelet. Dikke galwegen. Normaal aspect galblaas. Normaal aspect bijnieren. Normale aankleuring en grootte van de nieren met meerdere corticale cysten. Arteriosclerose grote retroperitoneale vaten. Normaal aspect urineblaas. Normale grootte prostaat en zaadblazen. Verkalkingen in vas deferens rechts. Diverticulosis colon sigmoideum. Volumeverlies linker onderkwab. Geen pleuravocht en/of pericardvocht.
Conclusie.
Hypovasculaire tumor in pancreascorpus (43 mm) met doorgroei in de mesenteriale wortel en encasement truncus coeliacus, a. hepatica communis en art. Lienalis; doorgroei richting aorticorenale plexus rechts. Waarschijnlijk metastase in leversegment 7 (32 mm).

Task 18: Hip Kellgren-Lawrence scoring

Background: Radiology reports for hip osteoarthritis describe the disease's features (e.g., there are some osteophytes) and/or the presence or absence of the disease (e.g., moderate hip osteoarthritis). From these descriptions, a Kellgren-Lawrence score can be derived per hip joint: 0 (no osteoarthritis), 1 (doubtful osteoarthritis), 2 (minimal osteoarthritis), 3 (moderate osteoarthritis), and 4 (severe osteoarthritis). Such a score is clinically relevant to both diagnose the disease and decide if surgery is needed. A Kellgren-Lawrence grade from 2 to 4 indicates presence of hip osteoarthritis, while only grades 3 and 4 lead to surgery (only if the patient has pain). The classification is important for developing deep learning classifiers, given that we can get the labels of hip X-ray images through obtaining the Kellgren-Lawrence scores from the reports.

Task: Predict the Kellgren-Lawrence score based on the radiology report. Provide your prediction as a list of strings, being:

15. "0", "1", "2", "3", "4", "p", or "n"
16. "0", "1", "2", "3", "4", "p", or "n"

Dataset: 34,601 consecutive studies (19,964 consecutive patients) were selected from patients who underwent hip X-ray at Radboudumc between 1st January 2002 and 31st December 2022. Studies with up to three X-ray images, without bilateral hip replacement and with more than one keyword of 'joint gap', 'degeneration', and 'coxartr' in the report were included. Cases from patients younger than 30 at the time of the X-ray were excluded, given that hip osteoarthritis is not common at this age and thus the reports refer mostly to other aspects, such as hip dysplasia. Then, from the selection, we randomly sampled 172 studies (159 patients) for testing. From the same selection, 4806 studies (4129 patients) were selected for model development (without patient overlap with the test set). All reports for the test set were manually annotated by a trained investigator (M.N.P.). The reports that were difficult to annotate were revised by a radiologist (M.J.C.M.R.). The reports for model development were annotated with ChatGPT (with GPT-4 on 13 June 2023) inputting the prompt below*, which was revised by the radiologist too. From the development data, 3 cases were excluded, due to missing annotations (1), or having a duplicate report (2). This included 4803 cases (4127 patients) for development.

Dataset splits: 172 cases were selected for the test set (159 patients) and 4803 cases were selected (4127 patients) for model development with 5-fold cross-validation.

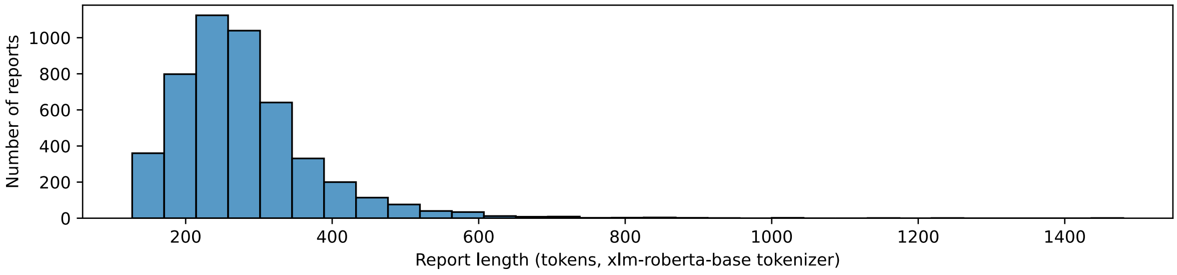

Supplementary Figure 19: Distribution of report lengths. The reports in the development dataset have a median length of 261 (95% CI: 153, 519) [min 127, max 1480] tokens with the xlm-roberta-base tokenizer.

Metric: The labels for this dataset range from 0 up to and including 4 for the Kellgren-Lawrence score, and additionally include "hip prosthesis" and "not determinable". The frequency of the labels is imbalanced. The Kellgren-Lawrence score is ordinal, but "hip prosthesis" and "not determinable" are not. Therefore, we use the Unweighted Kappa to evaluate model performance.

*Prompt inputted to GPT-4 to peform the annotations:
You are a helpful Dutch medical assistant. You will help the user assess hip osteoarthritis in radiology reports using the Kellgren Lawrence scale. Below is a brief description of the Kellgren Lawrence scale:

- 0: no radiographic core features of osteoarthritis, no joint gap narrowing, no bone abnormalities. Keywords: no coxarthrosis
- 1: possible joint gap narrowing, possible osteophyte formation. Keywords: no obvious coxarthrosis
- 2: obvious osteophyte formation, possible joint gap narrowing. Keywords: minimal coxarthrosis, incipient coxarthrosis, mild coxarthrosis, minor coxarthrosis
- 3: moderate osteophyte formation, marked joint gap narrowing and some sclerosis, possible degenerative bone defects. Keywords: moderate coxarthrosis
- 4: large definite osteophytes, definite joint gap narrowing and severe sclerosis, definite degenerative bone defects. Keywords: end-stage coxarthrosis, severe coxarthrosis, substantial coxarthrosis, strong coxarthrosis, obvious degeneration, obvious osteophyte formation
- not applicable: there is not enough information in the report to give an assessment
- prosthesis: the patient has a hip prosthesis. Keywords: THP, THR, total hip replacement, total hip prosthesis

Always answer in the following form:
left:
right:

Always choose from one of the following options for the left and right grade: [0, 1, 2, 3, 4, not applicable, prosthesis].

Keep your motivation short but complete and clear. For example:
left: 3
right: 4

left: prosthesis
right: 4

Below are some more important things to watch out for:
'bdz' stands for bilateral.
Cox osteoarthritis and pelvic osteoarthritis are synonyms.
If a single condition is listed for a grade, that is enough to get that grade. 'Severe sclerosis' is therefore grade 4, even though joint gap narrowing is not mentioned.
Joint gap narrowing always results in grade 3 or above, 2 or below is not possible.
Cox osteoarthritis always results in grade 2 or higher, 1 or lower is not possible.
Sclerosis always results in grade 3 or higher, 2 or lower is not possible.
Always choose the highest possible grade.
'Moderate to severe coxarthrosis' is therefore grade 4.

This is the report to review:
report

Example

Label left: 4

Label right: p

Notes: the “p” for the right label stands for “prosthesis”.

Anonymous sample report:
##############################
DISCLAIMER:
THIS REPORT HAS BEEN ANONYMIZED BY REPLACING PATIENT HEALTH INFORMATION WITH RANDOM SURROGATES.
ANY RESEMBLANCE TO REAL PERSONS, LIVING OR DEAD, IS PURELY COINCIDENTAL.
##############################
Medische gegevens: Krijgt steeds meer pijnklachten in
de li heup, bij lopen meer pijn, uitstralend naar de lies; zeurende
continue pijn li zijkant; kan er wel op ligggen. Bekend met poly-artrose.
X-bekken, en axiale opnamen beiderzijds.
Vergeleken wordt met foto van 9.11.2021.
Er zijn sclerotische veranderingen zichtbaar, samen met vernauwing van de gewrichtsspleet en de vorming van osteofyten in het linker acetabulum. Daarnaast is er cystevorming in de kop van het linker femur. De rechterheup heeft een eerder geplaatste gecementeerde totale heupprothese, waarvan de positie ongewijzigd is en er geen tekenen van loslating zijn. De overige botstructuren vertonen een normale contour en botdichtheid. In het rechter bovenbeen zijn twee verkalkingen aanwezig. In het kleine bekken bevinden zich flebolieten.
Conclusie: Uitgesproken coxartrose linker heup. Status quo ante heupprothese rechts.

Task 19: Prostate volume extraction

Background: Prostate cancer has 1.4 million new cases each year[1], a high incidence-to-mortality ratio and risks associated with treatment and biopsy; making non-invasive diagnosis of clinically significant prostate cancer (csPCa) crucial to reduce both unnecessary (confirmatory) biopsies and overtreatment[2]. Multiparametric MRI (mpMRI) scans interpreted by expert prostate radiologists provide the best non-invasive diagnosis[3]. The diagnostic value of MRI is increased when used in combination with the prostate-specific antigen (PSA) density[4]. When the PSA density is not explicitly described in the report, it can be calculated based on the PSA level and prostate volume. Extraction of the prostate volume from radiology reports is straightforward for investigators with the relevant clinical knowledge, but tedious to perform for thousands of reports.

Task: predict the prostate volume (in cm^3) based on the radiology report. When dimensions are given (e.g., "3 x 4 x 5 mm"), we used the ellipsoid formula to calculate the volume ($\pi \cdot l \cdot w \cdot h/6$), with l, w, and h the spatial dimensions in mm. Provide your prediction as a float.

Dataset: This dataset is constructed from three sources:

7. Radboudumc. The PI-CAI Challenge[5] was hosted in 2022. The PI-CAI Public and Private Training dataset contains 9107 cases for which lesion-level scores and clinical parameters were manually annotated by trained investigators (I.S., A.S., J.J.T., J.S.B., G.S., and E.M.G.T.) under supervision of a radiologist with 7 years of experience with prostate MRI (M.d.R.). For this task, we selected all cases with a radiology report from Radboudumc, selecting 6578 cases (5679 patients). Cases from Ziekenhuisgroep Twente were excluded to prevent overlap between the pretraining dataset and benchmark. Cases from University Medical Center Groningen were excluded because radiology reports were not available for the PI-CAI Public and Private Training dataset. 68 cases were excluded due to the report being English (4) or the prostate volume not being mentioned in the report (64). This included 6510 cases (5636 patients).
8. University Medical Center Groningen. 416 consecutive cases (392 patients) were selected from patients who had a suspicion for prostate cancer (elevated PSA levels, and/or abnormal digital rectal examination findings, and/or lower urinary tract symptoms) between 1 January 2021 and 31 December 2022 at University Medical Center Groningen. 53 cases were excluded because of previous prostate cancer treatment (24), missing PI-RADS values in the report (14), missing prostate volume values in the report (14) or not being a detection study (1). This selected 363 cases (354 patients). Findings were manually annotated by trained investigators (C.R., S.J.F., Q.Y.v.L., J.S.B.) , under supervision of a radiologist with 9 years of experience with prostate MRI (D.Y.).
9. Antoni van Leeuwenhoek Hospital. 9766 consecutive cases (7841 patients) were selected from patients who had a suspicion of prostate cancer (elevated PSA levels, and/or abnormal digital rectal examination findings, and/or lower urinary tract symptoms) between 1 January 2012 and 17 February 2023 at Antoni van Leeuwenhoek Ziekenhuis. 273 cases (269 patients) were randomly sampled. Additionally, 250 consecutive cases (250 patients) between 14 October 2015 and 3 March 2016 were selected. 9 cases were selected in both sets and only included once. This selected 514 cases (498 patients), which were manually annotated by a trained investigator (C.R.) , under supervision of a radiologist with 9 years of experience with prostate MRI (D.Y.). 79 cases were excluded, due to not being a prostate MRI detection study (25), having no prostate volume reported (53), or having an ambiguous prostate volume (1). This included 435 cases (426 patients) for this task.

Combining these data sources included 7308 cases (6416 patients).

Dataset splits: 30% of patients were randomly selected for the test set (2170 cases) and the remaining 70% of patients (5138 cases) were used for model development with 5-fold cross-validation.

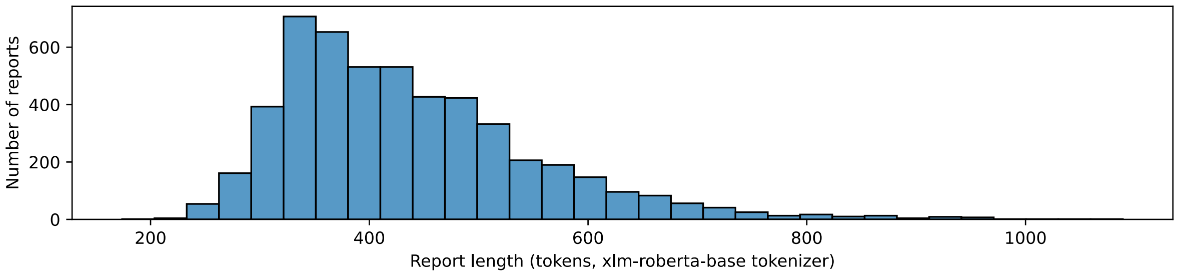

Supplementary Figure 20: Distribution of report lengths. The reports in the development dataset have a median length of 414 (95% CI: 278, 717) [min 174, max 1089] tokens with the xlm-roberta-base tokenizer.

Metric: The labels for this task are floating point values ranging from 4.0 to 470 cm^3, with 95% of values between 24 and 181 cm^3 (ranges based on the development data). The distribution of labels is skewed. The Robust Symmetric Mean Absolute Percentage Error Score (RSMAPES) with an epsilon of 4 cm^3 is used to evaluate model performance. This evaluates the prostate volume prediction in a diagnostically relevant manner.

Supplementary References

1. Sung, H. *et al.* Global Cancer Statistics 2020: GLOBOCAN Estimates of Incidence and Mortality Worldwide for 36 Cancers in 185 Countries. *CA A Cancer J Clinicians* **71**, 209–249 (2021).
2. Stavrinides, V., Giganti, F., Emberton, M. & Moore, C. M. MRI in active surveillance: a critical review. *Prostate Cancer Prostatic Dis* **22**, 5–15 (2019).
3. Eldred-Evans, D. *et al.* Population-Based Prostate Cancer Screening With Magnetic Resonance Imaging or Ultrasonography: The IP1-PROSTAGRAM Study. *JAMA Oncol* **7**, 395 (2021).
4. Distler, F. A. *et al.* The Value of PSA Density in Combination with PI-RADS^{TM} for the Accuracy of Prostate Cancer Prediction. *Journal of Urology* **198**, 575–582 (2017).
5. Saha, A. *et al.* Artificial intelligence and radiologists in prostate cancer detection on MRI (PI-CAI): an international, paired, non-inferiority, confirmatory study. *The Lancet Oncology* **25**, 879–887 (2024).

Example

Label: 68

Notes: The report describes a prostate volume of 68 cm^3 ("De prostaat heeft een volume van 68 cc").

Anonymous sample report:

##############################
DISCLAIMER:
THIS REPORT HAS BEEN ANONYMIZED BY REPLACING PATIENT HEALTH INFORMATION WITH RANDOM SURROGATES.
ANY RESEMBLANCE TO REAL PERSONS, LIVING OR DEAD, IS PURELY COINCIDENTAL.
##############################
Klinische gegevens:
PSA: 9,7; geen eerdere TRUS biopt(en); klinisch stadium: T 0 .
Verslag:
Er werd gescand met het detectie-protocol: locale prostaat T2WI (drie richtingen), DWI en DCE (Gadovist i.v.).
Bevindingen:
Index laesie mark1:
Plaats: rechts apicaal ventraal
Laesie grootte: 13 x 9 x 7mm.
T2W/DWI/DCE score: 4/4/-.
Minimale ADC waarde: 634 (normaal groter dan 950).
Risico categorie: Intermediate/high-grade Cancer (PIRADS v2 categorie: 4).
Onzekere extra-prostatische uitbreiding.
Impressie:
De prostaat heeft een volume van 68 cc. PSA densiteit: 0,14.
Afwijking passend bij significant carcinoom rechts ventraal apicaal (PIRADS 4). Onzekere extra-prostatische uitbreiding.

Task 20: Prostate specific antigen extraction

Background: Prostate cancer has 1.4 million new cases each year[1], a high incidence-to-mortality ratio and risks associated with treatment and biopsy; making non-invasive diagnosis of clinically significant prostate cancer (csPCa) crucial to reduce both unnecessary (confirmatory) biopsies and overtreatment[2]. Multiparametric MRI (mpMRI) scans interpreted by expert prostate radiologists provide the best non-invasive diagnosis[3]. The diagnostic value of MRI is increased when used in combination with the prostate-specific antigen (PSA) density[4]. When the PSA level is not explicitly described in the report, it can be calculated based on the PSA level and prostate volume. Extraction of the PSA level from radiology reports is straightforward for investigators with the relevant clinical knowledge, but tedious to perform for thousands of reports.

Task: predict the PSA level based on the radiology report. When a range is given (e.g., "PSA: 4-5"), provide the average (i.e., 4.5). Provide your prediction as a float.

Dataset: This dataset is constructed from three sources:

10. Radboudumc. The PI-CAI Challenge[5] was hosted in 2022. The PI-CAI Public and Private Training dataset contains 9107 cases for which lesion-level scores and clinical parameters were manually annotated by trained investigators (I.S., A.S., J.J.T., J.S.B., G.S., and E.M.G.T.) under supervision of a radiologist with 7 years of experience with prostate MRI (M.d.R.). For this task, we selected all cases with a radiology report from Radboudumc, selecting 6578 cases (5679 patients). Cases from Ziekenhuisgroep Twente were excluded to prevent overlap between the pretraining dataset and benchmark. Cases from University Medical Center Groningen were excluded because radiology reports were not available for the PI-CAI Public and Private Training dataset. 247 cases were excluded due to the report being English (4) or the PSA level not being mentioned in the report (243). This included 6331 cases (5529 patients).
11. University Medical Center Groningen. 416 consecutive cases (392 patients) were selected from patients who had a suspicion for prostate cancer (elevated PSA levels, and/or abnormal digital rectal examination findings, and/or lower urinary tract symptoms) between 1 January 2021 and 31 December 2022 at University Medical Center Groningen. 178 cases were excluded because of previous prostate cancer treatment (24), missing PI-RADS values in the report (14), missing PSA values in the report (139), or not being a detection study (1). This selected 238 cases (235 patients). Findings were manually annotated by trained investigators (C.R., S.J.F., Q.Y.v.L., J.S.B.) , under supervision of a radiologist with 9 years of experience with prostate MRI (D.Y.).
12. Antoni van Leeuwenhoek Hospital. 9766 consecutive cases (7841 patients) were selected from patients who had a suspicion of prostate cancer (elevated PSA levels, and/or abnormal digital rectal examination findings, and/or lower urinary tract symptoms) between 1 January 2012 and 17 February 2023 at Antoni van Leeuwenhoek Ziekenhuis. 273 cases (269 patients) were randomly sampled. Additionally, 250 consecutive cases (250 patients) between 14 October 2015 and 3 March 2016 were selected. 9 cases were selected in both sets and only included once. This selected 514 cases (498 patients), which were manually annotated by a trained investigator (C.R.) , under supervision of a radiologist with 9 years of experience with prostate MRI (D.Y.). 278 cases were excluded, due to not being a prostate MRI detection study (25), having no PSA level reported (251), or having an ambiguous PSA level (2). This included 238 cases (231 patients) for this task.

Combining these data sources included 6805 cases (5993 patients).

Dataset splits: 30% of patients were randomly selected for the test set (2046 cases) and the remaining 70% of patients (4759 cases) were used for model development with 5-fold cross-validation.

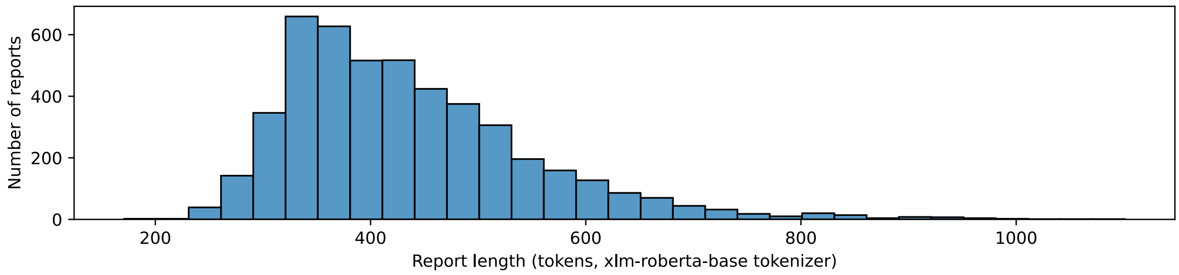

Supplementary Figure 21: Distribution of report lengths. The reports in the development dataset have a median length of 413 (95% CI: 277, 711) [min 171, max 1101] tokens with the xlm-roberta-base tokenizer.

Metric: The labels for this task are floating point values ranging from 0 to 870 ng/mL, with 95% of values between 2 and 35 ng/mL (ranges based on the development data). The distribution of labels is highly skewed. The Robust Symmetric Mean Absolute Percentage Error Score (RSMAPES) with an epsilon of 0.4 ng/ML is used to evaluate model performance. This evaluates the prostate-specific antigen prediction in a diagnostically relevant manner.

Supplementary References

1. Sung, H. *et al.* Global Cancer Statistics 2020: GLOBOCAN Estimates of Incidence and Mortality Worldwide for 36 Cancers in 185 Countries. *CA A Cancer J Clinicians* **71**, 209–249 (2021).
2. Stavrinides, V., Giganti, F., Emberton, M. & Moore, C. M. MRI in active surveillance: a critical review. *Prostate Cancer Prostatic Dis* **22**, 5–15 (2019).
3. Eldred-Evans, D. *et al.* Population-Based Prostate Cancer Screening With Magnetic Resonance Imaging or Ultrasonography: The IP1-PROSTAGRAM Study. *JAMA Oncol* **7**, 395 (2021).
4. Distler, F. A. *et al.* The Value of PSA Density in Combination with PI-RADS™ for the Accuracy of Prostate Cancer Prediction. *Journal of Urology* **198**, 575–582 (2017).
5. Saha, A. *et al.* Artificial intelligence and radiologists in prostate cancer detection on MRI (PI-CAI): an international, paired, non-inferiority, confirmatory study. *The Lancet Oncology* **25**, 879–887 (2024).

Example

Label: 9.7

Notes: The report describes a PSA level of 9.7 ng/ml ("PSA: 9,7").

Anonymous sample report:

##############################
DISCLAIMER:
THIS REPORT HAS BEEN ANONYMIZED BY REPLACING PATIENT HEALTH INFORMATION WITH RANDOM SURROGATES.
ANY RESEMBLANCE TO REAL PERSONS, LIVING OR DEAD, IS PURELY COINCIDENTAL.
##############################
Klinische gegevens:
PSA: 9,7; geen eerdere TRUS biopt(en); klinisch stadium: T 0 .
Verslag:
Er werd gescand met het detectie-protocol: locale prostaat T2WI (drie richtingen), DWI en DCE (Gadovist i.v.).
Bevindingen:
Index laesie mark1:
Plaats: rechts apicaal ventraal
Laesie grootte: 13 x 9 x 7mm.
T2W/DWI/DCE score: 4/4/-.
Minimale ADC waarde: 634 (normaal groter dan 950).
Risico categorie: Intermediate/high-grade Cancer (PIRADS v2 categorie: 4).
Onzekere extra-prostatische uitbreiding.
Impressie:
De prostaat heeft een volume van 68 cc. PSA densiteit: 0,14.
Afwijking passend bij significant carcinoom rechts ventraal apicaal (PIRADS 4). Onzekere extra-prostatische uitbreiding.

Task 21: Prostate specific antigen density extraction

Background: Prostate cancer has 1.4 million new cases each year[1], a high incidence-to-mortality ratio and risks associated with treatment and biopsy; making non-invasive diagnosis of clinically significant prostate cancer (csPCa) crucial to reduce both unnecessary (confirmatory) biopsies and overtreatment[2]. Multiparametric MRI (mpMRI) scans interpreted by expert prostate radiologists provide the best non-invasive diagnosis[3]. The diagnostic value of MRI is increased when used in combination with the prostate-specific antigen (PSA) density[4]. Extraction of the PSA density from radiology reports is straightforward for investigators with the relevant clinical knowledge, but tedious to perform for thousands of reports.

Task: predict the PSA density, which is either directly described in the radiology report, or needs to be calculated based on the PSA level and prostate volume. All required information is provided in the report, and the PSA density is related to the PSA and prostate volume as: PSA density = PSA / prostate volume. Provide your prediction as a float.

Dataset: This dataset is constructed from three sources:

13. Radboudumc. The PI-CAI Challenge[5] was hosted in 2022. The PI-CAI Public and Private Training dataset contains 9107 cases for which lesion-level scores and clinical parameters were manually annotated by trained investigators (I.S., A.S., J.J.T., J.S.B., G.S., and E.M.G.T.) under supervision of a radiologist with 7 years of experience with prostate MRI (M.d.R.). For this task, we selected all cases with a radiology report from Radboudumc, selecting 6578 cases (5679 patients). Cases from Ziekenhuisgroep Twente were excluded to prevent overlap between the pretraining dataset and benchmark. Cases from University Medical Center Groningen were excluded because radiology reports were not available for the PI-CAI Public and Private Training dataset. 374 cases were excluded due to the report being English (4) or the PSA density not being mentioned in the report (370). This included 6204 cases (5439 patients).
14. University Medical Center Groningen. 416 consecutive cases (392 patients) were selected from patients who had a suspicion for prostate cancer (elevated PSA levels, and/or abnormal digital rectal examination findings, and/or lower urinary tract symptoms) between 1 January 2021 and 31 December 2022 at University Medical Center Groningen. 88 cases were excluded because of previous prostate cancer treatment (24), missing PI-RADS values in the report (14), missing PSA density in the report (49), or not being a detection study (1). This selected 328 cases (321 patients). Findings were manually annotated by trained investigators (C.R., S.J.F., Q.Y.v.L., J.S.B.) , under supervision of a radiologist with 9 years of experience with prostate MRI (D.Y.).
15. Antoni van Leeuwenhoek Hospital. 9766 consecutive cases (7841 patients) were selected from patients who had a suspicion of prostate cancer (elevated PSA levels, and/or abnormal digital rectal examination findings, and/or lower urinary tract symptoms) between 1 January 2012 and 17 February 2023 at Antoni van Leeuwenhoek Ziekenhuis. 273 cases (269 patients) were randomly sampled. Additionally, 250 consecutive cases (250 patients) between 14 October 2015 and 3 March 2016 were selected. 9 cases were selected in both sets and only included once. This selected 514 cases (498 patients), which were manually annotated by a trained investigator (C.R.), under supervision of a radiologist with 9 years of experience with prostate MRI (D.Y.). 326 cases were excluded, due to not being a prostate MRI detection study (25) or having no PSA density reported (301). This included 188 cases (187 patients) for this task.

Combining these data sources included 6720 cases (5947 patients).

Dataset splits: 30% of patients were randomly selected for the test set (2020 cases) and the remaining 70% of patients (4700 cases) were used for model development with 5-fold cross-validation.

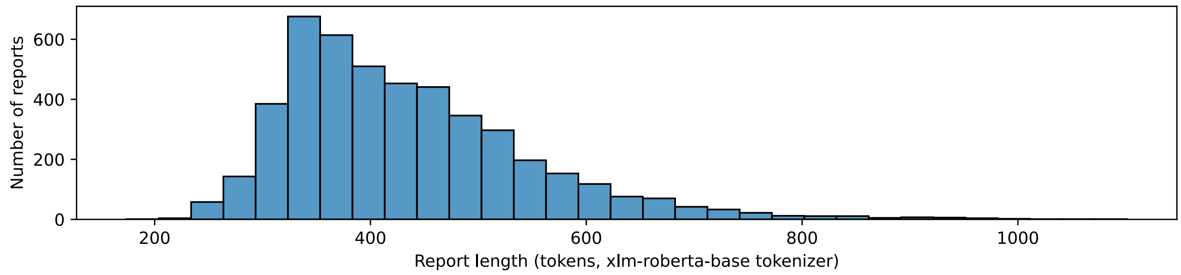

Supplementary Figure 22: Distribution of report lengths. The reports in the development dataset have a median length of 411 (95% CI: 276, 711) [min 174, max 1101] tokens with the xlm-roberta-base tokenizer.

Metric: The labels for this task are floating point values ranging from 0 to 171 ng/mL^2, with 95% of values between 0.03 and 0.6 ng/mL^2 (ranges are based on the development data). The distribution of labels is highly skewed. The Robust Symmetric Mean Absolute Percentage Error Score (RSMAPES) with an epsilon of 0.04 ng/mL^2 is used to evaluate model performance. This evaluates the prostate-specific antigen density prediction in a diagnostically relevant manner.

Supplementary References

1. Sung, H. *et al.* Global Cancer Statistics 2020: GLOBOCAN Estimates of Incidence and Mortality Worldwide for 36 Cancers in 185 Countries. *CA A Cancer J Clinicians* **71**, 209–249 (2021).
2. Stavrinides, V., Giganti, F., Emberton, M. & Moore, C. M. MRI in active surveillance: a critical review. *Prostate Cancer Prostatic Dis* **22**, 5–15 (2019).
3. Eldred-Evans, D. *et al.* Population-Based Prostate Cancer Screening With Magnetic Resonance Imaging or Ultrasonography: The IP1-PROSTAGRAM Study. *JAMA Oncol* **7**, 395 (2021).
4. Distler, F. A. *et al.* The Value of PSA Density in Combination with PI-RADS™ for the Accuracy of Prostate Cancer Prediction. *Journal of Urology* **198**, 575–582 (2017).
5. Saha, A. *et al.* Artificial intelligence and radiologists in prostate cancer detection on MRI (PI-CAI): an international, paired, non-inferiority, confirmatory study. *The Lancet Oncology* **25**, 879–887 (2024).

Example

Label: 0.14

Notes: The report describes a PSA density of 0.14 ng/ml^2 ("PSA densiteit: 0,14").

Anonymous sample report:

##############################
DISCLAIMER:
THIS REPORT HAS BEEN ANONYMIZED BY REPLACING PATIENT HEALTH INFORMATION WITH RANDOM SURROGATES.
ANY RESEMBLANCE TO REAL PERSONS, LIVING OR DEAD, IS PURELY COINCIDENTAL.
##############################
Klinische gegevens:
PSA: 9,7; geen eerdere TRUS biopt(en); klinisch stadium: T 0 .
Verslag:
Er werd gescand met het detectie-protocol: locale prostaat T2WI (drie richtingen), DWI en DCE (Gadovist i.v.).
Bevindingen:
Index laesie mark1:
Plaats: rechts apicaal ventraal
Laesie grootte: 13 x 9 x 7mm.
T2W/DWI/DCE score: 4/4/-.
Minimale ADC waarde: 634 (normaal groter dan 950).
Risico categorie: Intermediate/high-grade Cancer (PIRADS v2 categorie: 4).
Onzekere extra-prostatische uitbreiding.
Impressie:
De prostaat heeft een volume van 68 cc. PSA densiteit: 0,14.
Afwijking passend bij significant carcinoom rechts ventraal apicaal (PIRADS 4). Onzekere extra-prostatische uitbreiding.

Task 22: Pancreatic ductal adenocarcinoma size measurement

Background: Pancreatic ductal adenocarcinoma (PDAC) is estimated to become the second leading cause of cancer-related deaths in Western countries by 2030[1]. Due to the lack of early, disease-specific symptoms, 80–85% of patients are diagnosed in advanced disease stages (stages III-IV). However, patients diagnosed at an early stage (stage I) present a significantly more favorable prognosis than stage IV patients (median survival: 26 vs. 4.8 months), making early detection the current most effective approach to improving outcomes[2]. Abdominal contrast-enhanced computed tomography (CECT) scans are usually the first line of diagnosis for PDAC, as there are no validated early diagnostic biomarkers. The most used method for PDAC staging is the TNM classification by the American Joint Committee on Cancer (AJCC). The local tumor size (T stage), the dissemination to the regional lymph nodes (N stage), and the metastatic spread to distant sites (M stage) are used to stratify patients, determine their prognosis, and indicate treatment and monitoring strategy[3]. Since tumor size has been shown to correlate with survival, it is crucial to consider when developing artificial intelligence (AI) algorithms for pancreatic cancer management. However, manually extracting the size from radiology reports is time-consuming, limiting the size of data sets available for algorithm training.

Task: predict the reported diameter of the PDAC in mm, as described in the radiology report. When multiple axes are measured (e.g., “12 x 34 mm”), report the longest axis size (i.e., 34). When a range is given for one axis (e.g., “1 to 2 mm”), provide the average (i.e., 1.5). Provide your prediction as a float.

Dataset: 2343 consecutive patients undergoing pancreatic and liver CECT scans between 1 January 2011 and 31 December 2022 at Radboud University Medical Center were included. Additionally, all CT scans for 1076 consecutive patients who underwent histopathology analysis of pancreas specimens or distant metastasis of pancreatic cancer between 1 January 2006 and 31 December 2022 at Radboud University Medical Center were retrospectively collected. This resulted in 3419 selected patients. For each patient, the baseline diagnostic scan was considered for positive cases and the most recent scan mentioning a normal pancreas was considered for negative cases. 121 patients were excluded for the absence of a baseline diagnostic report or no mention of the pancreas. This is the dataset used also in the PANORAMA study[4]. 2035 cases (2035 patients) were sampled and manually annotated by one of three trained investigators (N.A., M.Sch., I.M.E.S.). The 651 cases with a PDAC lesion were selected for this task. 139 cases were excluded, for the absence of a radiology report (32) or the PDAC lesion size not mentioned in the report (107). This included 490 cases (490 patients) for this task.

Dataset splits: 30% of patients were randomly selected for the test set (147 cases) and the remaining 70% of patients (343 cases) were used for model development with 5-fold cross-validation.

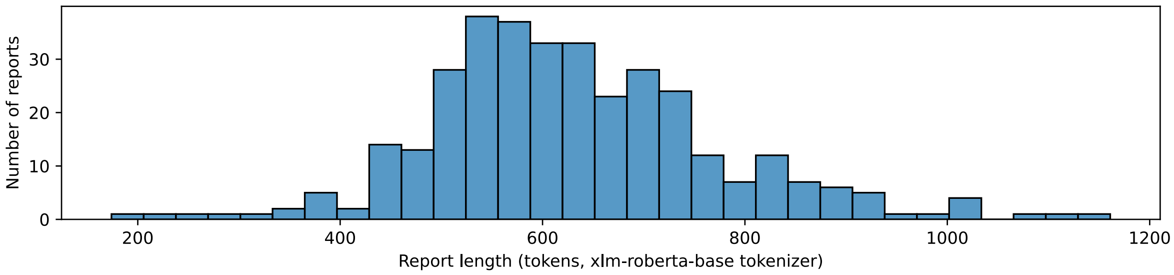

Supplementary Figure 23: Distribution of report lengths. The reports in the development dataset have a median length of 613 (95% CI: 380, 944) [min 174, max 1161] tokens with the xlm-roberta-base tokenizer.

Metric: The labels for this task are floating point values ranging from 6 to 130 mm, with 95% of values between 12 and 83 mm (ranges are based on the development data). The distribution of labels is skewed. The Robust Symmetric Mean Absolute Percentage Error Score (RSMAPES) with an epsilon of 4 mm is used to evaluate model performance. This evaluates the lesion size prediction in a diagnostically relevant manner.

Supplementary References

1. Ryan, D. P., Hong, T. S. & Bardeesy, N. Pancreatic Adenocarcinoma. *N Engl J Med* **371**, 1039–1049 (2014).
2. Schwartz, N. R. M. *et al.* Potential Cost-Effectiveness of Risk-Based Pancreatic Cancer Screening in Patients With New-Onset Diabetes. *Journal of the National Comprehensive Cancer Network* **20**, 451–459 (2022).
3. Edge, S. B. & Compton, C. C. The American Joint Committee on Cancer: the 7th Edition of the AJCC Cancer Staging Manual and the Future of TNM. *Ann Surg Oncol* **17**, 1471–1474 (2010).
4. Alves, N. *et al. The PANORAMA Study Protocol: Pancreatic Cancer Diagnosis - Radiologists Meet AI*. https://zenodo.org/doi/10.5281/zenodo.10599559 (2024) doi:10.5281/ZENODO.10599559.

Example

Label: 43

Notes: the report describes a pancreas lesion of 43 mm ("Hypovasculaire tumor in pancreascorpus en staart (43 mm)").

Anonymous sample report:
###############################
DISCLAIMER:
THIS REPORT HAS BEEN ANONYMIZED BY REPLACING PATIENT HEALTH INFORMATION WITH RANDOM SURROGATES.
ANY RESEMBLANCE TO REAL PERSONS, LIVING OR DEAD, IS PURELY COINCIDENTAL.
###############################
Gerichte vraagstelling: metastase?
Verslag:
Herbeoordeling CT d.d. 20 maart 2006. Hypovasculaire tumor in pancreascorpus
en staart (43 mm) met occlusie vena lienalis; mesenteriale en
perigastrische collateralen; vervorming portomesenterisch confluens met
doorgroei langs VMS; volledige encasement truncus coeliacus en a. lienalis
met irregulaire contour; partiele encasement art. Hepatica communis;
doorgroei richting aorticorenale plexus rechts. Enkele kleine lymfklieren
craniaal van de truncus coeliacus (6 mm) Normale grootte van de lever, met
meerdere cysten. Hypodense lesie in leversegment 5 (32 mm, 204-236).
Degeneratief skelet. Dikke galwegen. Normaal aspect galblaas. Normaal
aspect bijnieren. Normale aankleuring en grootte van de nieren met

meerdere corticale cysten. Arteriosclerose grote retroperitoneale vaten. Normaal aspect urineblaas. Normale grootte prostaat en zaadblazen. Verkalkingen in vas deferens rechts. Diverticulosis colon sigmoideum. Volumeverlies linker onderkwab. Geen pleuravocht en/of pericardvocht.
Conclusie.
Hypovasculaire tumor in pancreascorpus (43 mm) met doorgroei in de mesenteriale wortel en encasement truncus coeliacus, a. hepatica communis en art. Lienalis; doorgroei richting aorticorenale plexus rechts. Waarschijnlijk metastase in leversegment 7 (32 mm).

Task 23: Pulmonary nodule size measurement

Background: Outside a screening setting, early-stage lung cancer is generally detected after the detection of an incidental pulmonary nodule in a clinically ordered chest CT scan[1,2]. Trend analysis of the incidence of pulmonary nodules is important, because these statistics are essential in clinical decision-making and risk communication between physicians and patients with such a nodule[3]. Nodule findings are stored in unstructured radiology reports, making them difficult to extract for analysis. For large-scale trend analysis, it is unfeasible to manually analyse the reports and an automated solution would be required. Automated extraction of nodule findings could also facilitate dataset curation for the development of deep learning-based pulmonary nodule detection systems[4]. Nodule size is an important risk factor for malignancy and should therefore be extracted from the reports[5,6].

Task: Predict the largest reported diameter of pulmonary nodules described in the radiology report. When multiple sizes are described for a single lesion (e.g., the short and long axis), the size for that lesion should be averaged (e.g., 9 mm for a lesion of size 1.0 x 0.8 cm). Provide your prediction as a float.

Dataset: Hendrix et al.[1] created a dataset for nodule trend analysis with chest CT reports from two Dutch hospitals, acquired between 1 January 2008 and 31 December 2019 from Radboudumc and Jeroen Bosch Ziekenhuis. This dataset consists of a development set and an independent test set: the development set contains 1000 randomly sampled radiology reports (500 per hospital). The test set contains 200 randomly sampled reports (100 per hospital). Each report was given a label indicating (1) whether a pulmonary nodule was reported and (2) the diameter of the largest reported nodule if available (otherwise, a missing value was registered). All pulmonary nodules were included with a maximum diameter of 30 mm, regardless of morphology, type (i.e., solid, part-solid, non-solid, calcified, or perifissural), and malignancy status. For this task, all cases with a pulmonary nodule measurement were selected.

Dataset splits: The test and development datasets were kept the same as in the study of Hendrix et al.[1]. This selected 32 cases for testing and 186 cases for development. We used the development dataset for 5-fold cross-validation.

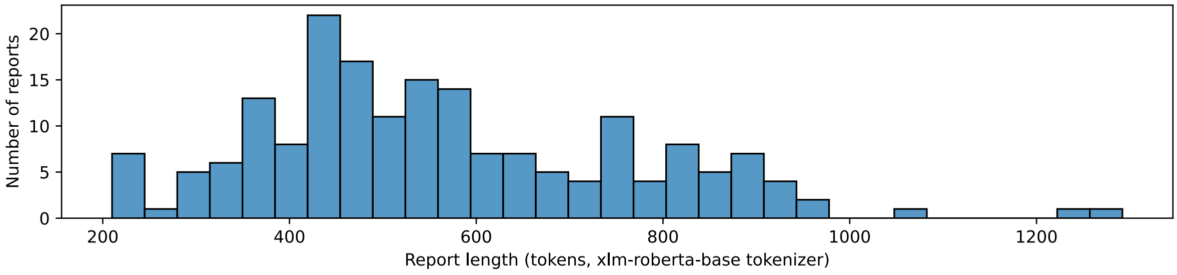

Supplementary Figure 24: Distribution of report lengths. The reports in the development dataset have a median length of 530 (95% CI: 238, 952) [min 210, max 1292] tokens with the xlm-roberta-base tokenizer.

Metric: The labels for this task are integer values ranging from 1 to 30 mm, with 95% of values between 2 and 28 mm (ranges based on the development data). The distribution of labels is moderately skewed. The Robust Symmetric Mean Absolute Percentage Error Score (RSMAPES) with an epsilon of 4 mm is used to evaluate model performance. This evaluates the lesion size prediction in a diagnostically relevant manner.

Supplementary References

1. Hendrix, W. *et al.* Trends in the incidence of pulmonary nodules in chest computed tomography: 10-year results from two Dutch hospitals. *Eur Radiol* (2023) doi:10.1007/s00330-023-09826-3.
2. Gould, M. K. *et al.* Recent Trends in the Identification of Incidental Pulmonary Nodules. *American Journal of Respiratory and Critical Care Medicine* **192**, 1208–1214 (2015).
3. Wiener, R. S., Gould, M. K., Woloshin, S., Schwartz, L. M. & Clark, J. A. What Do You Mean, a Spot?: A Qualitative Analysis of Patients' Reactions to Discussions With Their Physicians About Pulmonary Nodules. *CHEST* **143**, 672–677 (2013).
4. Gu, Y. *et al.* A survey of computer-aided diagnosis of lung nodules from CT scans using deep learning. *Computers in Biology and Medicine* **137**, 104806 (2021).
5. MacMahon, H. *et al.* Guidelines for management of incidental pulmonary nodules detected on CT images: From the Fleischner Society 2017. *Radiology* **284**, 228–243 (2017).
6. Callister, M. E. J. *et al.* British thoracic society guidelines for the investigation and management of pulmonary nodules. *Thorax* **70**, ii1–ii54 (2015).

Example

Label: 7

Notes: the report describes a nodule of 7 mm ("7 millimeter grote nodus").

Anonymous sample report:
##############################
DISCLAIMER:
THIS REPORT HAS BEEN ANONYMIZED BY REPLACING PATIENT HEALTH INFORMATION WITH RANDOM SURROGATES.
ANY RESEMBLANCE TO REAL PERSONS, LIVING OR DEAD, IS PURELY COINCIDENTAL.
##############################

Relevante voorgeschiedenis: Status na resectie sarcoom rechts parailiacaal.
Status na recidief rechterlies (27-05) status na rectumamputatie en stoma
('05). Vraagstelling: pulmonale metastasen?

Verslag:
Ter hoogte van de linker onderkwab segment 6 is aan 7 millimeter grote
nodus zichtbaar met centrale calcificatie. (Serie 5 ima 456. Radiologisch
meest passend bij granuloom. Verder met name apicaal centrilobulair
emfysemateuze veranderingen . Enkele kleine subpleurale strengetjes zonder
verdere pathologie.

Mediastinaal normaal verloop van de grote vaten. Geringe atherosclerotische veranderingen. Relatief uitgebreide coronaire calcificaties en enkele verkalkingen in de mitralisklep ring. Mediastinaal geen pathologisch vergrote klieren. Axillair geen bijzonderheden. Voorzover afgebeeld in bovenbuik normaal aspect van parenchymateuze organen behoudens een prominente cyste in de ventrale zijde van milt met een doorsnede van 38 mm. Atherosclerotische veranderingen. Degeneratieve veranderingen van afgebeelde ossale structuren zonder verdere pathologie. In de subcutane weke delen hoog links dorsaal ter hoogte van C6 bevindt zich een bolronde verdichting met een doorsnede van 30 mm.

Impressie:

Intrapulmonale nodus in de linker onderkwab met radiologische kenmerken van granuloom.
Geen aanwijzingen voor metastasen.
Bolronde verdichting subcutaan in de weke delen ter hoogte van C6 links paramediaan. Gaarne klinische informatie. Atheroomcyste?

Task 24: RECIST lesion size measurement

Background: The RECIST (Response Evaluation Criteria In Solid Tumors)[1,2] were formulated to assess the effect of cancer treatments. It involves identifying up to five target lesions in a baseline scan, measuring their axial diameter and subsequently comparing these to measurements in follow-up scans. However, when reports are stored in an unstructured format the diverse notations for this procedure pose a challenge for automated extraction of target lesion measurements. Proper measurement involves using the long-axis for lesions whereas for enlarged lymph nodes the short-axis diameter is used to track response. A Natural Language Processing (NLP) algorithm can be employed to automatically extract the target lesions from a report, determine whether the long or short axis diameter should be used based on context and report it in a standardized format (mm).

Task: predict the size in mm for up to five of the target lesions. For lymph nodes the short axis should be reported. Provide your prediction as a list of floats.

Dataset: 253 radiology reports of the Radboudumc and 144 reports of the JBZ hospitals in the Netherlands were selected from archives containing thorax-abdomen CT scans conducted between 2000-2020. Reports were selected by filtering using regular expressions, searching for mention of 'RECIST', 'target-' or 'indicator-lesions'. Patients with more than one such scan in the archive were included. Reports were manually annotated by a trained investigator (M.J.J.d.G.) under the supervision of a radiologist (E.Th.S.) to determine the lesion sizes.

Dataset splits: 30% of patients were randomly selected for the test set (119 cases) and the remaining 70% of patients (278 cases) were used for model development with 5-fold cross-validation.

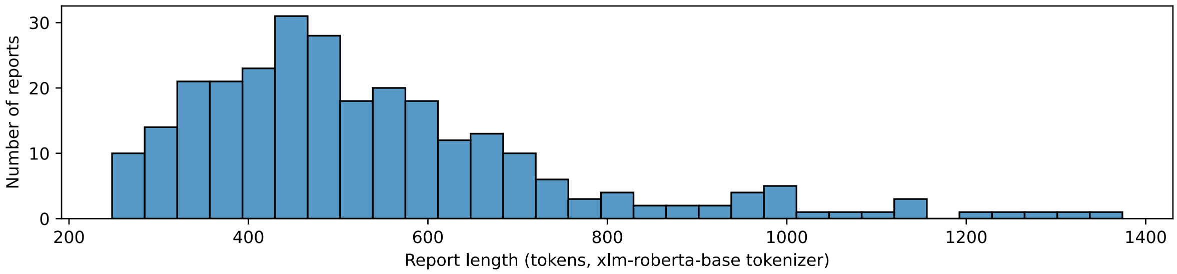

Supplementary Figure 25: Distribution of report lengths. The reports in the development dataset have a median length of 485 (95% CI: 268, 1127) [min 248, max 1374] tokens with the xlm-roberta-base tokenizer.

Metric: The labels for this task are floating point values ranging from 4 to 166 mm, with 95% of values between 6 and 94 mm (ranges based on the development data). The distribution of labels is skewed. Since the task is the same for each of the five lesions, we pool the predictions and labels for all lesions. The Robust Symmetric Mean Absolute Percentage Error Score (RSMAPES) with an epsilon of 4 mm is used to evaluate model performance. This evaluates the lesion size prediction in a diagnostically relevant manner.

Supplementary References

1. Eisenhauer, E. A. *et al.* New response evaluation criteria in solid tumours: Revised RECIST guideline (version 1.1). *European Journal of Cancer* **45**, 228–247 (2009).

2. Schwartz, L. H. *et al.* RECIST 1.1—Update and clarification: From the RECIST committee. *European Journal of Cancer* **62**, 132–137 (2016).

Example

Label: 40, 32, 14, 18, 22

Notes: the report describes multiple lymph nodes, for which the short axis should be reported: (1) Axillair links 68 x 40 mm → 40, (2) Axillair rechts 58 x 32 mm → 32, (3) Mediastinaal station 4R 27 x 14 mm → 14, (4) Precavaal 29 x 18 mm → 18, (5) Inguinaal links 36 x 22 mm → 22.

Anonymous sample report:
################################
DISCLAIMER:
THIS REPORT HAS BEEN ANONYMIZED BY REPLACING PATIENT HEALTH INFORMATION WITH RANDOM SURROGATES.
ANY RESEMBLANCE TO REAL PERSONS, LIVING OR DEAD, IS PURELY COINCIDENTAL.
################################

--Addendum start--
Target lesies: 12-06 Axillair links 68 x 40 mm (IMA 8-34) Axillair rechts 58 x 32 mm (IMA 3-38) Mediastinaal station 4R 27 x 14 mm (IMA 3-28) Precavaal 29 x 18 mm (IMA 8-104) Inguinaal links 36 x
22 mm (IMA 4-168) Inguinaal rechts 42 x 24 mm (IMA 4-172) SPD 5810 Non-target lesies: prominente
ring van Waldeyer, ongewijzigd craniocaudale afmeting van de milt bedraagt 11 cm Extranodaal: geen
Overige bevindingen: zie oorspronkelijk verslag
--Addendum einde--

Vraagstelling: Evaluatie tov CT 2 januari Evaluatie na 2 kuren chemotherapie

CT thorax abdomen, gescand na toediening van jodiumhoudend contrast intraveneus. Er is vergeleken met onderzoek van 5 maart en eerder.

Verslag:
CT-thorax en abdomen met intraveneus contrast. Ter correlatie de PET/CT
van dezelfde datum. Ter vergelijking de CT van 5 maart 2020.

Thorax.
Beiderzijds axillair conglomeraat van pathologisch vergrote lymfklieren,
met korte as diameter stand 35 mm rechts en 37 mm links. Tevens vergrote
lymfklieren mediastinaal, twee van 14 mm op level 4 rechts. Tevens
beiderzijds perihilair vergrote klieren en laag cervicaal enkele
opvallende lymfklieren.
Gedilateerde atria, verwijde truncus pulmonalis en prominente aorta
ascendens.
Coronaire sclerose. Focale gecalcificeerde atherosclerose in de aortaboog.
Scan in expiratie fase, waarbij matig beoordeelbaarheid van het
longparenchym.

Bronchuswandverdikkingen en bronchiectasieen bij bronchopathie met versterkte interstitiele tekening en matglas.
Pulmonale laesies, met name rechts; subpleuraal 9 mm (9-101) en perifissuraal 6 mm (7-123) en 11 mm (11-159), meest waarschijnlijk lymfeklieren. Tevens ongewijzigde nodi van 5 mm (8-173), 7 mm (11-86) en 9 mm (11-189).
Inhomogene schildklier bij bekende calcificatie rechts, niet vergroot.

Abdomen.
Multipele opvallende lymfklieren abdominaal, waaronder in de leverhilus van 11 mm (3-86) en precavaal van 16 mm (8-104).
Multipele vergrote lymfklieren inguinaal en in de obturatorloge, met korte as diameter tot 19 mm links en de 24 mm rechts (4-168).
Bekende hyperperfusie in segment 6 van de lever. Slanke intrahepatische galwegen. Indruk van een heterogene lever, echter suboptimale scan fase ter beoordeling van de lever. Een en ander berust mogelijk nog op wisselende perfusie.
Bekend verwijde ductus choledochus, bij status na cholecystectomie?
Symmetrische aankleuring van de nieren. Bekende corticale intrekking rechts (9-193). Slank verzamelsysteem. Slanke bijnieren.
Normaal aspect van de milt. Geen splenomegalie.
Induratie van het mesenteriale vetweefsel.
Voorts normaal aspect van de tractus digestivus.
Atherosclerotische afwijkingen van de abdominale aorta en iliacaalvaten.
Normaal aspect van de blaas. Geen vrij vocht.

Skelet en weke delen.
Degeneratief aspect van het skelet. Geen ossale haarden.
Enkele intracutane noduli in de bekkenregio.

Impressie:

Multipele (sterk) vergrote lymfklieren verspreid over thorax en abdomen, met name beiderzijds axillair en inguinaal. Mogelijk in kader van progressie van ziekte bij chronische lymfatische leukemie.

Autorisatiedatum:
9 juni Contrastmiddel: Optiray 300: 100 cc

Task 25: Anonymization

Background: Anonymization is an important first step in processing medical data to be used in further research. To protect patient and doctor's privacy, personally identifiable information should be removed and/or replaced with non-identifiable information. Detecting identifiable information can be difficult and ambiguous, and it is very time-consuming to do manually. Accurate anonymization models can improve the speed and scale of access to privacy-sensitive medical data.

Task: predict (sequences of) tokens that are considered personally identifiable information. The classes to predict are dates (e.g., "12-3-2004"), person names (e.g., "Joeran Bosma" or "JSB"), report identifiers (such as T-numbers used in pathology, e.g., "T12-345678"), places (hospitals and patient-specific places, e.g., "Radboudumc, Nijmegen" or "France"), personally identifying numbers (such as telephone numbers, patient identifiers, e.g. "06-12345678" or "012345"), clinical trial names (e.g., "4M trial"), hospital accreditation numbers (e.g., "M123"), times (e.g., "12:34"), and patient ages (e.g., "86"). Provide your prediction as a list of strings, where there is one label for each provided token, and the strings being:

17. "B-<DATUM>" for the first, or "I-<DATUM>" for subsequent date tokens
18. "B-<PERSOON>" for the first, or "I-<PERSOON>" for subsequent person name tokens
19. "B-<RAPPORT_ID>" for the first, or "I-<RAPPORT_ID>" for subsequent report identifier tokens
20. "B-<PLAATS>" for the first, or "I-<PLAATS>" for subsequent place tokens
21. "B-<PHINUMMER>" for the first, or "I-<PHINUMMER>" for subsequent personally identifying number tokens
22. "B-<STUDIE_NAAM>" for the first, or "I-<STUDIE_NAAM>" for subsequent clinical trial name tokens
23. "B-<ACCREDATIE_NUMMER>" for the first, or "I-<ACCREDATIE_NUMMER>" for subsequent hospital accreditation number tokens
24. "B-<TIJD>" for the first, or "I-<TIJD>" for subsequent time tokens
25. "B-<LEEFTIJD>" for the first, or "I-<LEEFTIJD>" for subsequent patient age tokens
26. "O" for tokens without any of the above

The report is provided as a list of tokens, split using the `dragon_prep.ner.ner_tokenizer` tokenizer.

Dataset: Reports were sourced from two hospitals:

1. Radboudumc.
   a. 1600 reports (1574 patients) were sampled randomly from patients with a thorax-abdomen or thorax CT scan at Radboudumc between 1 January 2008 and 31 December 2019. The reports were annotated semi-automatically, by processing the reports with a tool developed in-house (i.e., a rule-based system) followed by manually verifying and correcting the annotations. 65 duplicate reports were excluded to prevent overlap of reports between the dataset splits, resulting in 1535 included cases (1514 patients).
   b. Basal cell carcinoma pathology reports. 13183 cases (6555 patients) were selected from patients who underwent a skin cancer biopsy between 1 January 2003 and 31 December 2022 at Radboudumc with the diagnostic code for BCC. This dataset was constructed as part of the work by D.G.[1]. 756 cases (685 patients) were sampled and semi-automatically annotated by J.S.B., by processing the reports with a tool developed in-house (i.e., a rule-based system) followed by manually verifying and correcting the annotations.

c. Prostate pathology reports. 12,437 consecutive cases (10,402 patients) were selected from patients who underwent histopathological evaluation of prostate tissue between 1 January 2012 and 12 February 2024 at Radboudumc. 413 cases (412 patients) were randomly sampled and semi-automatically annotated by J.L., by processing the reports with a tool developed in-house (i.e., a rule-based system) followed by manually verifying and correcting the annotations.
d. Prostate pathology procedure reports. 1293 consecutive cases (1180 patients) were selected from patients who underwent an MR-guided prostate biopsy between 1 January 2014 and 31 December 2020 at Radboudumc. 515 cases (498 patients) were randomly sampled and semi-automatically annotated by J.S.B., by processing the reports with a tool developed in-house (i.e., a rule-based system) followed by manually verifying and correcting the annotations. 6 duplicate reports were excluded to prevent overlap of reports between the dataset splits, resulting in 509 included cases (492 patients).
e. Lung pathology reports. 1019 consecutive cases (902 patients) were selected from patients suspected of having non-small cell lung cancer between 1 January 2016 and 31 December 2022 at Radboudumc. 284 cases (273 patients) were randomly sampled and semi-automatically annotated by two trained investigators (J.L. and M.Ste.), by processing the reports with a tool developed in-house (i.e., a rule-based system) followed by manually verifying and correcting the annotations.

2. Antoni van Leeuwenhoek Ziekenhuis.
    a. 7581 consecutive cases (5017 patients) were selected from patients who received a prostate histopathological evaluation between 1 January 1995 and 17 February 2023 at Antoni van Leeuwenhoek Ziekenhuis. 462 cases (441 patients) were randomly sampled and manually annotated by a trained investigator (J.S.B.).
    b. 9766 consecutive cases (7841 patients) were selected from patients who had a suspicion of prostate cancer (elevated PSA levels, and/or abnormal digital rectal examination findings, and/or lower urinary tract symptoms) between 1 January 2012 and 17 February 2023 at Antoni van Leeuwenhoek Ziekenhuis. 273 cases (269 patients) were randomly sampled. Additionally, 250 consecutive cases (250 patients) between 14 October 2015 and 3 March 2016 were selected. 9 cases were selected in both sets and only included once. This included 514 cases (498 patients), which were manually annotated a trained investigator (J.S.B.).

These sources were combined to include 4385 cases (4206 patients). Each report is stored as full-word token sequences, along with sequences of beginning, inside, and outside (BIO) tags[2] annotating each word as belonging to an identifiable piece of information. Any further sub-word tokenization is left to the challenge participants.

Dataset splits: 30% of patients were randomly selected for the test set (1307 cases) and the remaining 70% of patients (3078 cases) were used for model development with 5-fold cross-validation.

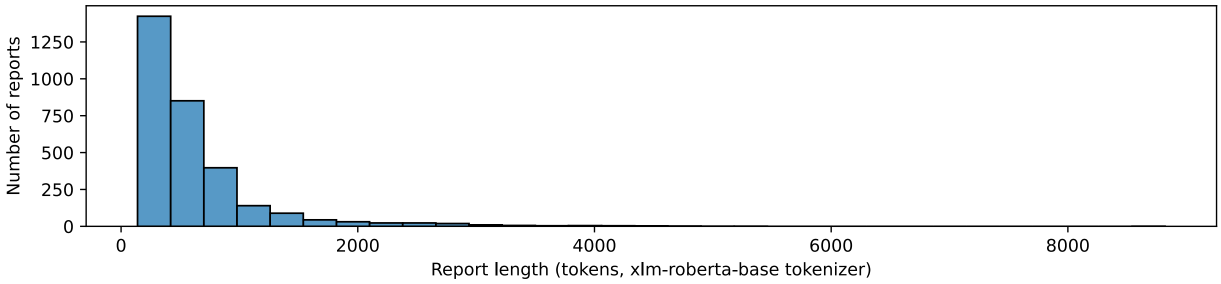

Supplementary Figure 26: Distribution of report lengths. The reports in the development dataset have a median length of 462 (95% CI: 188, 2382) [min 139, max 8822] tokens with the xlm-roberta-base tokenizer.

Metric: Predicted BIO tag sequences are converted back to complete entities before evaluation. The macro F1 metric is used to evaluate the predictions. This metric only counts predictions as correct when they perfectly overlap with ground truth annotations.

Supplementary References

1. Geijs, D. J. *et al.* Detection and subtyping of basal cell carcinoma in whole-slide histopathology using weakly-supervised learning. *Medical Image Analysis* **93**, 103063 (2024).
2. Ramshaw, L. A. & Marcus, M. P. Text Chunking using Transformation-Based Learning. Preprint at http://arxiv.org/abs/cmp-lg/9505040 (1995).

Example

Visual:

##############################

DISCLAIMER:

THIS REPORT HAS BEEN ANONYMIZED BY REPLACING PATIENT HEALTH INFORMATION WITH RANDOM SURROGATES.

ANY RESEMBLANCE TO REAL PERSONS, LIVING OR DEAD, IS PURELY COINCIDENTAL.

##############################

↵

SAMENVATTING: Patholoog: Violet Marinus Datum ontvangst: 2 okt Datum antwoord : 4/10/2024 Aard materiaal :
<PERSOON> <DATUM> <DATUM>

hersenen Klinische gegevens: Verkrijgingswijze resectie Klinische gegevens verdenking long metastase

intracerebraal links parafalcien Vraagstelling aard van het weefsel. Longarts: G. Van Veen Bethesda Materiaal voor
<PERSOON> <PLAATS>

biobank Nee Inzending I tumor Ter informatie: dit verslag is elektronisch aangemaakt middels een

spraakherkenningsysteem en elektronisch geautoriseerd. CONCLUSIE: excisie klinisch tumor, intracerebraal links

parafalcien: beeld passend bij een metastase van een niet-kleincellig carcinoom, met immunohistochemisch

aankleuringspatroon passend bij adenocarcinoom met primaire origine in de long. 4 okt 2024 AANVULLING (Lbu):
•<DATUM> •<PERSOON>

PD-L1 immunohistochemisch positief (>50%). ALK rearrangement immunohistochemisch negatief. Voor DNA

mutatieanalysen, zie T 24-817623. Voor fusiegenanalysen, zie T24-817631. MACROSCOPIE: 2 10 2024 Jaar I: vers
•<RAPPORT-ID> •<RAPPORT-ID> •<DATUM>

ontvangen, fragment voor NEOt waarna op formaline. Macro volgt. Jbo 3/10/2024 In formaline. I: onregelmatig,
•<PERSOON>
•<DATUM>

elastisch, deels beige deels bruine biopt van maximaal 2,2 cm x 1,9 cm x 1,6 cm. Aan een zijde is het fragment

afgekapseld met een dun vlies, waaronder een donkerblauwe verkleuring. In 4 lamellen. T.i. 3c. Hja MICROSCOPIE:
•<PERSOON>

hersenweefsel, met hierin een deels vitale tumor, opgebouwd uit in confluerende velden gelegen epithelioide cellen

met overwegend ruim eosinofiel cytoplasma en vergrote soms sterk polymorfe kern, deels met prominente nucleoli.

Hierbij op enkele plaatsen suggestie van buisvorming. Deze tumorcellen kleuren aan in de CK7 en TTF1 kleuring.

04.10 AANVULLING (Lbu); PD-L1 (22C3 DAKO) toont immunohistochemisch membraneuze, diffuse aankleuring in ca
•<DATUM> •<PERSOON>

80% van de tumorcellen. ALK-rearrangement immunohistochemisch negatief. Aanvullende bevindingen telefonisch

medegedeeld aan behandelend logarts op 04.10, 13u45. ISO: Het laboratorium voor pathologie is geaccrediteerd
•<DATUM>
•<TIJD>

volgens ISO 15189 RvA_TESTEN_M123. Voor de moleculaire analyses wordt deels gebruik gemaakt van faciliteiten
•<ACCREDATIE_...

van Genoomdiagnostiek Groningen onder ISO 15189 accreditatie M321. Moleculair microbiologisch onderzoek wordt
•<PLAATS> •<ACCREDATIE_...

uitbesteed aan Laboratorium Medische Microbiologie onder ISO 15189 accreditatie M456. Bij niet geaccrediteerde
•<ACCREDATIE_...

testen wordt dit expliciet vermeld.

Label tokenized:
['O', 'O', 'O', 'O', 'O', 'O', 'O', 'O', 'O', 'O', 'O', 'O', 'O', 'O', 'O', 'O', 'O', 'O', 'O', 'O', 'O', 'O', 'O', 'O', 'O',
'O', 'O', 'O', 'O', 'O', 'O', 'O', 'O', 'O', 'O', 'O', 'O', 'O', 'O', 'O', 'O', 'O', 'O', 'O', 'O', 'O', 'O', 'O', 'O', 'O', 'O',
'O', 'O', 'O', 'O', 'O', 'O', 'O', 'O', 'O', 'O', 'O', 'O', 'O', 'O', 'O', 'O', 'O', 'O', 'O', 'O', 'O', 'O', 'O', 'O', 'O', 'O',
'O', 'O', 'O', 'O', 'O', 'O', 'O', 'O', 'O', 'O', 'O', 'O', 'O', 'O', 'O', 'O', 'O', 'B-<PERSOON>', 'I-<PERSOON>',
'O', 'O', 'O', 'B-<DATUM>', 'I-<DATUM>', 'O', 'O', 'O', 'B-<DATUM>', 'I-<DATUM>', 'I-<DATUM>', 'I-
<DATUM>', 'I-<DATUM>', 'O', 'O', 'O', 'O', 'O', 'O', 'O', 'O', 'O', 'O', 'O', 'O', 'O', 'O', 'O', 'O', 'O', 'O', 'O',
'O', 'O', 'O', 'O', 'O', 'O', 'B-<PERSOON>', 'I-<PERSOON>', 'I-<PERSOON>', 'I-<PERSOON>', 'B-<PLAATS>',
'O', 'O', 'O', 'O', 'O', 'O', 'O', 'O', 'O', 'O', 'O', 'O', 'O', 'O', 'O', 'O', 'O', 'O', 'O', 'O', 'O', 'O', 'O', 'O', 'O', 'O',
'O', 'O', 'O', 'O', 'O', 'O', 'O', 'O', 'O', 'O', 'O', 'O', 'O', 'O', 'O', 'O', 'O', 'O', 'O', 'O', 'O', 'O', 'O', 'O', 'O', 'O',
'O', 'O', 'O', 'O', 'O', 'B-<DATUM>', 'I-<DATUM>', 'I-<DATUM>', 'O', 'O', 'B-<PERSOON>', 'O', 'O', 'O', 'O',
'O', 'O', 'O', 'O', 'O', 'O', 'O', 'O', 'O', 'O', 'O', 'O', 'O', 'O', 'O', 'O', 'O', 'O', 'O', 'B-<RAPPORT-ID>', 'I-

<RAPPORT-ID>', 'I-<RAPPORT-ID>', 'I-<RAPPORT-ID>', 'O', 'O', 'O', 'O', 'O', 'B-<RAPPORT-ID>', 'I-<RAPPORT-ID>', 'I-<RAPPORT-ID>', 'I-<RAPPORT-ID>', 'O', 'O', 'O', 'B-<DATUM>', 'I-<DATUM>', 'I-<DATUM>', 'O', 'O', 'O', 'O', 'O', 'O', 'O', 'O', 'O', 'O', 'O', 'O', 'O', 'O', 'O', 'O', 'B-<PERSOON>', 'B-<DATUM>', 'I-<DATUM>', 'I-<DATUM>', 'I-<DATUM>', 'I-<DATUM>', 'O', 'O', 'O', 'O', 'O', 'O', 'O', 'O', 'O', 'O', 'O', 'O', 'O', 'O', 'O', 'O', 'O', 'O', 'O', 'O', 'O', 'O', 'O', 'O', 'O', 'O', 'O', 'O', 'O', 'O', 'O', 'O', 'O', 'O', 'O', 'O', 'O', 'O', 'O', 'O', 'O', 'O', 'O', 'O', 'O', 'O', 'O', 'O', 'O', 'O', 'O', 'O', 'O', 'O', 'O', 'O', 'O', 'O', 'O', 'B-<PERSOON>', 'O', 'O', 'O', 'O', 'O', 'O', 'O', 'O', 'O', 'O', 'O', 'O', 'O', 'O', 'O', 'O', 'O', 'O', 'O', 'O', 'O', 'O', 'O', 'O', 'O', 'O', 'O', 'O', 'O', 'O', 'O', 'O', 'O', 'O', 'O', 'O', 'O', 'O', 'O', 'O', 'O', 'O', 'O', 'O', 'O', 'O', 'O', 'O', 'O', 'O', 'O', 'O', 'O', 'O', 'O', 'O', 'O', 'B-<DATUM>', 'I-<DATUM>', 'I-<DATUM>', 'O', 'O', 'B-<PERSOON>', 'O', 'O', 'O', 'O', 'O', 'O', 'O', 'O', 'O', 'O', 'O', 'O', 'O', 'O', 'O', 'O', 'O', 'O', 'O', 'O', 'O', 'O', 'O', 'O', 'O', 'O', 'O', 'O', 'O', 'O', 'O', 'O', 'O', 'O', 'O', 'O', 'O', 'O', 'O', 'B-<DATUM>', 'I-<DATUM>', 'I-<DATUM>', 'O', 'B-<TIJD>', 'I-<TIJD>', 'I-<TIJD>', 'O', 'O', 'O', 'O', 'O', 'O', 'O', 'O', 'O', 'O', 'O', 'O', 'O', 'O', 'O', 'O', 'B-<ACCREDATIE_NUMMER>', 'I-<ACCREDATIE_NUMMER>', 'O', 'O', 'O', 'O', 'O', 'O', 'O', 'O', 'O', 'O', 'O', 'O', 'O', 'B-<PLAATS>', 'O', 'O', 'O', 'O', 'B-<ACCREDATIE_NUMMER>', 'I-<ACCREDATIE_NUMMER>', 'O', 'O', 'O', 'O', 'O', 'O', 'O', 'O', 'O', 'O', 'O', 'O', 'O', 'O', 'B-<ACCREDATIE_NUMMER>', 'I-<ACCREDATIE_NUMMER>', 'O', 'O', 'O', 'O', 'O', 'O', 'O', 'O', 'O', 'O']

Anonymous sample report:
################################
DISCLAIMER: THIS REPORT HAS BEEN ANONYMIZED BY REPLACING PATIENT HEALTH INFORMATION WITH RANDOM SURROGATES.
ANY RESEMBLANCE TO REAL PERSONS, LIVING OR DEAD, IS PURELY COINCIDENTAL.
################################
SAMENVATTING: Patholoog: Violet Marinus Datum ontvangst: 2 okt Datum antwoord : 4/10/2024 Aard materiaal : hersenen Klinische gegevens: Verkrijgingswijze resectie Klinische gegevens verdenking long metastase intracerebraal links parafalcien Vraagstelling aard van het weefsel. Longarts: G. Van Veen Bethesda Materiaal voor biobank Nee Inzending I tumor Ter informatie: dit verslag is elektronisch aangemaakt middels een spraakherkenningsysteem en elektronisch geautoriseerd. CONCLUSIE: excisie klinisch tumor, intracerebraal links parafalcien: beeld passend bij een metastase van een niet-kleincellig carcinoom, met immunohistochemisch aankleuringspatroon passend bij adenocarcinoom met primaire origine in de long. 4 okt 2024 AANVULLING (Lbu): PD-L1 immunohistochemisch positief (>50%). ALK rearrangement immunohistochemisch negatief. Voor DNA mutatieanalysen, zie T 24-817623. Voor fusiegenanalysen, zie T24-817631. MACROSCOPIE: 2 10 2024 Jaar I: vers ontvangen, fragment voor NEOt waarna op formaline. Macro volgt. Jbo 3/10/2024 In formaline. I: onregelmatig, elastisch, deels beige deels bruine biopt van maximaal 2,2 cm x 1,9 cm x 1,6 cm. Aan een zijde is het fragment afgekapseld met een dun vlies, waaronder een donkerblauwe verkleuring. In 4 lamellen. T.i. 3c. Hja MICROSCOPIE: hersenweefsel, met hierin een deels vitale tumor, opgebouwd uit in confluerende velden gelegen epithelioide cellen met overwegend ruim eosinofiel cytoplasma en vergrote soms sterk polymorfe kern, deels met prominente nucleoli. Hierbij op enkele plaatsen suggestie van buisvorming. Deze tumorcellen kleuren aan in de CK7 en TTF1 kleuring. 04.10 AANVULLING (Lbu); PD-L1 (22C3 DAKO) toont immunohistochemisch membraneuze, diffuse aankleuring in ca 80% van de tumorcellen. ALK-rearrangement immunohistochemisch negatief. Aanvullende bevindingen telefonisch medegedeeld aan behandelend logarts op 04.10, 13u45. ISO: Het laboratorium voor pathologie is geaccrediteerd volgens ISO 15189 RvA_TESTEN_M123. Voor de moleculaire analyses wordt deels gebruik gemaakt van faciliteiten van Genoomdiagnostiek Groningen onder ISO 15189 accreditatie M321. Moleculair microbiologisch onderzoek wordt

uitbesteed aan Laboratorium Medische Microbiologie onder ISO 15189 accreditatie M456. Bij niet geaccrediteerde testen wordt dit expliciet vermeld.

Anonymous sample report tokenized:

['#', 'DISCLAIMER', ':', 'THIS', 'REPORT', 'HAS', 'BEEN', 'ANONYMIZED', 'BY', 'REPLACING', 'PATIENT', 'HEALTH', 'INFORMATION', 'WITH', 'RANDOM', 'SURROGATES', '.', 'ANY', 'RESEMBLANCE', 'TO', 'REAL', 'PERSONS', ',', 'LIVING', 'OR', 'DEAD', ',', 'IS', 'PURELY', 'COINCIDENTAL', '.', '#', 'SAMENVATTING', ':', 'Patholoog', ':', 'Violet', 'Marinus', 'Datum', 'ontvangst', ':', '2', 'okt', 'Datum', 'antwoord', ':', '4', '/', '10', '/', '2024', 'Aard', 'materiaal', ':', 'hersenen', 'Klinische', 'gegevens', ':', 'Verkrijgingswijze', 'resectie', 'Klinische', 'gegevens', 'verdenking', 'long', 'metastase', 'intracerebraal', 'links', 'parafalcien', 'Vraagstelling', 'aard', 'van', 'het', 'weefsel', '.', 'Longarts', ':', 'G', '.', 'Van', 'Veen', 'Bethesda', 'Materiaal', 'voor', 'biobank', 'Nee', 'Inzending', 'I', 'tumor', 'Ter', 'informatie', ':', 'dit', 'verslag', 'is', 'elektronisch', 'aangemaakt', 'middels', 'een', 'spraakherkenningsysteem', 'en', 'elektronisch', 'geautoriseerd', '.', 'CONCLUSIE', ':', 'excisie', 'klinisch', 'tumor', ',', 'intracerebraal', 'links', 'parafalcien', ':', 'beeld', 'passend', 'bij', 'een', 'metastase', 'van', 'een', 'niet', '-', 'kleincellig', 'carcinoom', ',', 'met', 'immunohistochemisch', 'aankleuringspatroon', 'passend', 'bij', 'adenocarcinoom', 'met', 'primaire', 'origine', 'in', 'de', 'long', '.', '4', 'okt', '2024', 'AANVULLING', '(', 'Lbu', ')', ':', 'PD', '-', 'L', '1', 'immunohistochemisch', 'positief', '(', '>', '50%', ')', '.', 'ALK', 'rearrangement', 'immunohistochemisch', 'negatief', '.', 'Voor', 'DNA', 'mutatieanalysen', ',', 'zie', 'T', '24', '-', '817623', '.', 'Voor', 'fusiegenanalysen', ',', 'zie', 'T', '24', '-', '817631', '.', 'MACROSCOPIE', ':', '2', '10', '2024', 'Jaar', 'I', ':', 'vers', 'ontvangen', ',', 'fragment', 'voor', 'NEOt', 'waarna', 'op', 'formaline', '.', 'Macro', 'volgt', '.', 'Jbo', '3', '/', '10', '/', '2024', 'In', 'formaline', '.', 'I', ':', 'onregelmatig', ',', 'elastisch', ',', 'deels', 'beige', 'deels', 'bruine', 'biopt', 'van', 'maximaal', '2', ',', '2', 'cm', 'x', '1', ',', '9', 'cm', 'x', '1', ',', '6', 'cm', '.', 'Aan', 'een', 'zijde', 'is', 'het', 'fragment', 'afgekapseld', 'met', 'een', 'dun', 'vlies', ',', 'waaronder', 'een', 'donkerblauwe', 'verkleuring', '.', 'In', '4', 'lamellen', '.', 'T', '.', 'i', '.', '3', 'c', '.', 'Hja', 'MICROSCOPIE', ':', 'hersenweefsel', ',', 'met', 'hierin', 'een', 'deels', 'vitale', 'tumor', ',', 'opgebouwd', 'uit', 'in', 'confluerende', 'velden', 'gelegen', 'epithelioide', 'cellen', 'met', 'overwegend', 'ruim', 'eosinofiel', 'cytoplasma', 'en', 'vergrote', 'soms', 'sterk', 'polymorfe', 'kern', ',', 'deels', 'met', 'prominente', 'nucleoli', '.', 'Hierbij', 'op', 'enkele', 'plaatsen', 'suggestie', 'van', 'buisvorming', '.', 'Deze', 'tumorcellen', 'kleuren', 'aan', 'in', 'de', 'CK', '7', 'en', 'TTF', '1', 'kleuring', '.', '04', '.', '10', 'AANVULLING', '(', 'Lbu', ')', ';', 'PD', '-', 'L', '1', '(', '22', 'C', '3', 'DAKO', ')', 'toont', 'immunohistochemisch', 'membraneuze', ',', 'diffuse', 'aankleuring', 'in', 'ca', '80%', 'van', 'de', 'tumorcellen', '.', 'ALK', '-', 'rearrangement', 'immunohistochemisch', 'negatief', '.', 'Aanvullende', 'bevindingen', 'telefonisch', 'medegedeeld', 'aan', 'behandelend', 'logarts', 'op', '04', '.', '10', ',', '13', 'u', '45', '.', 'ISO', ':', 'Het', 'laboratorium', 'voor', 'pathologie', 'is', 'geaccrediteerd', 'volgens', 'ISO', '15189', 'RvA', '_', 'TESTEN', '_', 'M', '123', '.', 'Voor', 'de', 'moleculaire', 'analyses', 'wordt', 'deels', 'gebruik', 'gemaakt', 'van', 'faciliteiten', 'van', 'Genoomdiagnostiek', 'Groningen', 'onder', 'ISO', '15189', 'accreditatie', 'M', '321', '.', 'Moleculair', 'microbiologisch', 'onderzoek', 'wordt', 'uitbesteed', 'aan', 'Laboratorium', 'Medische', 'Microbiologie', 'onder', 'ISO', '15189', 'accreditatie', 'M', '456', '.', 'Bij', 'niet', 'geaccrediteerde', 'testen', 'wordt', 'dit', 'expliciet', 'vermeld', '.']

Task 26: Medical terminology recognition

Background: Radiology reports contain a lot of terminology that is meant to communicate observations from radiological imaging results. The reports are available to patients, who have a hard time making sense of this terminology. By automatically detecting medical terminology that we can provide laymen-friendly explanations for, patient understanding of the reports can be improved[1].

Task: predict (sequences of) tokens that are considered medical terminology. Provide your prediction as a list of strings, where there is one label for each provided token, and the strings being:

27. "B-MENTION" for the first, or "I-MENTION" for subsequent medical terminology tokens
28. "O" otherwise

The report is provided as a list of tokens, split using the `dragon_prep.ner.ner_tokenizer` tokenizer.

Dataset: 250 manually annotated pseudonymized reports, randomly sampled from the Radboudumc CT thorax/abdomen archive. Annotation was performed by a trained investigator (K.D.). The reports were sampled such that they all fit inside 512 tokens using the `bert-base-multilingual-cased` tokenizer. Each report is stored as a sequence of full-word tokens, along with a sequence of beginning, inside, and outside (BIO) tags[2] annotating each word. Any further subword tokenization is left to the user.

Dataset splits: 30% of patients were randomly selected for the test set (75 cases) and the remaining 70% of patients (175 cases) were used for model development with 5-fold cross-validation.

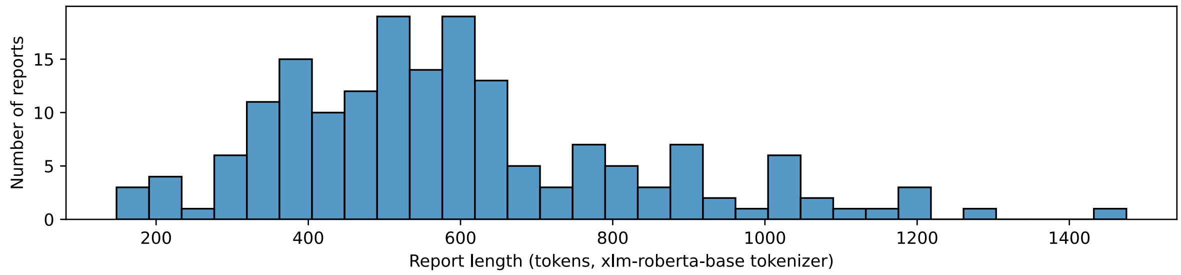

Supplementary Figure 27: Distribution of report lengths. The reports in the development dataset have a median length of 551 (95% CI: 215, 1187) [min 148, max 1475] tokens with the xlm-roberta-base tokenizer.

Metric: Predicted BIO tag sequences are converted back to complete entities before evaluation. The F1 metric is used to evaluate the predictions. This metric only counts predictions as correct when they perfectly overlap with ground truth annotations.

Supplementary References

1. Dercksen, K. & de Vries, A. P. SNOMEDCT Entity Recognition in Dutch Radiology Reports using Weak Labeling. *To be published* (2022).
2. Ramshaw, L. A. & Marcus, M. P. Text Chunking using Transformation-Based Learning. Preprint at http://arxiv.org/abs/cmp-lg/9505040 (1995).

Example

Visual:

##############################

DISCLAIMER: THIS REPORT HAS BEEN ANONYMIZED BY REPLACING PATIENT HEALTH INFORMATION WITH RANDOM SURROGATES. ANY RESEMBLANCE TO REAL PERSONS, LIVING OR DEAD, IS PURELY COINCIDENTAL.

##############################

Medische gegevens: status na hemihepatectomie rechts in verband met metastasen coloncarcinoom. CT-thorax en abdomen
•MENTION •MENTION •MENTION •MENTION

gescand volgens routine protocol na intraveneuze contrasttoediening van 50 ml Xenetix 300 en orale contrastvoorbereiding.
•MENTION •MENTION •MENTION

Vergeleken wordt met de CT van 23 nov. Axillair en mediastinaal: geen pathologisch grote klieren. Ongewijzigde kliergrootte
•MENTION •MENTION •MENTION •MENTION

ventrale mediastinum. Intra pulmonaal: bekende ongewijzigde pleurale verdikking en verkalkingen. Atelectase basale
•MENTION •MENTION •MENTION •MENTION •MENTION •MENTION
•MENTION

bovenkwab links. Intra-abdominaal: Status na hemihepatectomie rechts en low anteriorresectie. Kleine bekende ongewijzigde
•MENTION •MENTION •MENTION •MENTION

(sinds 23.11.2014) intrahepatisch hypodensiteiten IMA 75 en 90. Geen nieuwe lesies verdacht voor recidief. Ongewijzigd
•MENTION •MENTION •MENTION •MENTION

aspect milt, vergrote bijnier rechts, bekende niercysten, uitgebreide vaatcalcificaties, wandstandige plaque vorming, pancreas.
•MENTION •MENTION •MENTION •MENTION •MENTION •MENTION •MENTION

Retroperitoneaal geen pathologisch vergrote klieren. Ossaal: uitgebreide degeneratieve veranderingen. Vlekkige tekening van
•MENTION •MENTION •MENTION •MENTION
•MENTION

de ossale structuren, ongewijzigd beeld.
•MENTION

Conclusie: Ongewijzigd beeld lever en linker vergrote bijnier bij status na hemihepatectomie rechts en low anteriorresectie bij
•MENTION •MENTION •MENTION •MENTION

coloncarcinoom.
•MENTION

Label tokenized: ["O", "O", "O", "O", "O", "O", "O", "O", "O", "O", "O", "O", "O", "O", "O", "O", "O",
"O", "O", "O", "O", "O", "O", "O", "O", "O", "O", "O", "O", "O", "O", "O", "O", "O", "O", "O", "O", "O",
"O", "O", "O", "O", "O", "O", "O", "O", "O", "O", "O", "O", "O", "O", "O", "O", "O", "O", "O", "O", "O",
"O", "O", "O", "O", "O", "O", "O", "O", "O", "O", "O", "O", "O", "O", "O", "O", "O", "O", "O", "O", "O",
"O", "O", "O", "O", "O", "O", "O", "O", "O", "O", "O", "O", "O", "O", "O", "B-MENTION", "O", "O", "O",
"O", "B-MENTION", "B-MENTION", "O", "O", "O", "O", "O", "B-MENTION", "O", "O", "O", "O", "O",
"B-MENTION", "O", "O", "O", "O", "B-MENTION", "I-MENTION", "O", "B-MENTION", "O", "O", "O",
"O", "O", "O", "O", "O", "O", "O", "O", "B-MENTION", "O", "B-MENTION", "O", "O", "B-MENTION",
"O", "B-MENTION", "O", "O", "O", "B-MENTION", "B-MENTION", "O", "B-MENTION", "I-MENTION",
"O", "O", "O", "B-MENTION", "O", "O", "B-MENTION", "O", "B-MENTION", "B-MENTION", "B-
MENTION", "O", "O", "B-MENTION", "I-MENTION", "I-MENTION", "O", "O", "O", "B-MENTION", "O",
"O", "O", "B-MENTION", "O", "O", "O", "O", "O", "O", "O", "O", "O", "O", "O", "O", "B-MENTION", "B-
MENTION", "O", "O", "O", "O", "O", "O", "O", "B-MENTION", "O", "O", "B-MENTION", "O", "O", "O",
"B-MENTION", "O", "O", "B-MENTION", "O", "O", "O", "B-MENTION", "O", "O", "B-MENTION", "O",
"B-MENTION", "B-MENTION", "I-MENTION", "O", "B-MENTION", "O", "B-MENTION", "O", "B-
MENTION", "O", "B-MENTION", "O", "B-MENTION", "O", "O", "B-MENTION", "O", "O", "O", "O", "O",

"O", "B-MENTION", "I-MENTION", "O", "O", "O", "O", "O", "O", "O", "O", "B-MENTION", "O", "O", "O", "B-MENTION", "O", "O", "O", "B-MENTION", "O", "O", "O", "B-MENTION", "O", "B-MENTION", "O"]

Anonymous sample report:
##############################
DISCLAIMER: THIS REPORT HAS BEEN ANONYMIZED BY REPLACING PATIENT HEALTH INFORMATION WITH RANDOM SURROGATES. ANY RESEMBLANCE TO REAL PERSONS, LIVING OR DEAD, IS PURELY COINCIDENTAL.
##############################

Medische gegevens: status na hemihepatectomie rechts in verband met metastasen coloncarcinoom. CT-thorax en abdomen gescand volgens routine protocol na intraveneuze contrasttoediening van 50 ml Xenetix 300 en orale contrastvoorbereiding. Vergeleken wordt met de CT van 23 nov. Axillair en mediastinaal: geen pathologisch grote klieren. Ongewijzigde kliergrootte ventrale mediastinum. Intra pulmonaal: bekende ongewijzigde pleurale verdikking en verkalkingen. Atelectase basale bovenkwab links. Intra-abdominaal: Status na hemihepatectomie rechts en low anteriorresectie. Kleine bekende ongewijzigde (sinds 23.11.2014) intrahepatisch hypodensiteiten IMA 75 en 90. Geen nieuwe lesies verdacht voor recidief. Ongewijzigd aspect milt, vergrote bijnier rechts, bekende niercysten, uitgebreide vaatcalcificaties, wandstandige plaque vorming, pancreas. Retroperitoneaal geen pathologisch vergrote klieren. Ossaal: uitgebreide degeneratieve veranderingen. Vlekkige tekening van de ossale structuren, ongewijzigd beeld.

Conclusie: Ongewijzigd beeld lever en linker vergrote bijnier bij status na hemihepatectomie rechts en low anteriorresectie bij coloncarcinoom.

Anonymous sample report tokenized:
["#", "DISCLAIMER", ":", "THIS", "REPORT", "HAS", "BEEN", "ANONYMIZED", "BY", "REPLACING", "PATIENT", "HEALTH", "INFORMATION", "WITH", "RANDOM", "SURROGATES", ".", "ANY", "RESEMBLANCE", "TO", "REAL", "PERSONS", ",", "LIVING", "OR", "DEAD", ",", "IS", "PURELY", "COINCIDENTAL", ".", "#", "Medische", "gegevens", ":", "status", "na", "hemihepatectomie", "rechts", "in", "verband", "met", "metastasen", "coloncarcinoom", ".", "CT", "-", "thorax", "en", "abdomen", "gescand", "volgens", "routine", "protocol", "na", "intraveneuze", "contrasttoediening", "van", "50", "ml", "Xenetix", "300", "en", "orale", "contrastvoorbereiding", ".", "Vergeleken", "wordt", "met", "de", "CT", "van", "23", "nov", ".", "Axillair", "en", "mediastinaal", ":", "geen", "pathologisch", "grote", "klieren", ".", "Ongewijzigde", "kliergrootte", "ventrale", "mediastinum", ".", "Intra", "pulmonaal", ":", "bekende", "ongewijzigde", "pleurale", "verdikking", "en", "verkalkingen", ".", "Atelectase", "basale", "bovenkwab", "links", ".", "Intra", "-", "abdominaal", ":", "Status", "na", "hemihepatectomie", "rechts", "en", "low", "anteriorresectie", ".", "Kleine", "bekende", "ongewijzigde", "(", "sinds", "23", ".", "11", ".", "2014", ")", "intrahepatisch", "hypodensiteiten", "IMA", "75", "en", "90", ".", "Geen", "nieuwe", "lesies", "verdacht", "voor", "recidief", ".", "Ongewijzigd", "aspect", "milt", ",", "vergrote", "bijnier", "rechts", ",", "bekende", "niercysten", ",", "uitgebreide", "vaatcalcificaties", ",", "wandstandige", "plaque", "vorming", ",", "pancreas", ".", "Retroperitoneaal", "geen", "pathologisch", "vergrote", "klieren", ".", "Ossaal", ":", "uitgebreide", "degeneratieve", "veranderingen", ".", "Vlekkige", "tekening", "van", "de", "ossale", "structuren", ",", "ongewijzigd", "beeld", ".", "Conclusie", ":", "Ongewijzigd", "beeld", "lever", "en", "linker", "vergrote", "bijnier", "bij", "status", "na", "hemihepatectomie", "rechts", "en", "low", "anteriorresectie", "bij", "coloncarcinoom", "."]

Task 27: Prostate biopsy sampling

Background: MR-guided prostate biopsies offer higher accuracy in detecting clinically significant prostate cancer compared to the more traditional ultrasound-guided biopsies. Radiologists write reports in which they describe how satisfactory the biopsy was performed. For each biopsy, the radiologist describes the location and evaluates whether the sampled tissue is representative of the targeted lesion. Analyzing locations and representativeness allows for connecting pathological outcomes with radiological outcomes. The corresponding lesion can be reidentified in the MRI scan based on the location. The pathological outcomes can be used as a reference for the radiological predictions for representative biopsies. Conversely, for unrepresentative or uncertain biopsies, the pathological outcomes can be incorporated with the necessary nuance. However, performing this task manually requires specialized knowledge and is tedious for thousands of cases.

Task: For each biopsy, predict which group of words describes the location of that biopsy and whether the lesion sampling was representative, not representative, or whether this was ambiguous. Provide your prediction as a list of lists of strings, where there is one or multiple labels for each provided token, and the strings being:

29. "B-{i}-locatie naald" for the first, or "I-{i}-locatie naald" for subsequent needle location tokens of the {i}-th needle
30. "B-{i}-representatief" for the first, or "I-{i}-representatief" for subsequent tokens indicating the needle was representative
31. "B-{i}-ambigu" for the first, or "I-{i}- ambigu" for subsequent tokens indicating the needle was ambiguous
32. "B-{i}-niet representatief" for the first, or "I-{i}-niet representatief" for subsequent tokens indicating the needle was not representative
33. "O" for tokens without any of the above

The {i} should be the number of the needle, from "1", "2", "3", "4", "5", or "6". The report is provided as a list of tokens, split using the `dragon_prep.ner.ner_tokenizer` tokenizer.

Dataset: 1293 consecutive cases (1180 patients) were selected from patients who underwent an MR-guided prostate biopsy between 1 January 2014 and 31 December 2020 at Radboudumc. 515 cases (498 patients) were randomly sampled and manually annotated by two trained investigators (C.R.N. and J.S.B.) under the supervision of an interventionalist performing MR-guided prostate biopsies (10 years of experience, C.G.O.). 20 cases were excluded, due to incomplete reports (8), not being a biopsy report (4), ambiguous reporting (1), being a duplicate report (6), or being the only report describing seven biopsies (1). This included 495 cases (478 patients). Reports were annotated at the word level using 24 labels: location, representative, not representative, or ambiguous, for each of the up to 6 biopsies. Labels can be overlapping (e.g., the same word representing multiple labels). Each report is stored as full-word token sequences, along with sequences of beginning, inside, and outside (BIO) tags[1] annotating each word as belonging to an identifiable piece of information. Any further sub-word tokenization is left to the challenge participants.

Dataset splits: 30% of patients were randomly selected for the test set (146 cases) and the remaining 70% of patients (349 cases) were used for model development with 5-fold cross-validation.

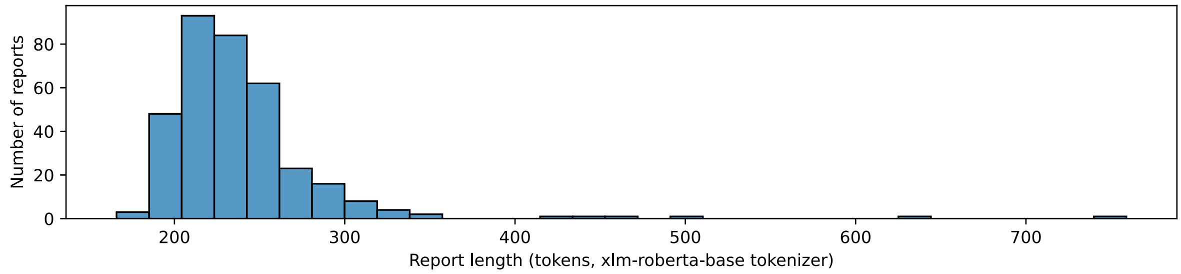

Supplementary Figure 28: Distribution of report lengths. The reports in the development dataset have a median length of 229 (95% CI: 190, 335) [min 166, max 759] tokens with the xlm-roberta-base tokenizer.

Metric: Predicted BIO tag sequences are converted back to complete entities before evaluation. The frequency of the labels is highly imbalanced and non-ordinal. The weighted F1 metric is used to evaluate the predictions. This metric only counts predictions as correct when they perfectly overlap with ground truth annotations.

Supplementary References

1. Ramshaw, L. A. & Marcus, M. P. Text Chunking using Transformation-Based Learning. Preprint at http://arxiv.org/abs/cmp-lg/9505040 (1995).

Example

Visual:

##############################

DISCLAIMER: THIS REPORT HAS BEEN ANONYMIZED BY REPLACING PATIENT HEALTH INFORMATION WITH RANDOM SURROGATES.

ANY RESEMBLANCE TO REAL PERSONS, LIVING OR DEAD, IS PURELY COINCIDENTAL.

##############################

Conform protocol werden er 1 jaar na MRI-geleide cryoablatie van het rechter vesiculum MRI-geleidebiopten genomen uit de rand van de ablatie zone.

Naald 1: Mediale rand ablatiezone zaadblaasje rechts: Mogelijk te ondiep.
Location 1 — Ambiguous 1

Naald 2: Laterale rand ablatiezone zaadblaasje rechts: Mogelijk te ondiep.
Location 2 — Ambiguous 2

Naald 3: Apex prostaat/overgang zaadblaasjes mediaal: Representatief.
Location 3 — Repres. 3

De histologische uitslag wordt separaat verzonden.

Label tokenized: [["O"],

["O"], ["B-1-locatie naald"], ["I-1-locatie naald"], ["I-1-locatie naald"], ["I-1-locatie naald"], ["I-1-locatie naald"], ["O"], ["B-1-ambigu"], ["I-1-ambigu"], ["I-1-ambigu"], ["O"], ["O"], ["O"], ["O"], ["B-2-locatie naald"], ["I-2-locatie naald"], ["I-2-locatie naald"], ["I-2-locatie naald"], ["I-2-locatie naald"], ["O"], ["B-2-ambigu"], ["I-2-ambigu"], ["I-2-ambigu"], ["O"], ["O"], ["O"], ["O"], ["B-3-locatie naald"], ["I-3-locatie naald"], ["I-3-locatie naald"], ["I-3-locatie naald"], ["I-3-locatie naald"], ["I-3-locatie naald"], ["O"], ["B-3-representatief"], ["O"], ["O"], ["O"], ["O"], ["O"], ["O"], ["O"], ["O"]]

Anonymous sample report:
##############################
DISCLAIMER: THIS REPORT HAS BEEN ANONYMIZED BY REPLACING PATIENT HEALTH INFORMATION WITH RANDOM SURROGATES.
ANY RESEMBLANCE TO REAL PERSONS, LIVING OR DEAD, IS PURELY COINCIDENTAL.
##############################
Conform protocol werden er 1 jaar na MRI-geleide cryoablatie van het rechter vesiculum MRI-geleidebiopten genomen uit de rand van de ablatie zone.

Naald 1: Mediale rand ablatiezone zaadblaasje rechts: Mogelijk te ondiep.
Naald 2: Laterale rand ablatiezone zaadblaasje rechts: Mogelijk te ondiep.
Naald 3: Apex prostaat/overgang zaadblaasjes mediaal: Representatief.

De histologische uitslag wordt separaat verzonden.

Anonymous sample report tokenized:
["#", "DISCLAIMER", ":", "THIS", "REPORT", "HAS", "BEEN", "ANONYMIZED", "BY", "REPLACING", "PATIENT", "HEALTH", "INFORMATION", "WITH", "RANDOM", "SURROGATES", ".", "ANY", "RESEMBLANCE", "TO", "REAL", "PERSONS", ",", "LIVING", "OR", "DEAD", ",", "IS", "PURELY", "COINCIDENTAL", ".", "#", "Conform", "protocol", "werden", "er", "1", "jaar", "na", "MRI", "-", "geleide", "cryoablatie", "van", "het", "rechter", "vesiculum", "MRI", "-", "geleidebiopten", "genomen", "uit", "de", "rand", "van", "de", "ablatie", "zone", ".", "Naald", "1", ":", "Mediale", "rand", "ablatiezone", "zaadblaasje", "rechts", ":", "Mogelijk", "te", "ondiep", ".", "Naald", "2", ":", "Laterale", "rand", "ablatiezone", "zaadblaasje", "rechts", ":", "Mogelijk", "te", "ondiep", ".", "Naald", "3", ":", "Apex", "prostaat", "\/", "overgang", "zaadblaasjes", "mediaal", ":", "Representatief", ".", "De", "histologische", "uitslag", "wordt", "separaat", "verzonden", "."]

Task 28: Skin histopathology diagnosis

Background: The frequency of basal cell carcinoma (BCC) cases is putting an increasing strain on dermatopathologists. BCC is the most common type of skin cancer, and its incidence is increasing rapidly worldwide. AI can play a significant role in reducing the time and effort required for BCC diagnostics and thus improve the overall efficiency of the process.

The diagnostic procedure for BCC biopsies involves two assessments by the pathologist: first, determining the presence of BCC, and second, identifying its subtype. BCC is categorized into four main subtypes: nodular, superficial, micronodular, and infiltrative. Correctly identifying these subtypes is needed, as each requires a specific surgical approach for effective treatment.

Given this context, a large dataset of pathology reports is an invaluable place to find and label cases. It serves as a foundational resource for training AI models to recognize and differentiate between BCC subtypes from biopsy samples. The process of gathering and labeling a significant volume of BCC cases involves extracting relevant information from the unstructured text of pathology reports. The challenge with unstructured text is its susceptibility to varied interpretations, making it difficult to understand the meaning of sentences using standard text filtering strategies like regex.

To overcome this hurdle, a method should be developed that enables accurate labeling and structuring of data within the reports. The effectiveness of the method is directly linked to the ability to accurately identify the presence of BCC: the higher the sensitivity, the more data can be extracted from archives, and the higher the specificity, the higher the quality of the data.

Task: Your objective is to analyze each word in the report and split the diagnosis for each specified case, numbered from 1 to 20. The diagnoses include basal cell carcinoma, benign, or other pathologies. In cases where a block is identified with basal cell carcinoma, you are required to classify each subtype present (multiple are possible for a single block). Additionally, for each block, determine the tissue acquisition method, specifying whether it was obtained through biopsy or excision, again correlating with the block number (1 – 20). Provide your prediction as a list of lists of strings, where there is one or multiple labels for each provided token, and the strings being:

34. "B-{i}-BCC" for the first, or "I-{i}-BCC" for subsequent tokens indicating basal cell carcinoma in the {i}-th block
35. "B-{i}-Benign" for the first, or "I-{i}-Benign" for subsequent tokens indicating benign tissue in the {i}-th block
36. "B-{i}-Other" for the first, or "I-{i}-Other" for subsequent tokens indicating other pathologies in the {i}-th block
37. "B-{i}-nodular" for the first, or "I-{i}-nodular" for subsequent tokens indicating the BCC is of the nodular type in the {i}-th block
38. "B-{i}-micro-nodular" for the first, or "I-{i}-micro-nodular" for subsequent tokens indicating the BCC is of the micro-nodular type in the {i}-th block
39. "B-{i}-infiltrative" for the first, or "I-{i}-infiltrative" for subsequent tokens indicating the BCC is of the infiltrative type in the {i}-th block
40. "B-{i}-superficial" for the first, or "I-{i}-superficial" for subsequent tokens indicating the BCC is of the superficial type in the {i}-th block
41. "B-{i}-biopt" for the first, or "I-{i}-biopt" for subsequent tokens indicating the tissue was acquired as a biopsy in the {i}-th block
42. "B-{i}-excision" for the first, or "I-{i}-excision" for subsequent tokens indicating the tissue was acquired as an excision in the {i}-th block
43. "O" for tokens without any of the above

The {i} should be the block number, from “1”, “2”, “3”, “4”, “5”, “6”, “7”, “8”, “9”, “10”, “11”, “12”, “13”, “14”, “15”, “16”, “17”, “18”, “19”, or “20”. The report is provided as a list of tokens, split using the `dragon_prep.ner.ner_tokenizer` tokenizer.

Note: Keep in mind that the report contains the pathologist's conclusions, and a single report may include multiple conclusions. For instance, consider a scenario where a patient has biopsies taken from three suspicious skin locations. Each location will be referred to as a case. The report will differentiate these three cases using Roman numerals. It is possible that out of these three cases, one may be diagnosed as basal cell carcinoma, another as benign, and the third as a different pathology (among the numerous skin pathologies).

Dataset: 13183 cases (6555 patients) were selected from patients who underwent a skin cancer biopsy between 1 January 2003 and 31 December 2022 at Radboudumc with the diagnostic code for BCC. This dataset was constructed as part of the work by D.G.[1]. 756 cases (685 patients) were sampled and manually annotated by trained investigators (D.G. and J.S.B.). 132 cases were excluded due to incomplete reports (129 cases) or incomplete diagnoses (3 cases). This included 624 cases (568 patients). Reports were annotated at the word level using 180 labels: basal cell carcinoma, benign, other, nodular, micro-nodular, infiltrative, superficial, biopsy, or excision, for each of the up to 20 blocks. Labels can be overlapping (e.g., the same word representing multiple labels). Each report is stored as full-word token sequences, along with sequences of beginning, inside, and outside (BIO) tags[2] annotating each word as belonging to an identifiable piece of information. Any further sub-word tokenization is left to the challenge participants.

Dataset splits: 30% of patients were randomly selected for the test set (185 cases) and the remaining 70% of patients (439 cases) were used for model development with 5-fold cross-validation.

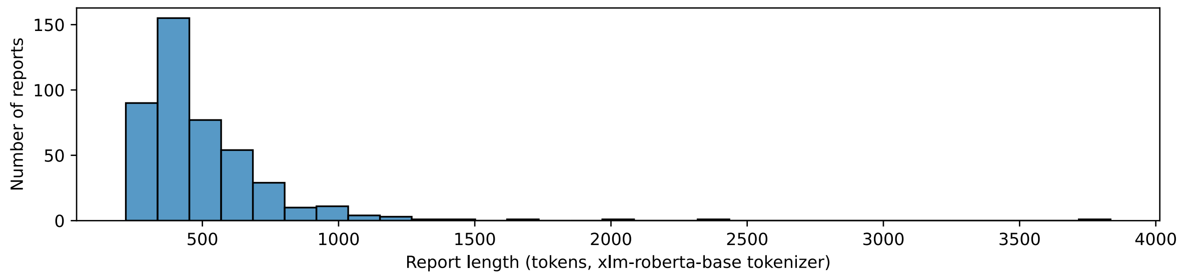

Supplementary Figure 29: Distribution of report lengths. The reports in the development dataset have a median length of 427 (95% CI: 272, 1087) [min 219, max 3834] tokens with the xlm-roberta-base tokenizer.

Metric: Predicted BIO tag sequences are converted back to complete entities before evaluation. The frequency of the labels is highly imbalanced and non-ordinal. The weighted F1 metric is used to evaluate the predictions. This metric only counts predictions as correct when they perfectly overlap with ground truth annotations.

Supplementary References

1. Geijs, D. J. *et al.* Detection and subtyping of basal cell carcinoma in whole-slide histopathology using weakly-supervised learning. *Medical Image Analysis* **93**, 103063 (2024).

2. Ramshaw, L. A. & Marcus, M. P. Text Chunking using Transformation-Based Learning. Preprint at http://arxiv.org/abs/cmp-lg/9505040 (1995).

Example

Visual:

##############################

DISCLAIMER: THIS REPORT HAS BEEN ANONYMIZED BY REPLACING PATIENT HEALTH INFORMATION WITH RANDOM SURROGATES.

ANY RESEMBLANCE TO REAL PERSONS, LIVING OR DEAD, IS PURELY COINCIDENTAL.

##############################

MACROSCOPIE: 07-03 Op formaline. I: huidstans met een doorsnede van 0,5 cm en 1 diepte van 0,2 cm. Huidoppervlak glad witgrijze aspect. Onaangesneden t.i. 1b. RGer

↵

MICROSCOPIE: Huidbiopt tot in vetweefsel met hierin een basaalcelcarcinoom met nodulaire en sprieterige groeiwijze. Deze reikt tot ca. halverwege de dermis.

↵

CONCLUSIE: Huidbiopt kaakrand links: basaalcelcarcinoom, nodulaire en sprieterige type, reikend tot halverwege de
1-biopsy 1-BCC 1-nodular 1-infilt.

dermis.

↵

HISTOLOGIE OPMERKING/GEWENST ONDERZOEK: basaalcelcarcinoom? M Bowen? Actinische keratose?

↵

UMCA IP PATH KLIN GEGEVENS: op kaakrand, thv linker mondhoek een circa 5 mm grote erythematosquameuze plaque met centraal enkele wat glanzende papels met bij dermatoscopisch onderzoek teleangiectasieen.

↵

UMCA IP PATHO VERKRIJGINGSWIJZE HISTO: biopt UMCA IP HISTOLOGIE POTJE I: 3 mm HE biopt uit afiwjking

Label tokenized: [["O"], ["B-1-biopt"], ["O"], ["O"], ["O"], ["B-1-BCC"], ["O"], ["B-1-nodular"], ["O"], ["B-1-infiltrative"], ["O"], ["O"], ["O"], ["O"], ["O"], ["O"], ["O"], ["O"], ["O"], ["O"], ["O"], ["O"], ["O"], ["O"],

["O"], ["O"], ["O"], ["O"], ["O"], ["O"], ["O"], ["O"], ["O"], ["O"], ["O"], ["O"], ["O"], ["O"], ["O"], ["O"],
["O"], ["O"], ["O"], ["O"], ["O"], ["O"], ["O"], ["O"], ["O"], ["O"], ["O"], ["O"], ["O"], ["O"], ["O"], ["O"],
["O"], ["O"], ["O"], ["O"], ["O"], ["O"], ["O"], ["O"], ["O"], ["O"], ["O"], ["O"], ["O"], ["O"], ["O"], ["O"],
["O"], ["O"], ["O"], ["O"], ["O"], ["O"], ["O"], ["O"], ["O"], ["O"]]

Anonymous sample report:
##############################
DISCLAIMER: THIS REPORT HAS BEEN ANONYMIZED BY REPLACING PATIENT HEALTH INFORMATION WITH RANDOM SURROGATES.
ANY RESEMBLANCE TO REAL PERSONS, LIVING OR DEAD, IS PURELY COINCIDENTAL.
##############################
MACROSCOPIE: 07-03 Op formaline. I: huidstans met een doorsnede van 0,5 cm en 1 diepte van 0,2 cm. Huidoppervlak glad witgrijze aspect. Onaangesneden t.i. 1b. RGer

MICROSCOPIE: Huidbiopt tot in vetweefsel met hierin een basaalcelcarcinoom met nodulaire en sprieterige groeiwijze. Deze reikt tot ca. halverwege de dermis.

CONCLUSIE: Huidbiopt kaakrand links: basaalcelcarcinoom, nodulaire en sprieterige type, reikend tot halverwege de dermis.

HISTOLOGIE OPMERKING/GEWENST ONDERZOEK: basaalcelcarcinoom? M Bowen? Actinische keratose?

UMCA IP PATH KLIN GEGEVENS: op kaakrand, thv linker mondhoek een circa 5 mm grote erythematosquameuze plaque met centraal enkele wat glanzende papels met bij dermatoscopisch onderzoek teleangiectasieen.

UMCA IP PATHO VERKRIJGINGSWIJZE HISTO: biopt UMCA IP HISTOLOGIE POTJE I: 3 mm HE biopt uit afiwjking

Anonymous sample report tokenized:
["#", "#",
"#", "#", "#", "#", "#", "#", "#", "DISCLAIMER", ":", "THIS", "REPORT", "HAS", "BEEN", "ANONYMIZED",
"BY", "REPLACING", "PATIENT", "HEALTH", "INFORMATION", "WITH", "RANDOM", "SURROGATES",
".", "ANY", "RESEMBLANCE", "TO", "REAL", "PERSONS", ",", "LIVING", "OR", "DEAD", ",", "IS",
"PURELY", "COINCIDENTAL", ".", "#", "#", "#", "#", "#", "#", "#", "#", "#", "#", "#", "#", "#", "#", "#",
"#", "#", "#", "#", "#", "#", "#", "#", "#", "#", "#", "#", "#", "#", "#", "MACROSCOPIE", ":", "07", "-",
"03", "Op", "formaline", ".", "I", ":", "huidstans", "met", "een", "doorsnede", "van", "0", ",", "5",
"cm", "en", "1", "diepte", "van", "0", ",", "2", "cm", ".", "Huidoppervlak", "glad", "witgrijze", "aspect",
".", "Onaangesneden", "t", ".", "i", ".", "1", "b", ".", "RGer", "MICROSCOPIE", ":", "Huidbiopt", "tot",
"in", "vetweefsel", "met", "hierin", "een", "basaalcelcarcinoom", "met", "nodulaire", "en",
"sprieterige", "groeiwijze", ".", "Deze", "reikt", "tot", "ca", ".", "halverwege", "de", "dermis", ".",
"CONCLUSIE", ":", "Huidbiopt", "kaakrand", "links", ":", "basaalcelcarcinoom", ",", "nodulaire", "en",
"sprieterige", "type", ",", "reikend", "tot", "halverwege", "de", "dermis", ".", "HISTOLOGIE",
"OPMERKING", "\/", "GEWENST", "ONDERZOEK", ":", "basaalcelcarcinoom", "?", "M", "Bowen", "?",
"Actinische", "keratose", "?", "UMCA", "IP", "PATH", "KLIN", "GEGEVENS", ":", "op", "kaakrand", ",",
"thv", "linker", "mondhoek", "een", "circa", "5", "mm", "grote", "erythematosquameuze", "plaque",
"met", "centraal", "enkele", "wat", "glanzende", "papels", "met", "bij", "dermatoscopisch",
"onderzoek", "teleangiectasieen", ".", "UMCA", "IP", "PATHO", "VERKRIJGINGSWIJZE", "HISTO", ":",
"biopt", "UMCA", "IP", "HISTOLOGIE", "POTJE", "I", ":", "3", "mm", "HE", "biopt", "uit", "afiwjking"]
